# Proteomic Analysis of Tears and Conjunctival Cells Collected with Schirmer Strips Using timsTOF Pro: Preanalytical Considerations

**DOI:** 10.3390/metabo12010002

**Published:** 2021-12-21

**Authors:** Murat Akkurt Arslan, Ioannis Kolman, Cédric Pionneau, Solenne Chardonnet, Romain Magny, Christophe Baudouin, Françoise Brignole-Baudouin, Karima Kessal

**Affiliations:** 1 Institut National de la Santé et de la Recherche Médicale INSERM UMR 968, CNRS UMR 7210, Institut de la Vision, IHU ForeSight, Sorbonne Université UM80, 75012 Paris, France; murat.akkurt@inserm.fr (M.A.A.); ioannis.kolman@inserm.fr (I.K.); romain.magny@inserm.fr (R.M.); cbaudouin@15-20.fr (C.B.); fbaudouin@15-20.fr (F.B.-B.); 2 Institut National de la Santé et de la Recherche Médicale INSERM, UMS PASS, Plateforme Post-Génomique de la Pitié Salpêtrière (P3S), Sorbonne Université, 75013 Paris, France; cedric.pionneau@sorbonne-universite.fr (C.P.); solenne.chardonnet@sorbonne-universite.fr (S.C.); 3 Centre Hospitalier National d’Ophtalmologie des Quinze-Vingts, INSERM-DGOS CIC 1423, IHU ForeSight, 75012 Paris, France; 4 Centre Hospitalier National d’Ophtalmologie des Quinze-Vingts, Service 3, 75012 Paris, France; 5 Ambroise Paré, Assistance Publique-Hôpitaux de Paris APHP, Service d’Ophtalmologie, Université Paris Saclay, 92100 Boulogne, France; 6 Centre Hospitalier National d’Ophtalmologie des Quinze-Vingts, Laboratoire d’Ophtalmobiologie, 75012 Paris, France; 7 Faculté de Pharmacie, Université de Paris, 75006 Paris, France

**Keywords:** proteome, tears, Schirmer strip, timsTOF Pro, signaling pathways

## Abstract

This study aimed to investigate the human proteome profile of samples collected from whole (W) Schirmer strips (ScS) and their two parts—the bulb (B) and the rest of the strip (R)—with a comprehensive proteomic approach using a trapped ion mobility mass spectrometer, the timsTOF Pro. Eight ScS were collected from two healthy subjects at four different visits to be separated into three batches, i.e., 4W, 4B, and 4R. In total, 1582 proteins were identified in the W, B, and R batches. Among all identified proteins, binding proteins (43.4%) and those with catalytic activity (42.2%) constituted more than 80% of the molecular functions. The most represented biological processes were cellular processes (31.2%), metabolic processes (20.8%), and biological regulation (13.1%). Enzymes were the most represented protein class (41%), consisting mainly of hydrolases (47.5%), oxidoreductases (22.1%), and transferases (16.7%). The bulb (B), which is in contact with the conjunctiva, might collect both tear and cell proteins and therefore promote the identification of more proteins. Processing B and R separately before mass spectrometry (MS) analysis, combined with the high data acquisition speed and the addition of ion-mobility-based separation in the timsTOF Pro, can bring a new dimension to biomarker investigations of a limited sample such as tear fluid.

## 1. Introduction

The tear film, composed of the secretions of lacrimal glands, Meibomian glands, and goblet cells, is essential for many vital functions of the ocular surface (OS), such as protection, lubrication, and nutrition [1,2]. A healthy tear film represents a barrier between the eye and the environment [3]. By coating the OS, the tear film provides an optically smooth surface, which is necessary for the refraction of light onto the retina [4,5]. Tear fluid (TF) is an important medium for the evaluation of OS disease (OSD), and it is suitable for prognostic and diagnostic purposes [6]. Despite its small volume, TF offers several advantages for biochemical analysis, biomarker discovery, and development of new drugs [5,6,7,8,9]. TF can be collected with various instruments such as microcapillary tubes, absorbent cellulose acetate filters, polyester wicks, and Schirmer strips (ScS) [10,11], the latter of which is used by clinicians to perform the Schirmer’s tear test (STT). The STT is a standard ophthalmologic test that measures tear production based on the wetted length of the strip. It is one of the most commonly used clinical tests for the evaluation of dry eye disease (DED) [12,13]. Likewise, it is relatively convenient, rapid and reliable, and therefore, despite a slight and brief tingling when placed into the conjunctival cul-de-sac, it is used more frequently than the glass capillary tube method, which requires a delicate procedure on the open eye [14]. Apart from being more convenient to collect tear samples, especially in patients with DED, the ScS offers the opportunity to collect, through the bulb (the upper part of the ScS), superficial conjunctival cells [11]. In other words, secreted proteins can be collected using the capillary method, while a combination of cellular and soluble proteins can be collected using ScS [15]. These collection methods are of major interest in identifying biomarker candidates in OSD such as DED, using powerful “omics” technologies. Indeed, tear proteomics is increasingly employed for such investigations [16,17]. In the last two decades, new insights have been achieved in tear proteomics studies using various systems such as Q-TOF or Orbitrap [18,19,20]. The first major proteomics dataset for tears was created by de Souza et al. in 2006, with the identification of 491 proteins, and in the last decade, several new proteomics approaches have been used, creating large datasets of the tear proteome by using various forms of advanced mass spectrometry (MS) [16,18,20,21,22]. These studies, revealing the datasets for the healthy tear proteome are summarized in Table 1.

Interest in MS continues to grow, and new technologies are emerging for TF analysis [27]. These more efficient techniques have promoted the reliable identification of large numbers of proteins from TF samples [28,29,30]. Thus, MS has been implemented in several studies exploring the modulation or dysregulation of proteins in OSD [15,18,31,32,33,34]. Nano-scale liquid chromatography (nano-LC), coupled to tandem MS (MS/MS), provides the improved chromatographic separation of peptides, higher sensitivity, and extended dynamic ranges to identify a large number of proteins [35,36]. Therefore, nano-LC-MS/MS has become the first choice for proteomic analysis of small samples such as TF [37]. The timsTOF Pro mass spectrometer (Bruker Daltonics, Bremen, Germany), which combines the sensitivity of trapped ion-mobility spectrometry (TIMS) and a fast quadrupole time-of-flight (Q-TOF), has been providing a large dataset for exploring the proteome of several biological fluids [38,39]. After peptide separation with nano-LC, peptides are subsequently ionized and trapped in a dual TIMS [40]. This dual TIMS technology traps ions in the first section and separates them according to their mobility in the second section [41], functioning as a third dimension of separation [39]. Thanks to the dual TIMS technique, the parallel accumulation-serial fragmentation (PASEF) acquisition method was developed, providing a duty cycle of nearly 100% (nearly no ion loss) and a high sensitivity [39]. PASEF corresponds to a mass selective release of peptide ions from TIMS devices for MS/MS at a high acquisition speed, increasing MS/MS scan rates up to 100 Hz [42,43]. In conclusion, the timsTOF Pro provides extremely high speeds and sensitivity to reach new depths in proteomics analysis using a small amount of samples.

The current study sought to perform a comprehensive tear proteomics investigation from samples collected with ScS using nanoElute ultra-high-pressure liquid chromatography (UHPLC) coupled to the timsTOF Pro. The protein composition of the various sections of the ScS can be considered to characterize the human ScS-extracted proteome (SEP) and describe in detail the signaling pathways involved.

## 2. Results

### 2.1. Number of Identified Proteins (NIPs) and Their Distributions in the Sections of the ScS 

**Figure 1:**
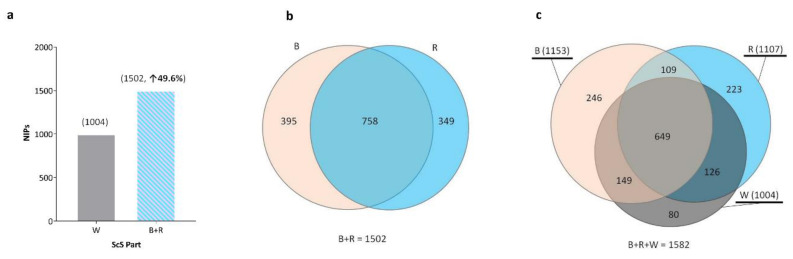
Number of identified proteins (NIPs) and their distributions in the various sections of the Schirmer strips (ScS). (**a**) Effect of processing two different sections of the ScS (B + R) on the NIPs. (**b**) Comparison of the NIPs between the two sections of the ScS and section R. (**c**) Venn diagram displaying the comparison of the NIPs in the whole strip (W), bulb (B), and the rest of the strip (R).

In the whole ScS (W), the NIPs was 1004. When the two sections of the ScS were analyzed separately, 1153 proteins were identified in the upper part (or bulb (B)) and 1107 were in the rest of the ScS (R). Therefore, processing sections B and R of the ScS as two different sample batches increased the NIPs from 1004 to 1502, for a 49.6% increase in identified proteins compared to in the entire strip (W) (Figure 1a). Of these 1502 proteins identified in sections B and R, a higher number of proteins, 758, were common, compared to 395 and 349 in sections B and the R, respectively (Figure 1b). Moreover, among the identified proteins in the 3 batches (W, B, and R), 649 proteins were common, whereas a smaller number of unique proteins, 80, 246, and 223 in W, B, and R, respectively, were identified (Figure 1c). The 20 most abundant proteins identified with the highest mean MS/MS spectral count in W, B, and R are shown in Tables S2–S4.

In total, 1582 proteins were identified by summing the 3 batches with no pre-fractionation during sample preparation. The raw data was represented in Table S1, and the MS proteomics data were deposited to the ProteomeXchange Consortium via the PRIDE partner repository with the dataset identifier PXD030334. Moreover, we compared these results with those from four proteomics studies that used advanced MS technologies in healthy human tear proteome [19,20,21,25] in order to enrich the SEP dataset. Among the 1183 proteins identified in our study, 266 were detected in study (**A**), 972 were detected in study (**B**), 487 were detected in study (**C**), and 543 were detected in study (**D**) (Figure S1). More interestingly, we identified 399 new proteins that were not identified in these studies.

### 2.2. Distribution within ScS Sections and Spectral Counts for the Previously Described Tear Proteins

The tear proteins identified in this study and previously reported in the literature for their role in OSD are presented with their spectral counts (Table 2). This classification also highlights the specific and common proteins identified in ScS batches. Some of these common proteins, such as PIP, HSPG2, and C3, were among the 20 most abundant proteins identified with the highest mean of spectral count in W, B, and R (Tables S2–S4). Interestingly, when considering clinically relevant OSD biomarkers in tears, such as lysozyme C, lactotransferrin, and lipocalin-1, our results showed that their mean spectral counts were rather similar in the three batches. However, the spectral count for another relevant OSD biomarker, serum albumin, was different among batches, with 101.7, 204.3, 67 in W, B, and R, respectively. This difference of the spectral counts between batches was also found for ALDH1A3 and α-enolase, with a higher abundance in B, for mammaglobin-B with a higher abundance in W, and HSPG2 with a higher abundance in R. Additionally, some proteins were either unique only to two batches or even just one. For instance, MUC5AC and IL-18 were detected with a higher spectral count in B compared to W but were undetected in the R section of the ScS. Finally, matrix metalloproteinase (MMP)-9 was detected only in B, with a low quantity, whereas Serpin B3 and B4 were specifically identified in R.

### 2.3. Functional Annotation Analysis in the Various Sections of the ScS

**Figure 2:**
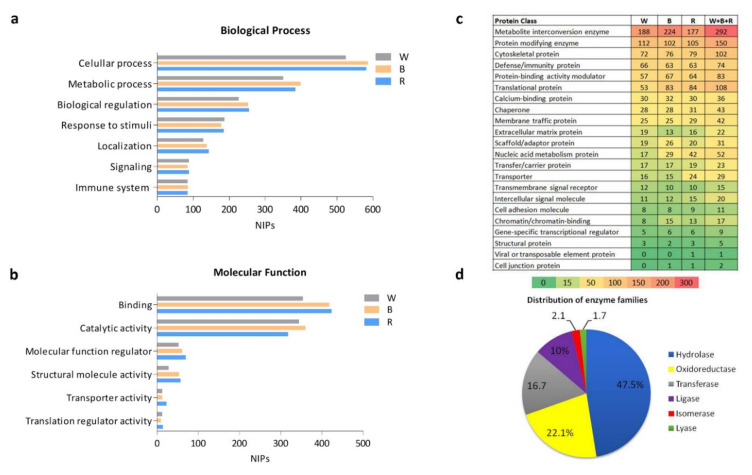
Functional analysis and structural classification of identified proteins. (**a**) Subgroups of the biological process. (**b**) Subgroups of molecular function. The NIPs illustrated in the *x*-axis show the numbers of proteins involved in (**a**,**b**). (**c**) The list and number of protein classes in each ScS section and all batches. (**d**) Distribution and classification of all identified enzymes.

Despite the several unique proteins identified in each batch (W, B, and R), no major difference was observed in biological processes or molecular functions, as shown in Figure 2a,b. Over 800 identified proteins were related to cellular processes, and 565 identified proteins were associated to binding as a molecular function. With regard to total identified proteins, cellular processes, metabolic processes, biological regulation, and responses to stimuli formed the major subgroups, with percentages of 31.3%, 20.9%, 13%, and 8.9%, respectively, of the biological processes (Table S5). Concerning molecular function, binding and catalytic activity represented the major subgroups, with percentages of 44% and 38.3%, respectively (Table S6).

In addition to biological process and molecular function classification, the protein classes in the healthy SEP are shown in Figure 2c. The metabolite interconversion enzyme class was the most represented group, with 224 in B and less in the two other parts (177 in R and 188 in W). This was followed by protein-modifying enzymes, cytoskeletal proteins, and immunity proteins. In other protein classes, the numbers of proteins in the three batches were not particularly different (Figure 2c). Interestingly, when all identified proteins from the three batches were considered, enzymes formed almost the majority (41%) of the SEP, with 480 identified proteins. Furthermore, the numbers of these enzymes were distributed differently among various protein classes, for example metabolite interconversion enzyme class (292) could include protein modifying enzymes (150), translational proteins (14), transporters (9), nucleic acid metabolism (9), and protein-binding activity modulators (6). The distribution of the sub-families of all identified enzymes are shown in Figure 2d. The largest enzyme families consisted of hydrolases (47.5%) followed by oxidoreductases (22.1%) and transferases (16.7%). Isomerase and lyase were less represented in the healthy SEP. Inversely, when the unique proteins of each batch were considered, numbering 80 in W, 246 in B, and 223 in R (Figure 1c), the differences among subclasses of molecular function, biological processes, and protein classes were more distinctive in the three batches (Figure S2). Indeed, in biological process classification, the proportion of the response to stimulus proteins, signaling, and immune system processes was higher in W (Figure S2a). As for molecular function classification, the proteins with catalytic activity formed the highest percentage (55%) in W (Figure S2b). Nevertheless, the percentage of binding proteins in W (31.7%) was lower than in B or R. Additionally, the classification of unique proteins in each batch (Figure S2c) highlighted a major representation of metabolite interconversion enzymes in B, with 61, 33 and 14 proteins in B, R, and W, respectively. Moreover, nucleic acid metabolism proteins were preferentially found in R, with 20, 8, and 1 proteins in R, B, and W, respectively. Finally, unique proteins in the protein-binding activity modulator and transporter protein classes were not identified in W.

### 2.4. Signaling Pathways in the ScS-Extracted Proteome

**Figure 3:**
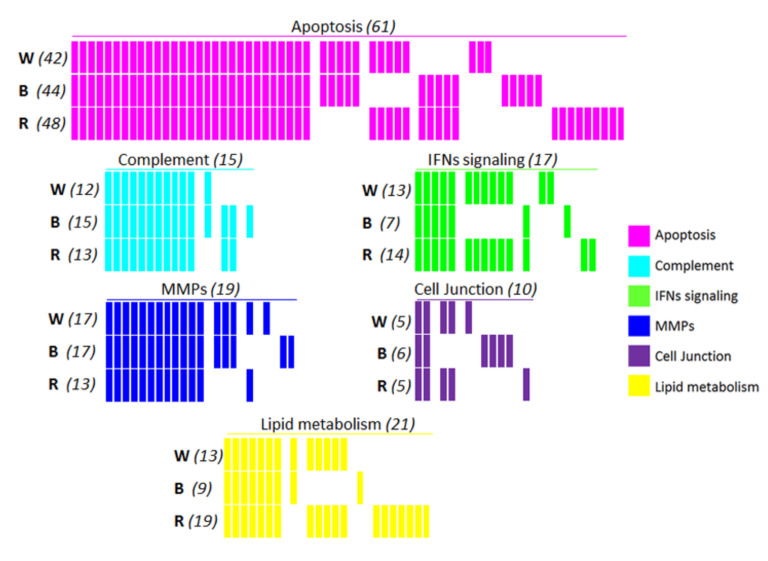
Barcodes of the major signaling pathways and NIPs in each ScS batch. The six signaling pathways were distributed as apoptosis, complement, interferons (IFNs) signaling, matrix metalloproteinases (MMPs), cell junction, and lipid metabolism in the whole strip (W), the bulb (B), and the rest (R). Each bar represents one protein. The total NIPs (W + B + R) are indicated between the bracket above each pathway barcode, and the corresponding NIPs for each batch are indicated between brackets on the left.

Six major signaling pathways, consisting of identified proteins in W, B, and R, are illustrated in Figure 3, as a barcode highlighting the overall distribution of molecular effectors for each batch. The corresponding proteins are listed in Table S7. Apoptosis was the most represented signaling pathway among these. Nearly half of the proteins involved in apoptosis (29) were common amongst the three batches. In apoptosis, 17 unique proteins, mostly related to proteasome subunits (PSM), were detected. These specific cellular effectors numbered 3 for W (PSMD5, BID, and TNFSF10), 5 for B (PSMB7, PSMD9, PSMF1, DFFA, and FNTA), and 9 for R (PSMC2, -4, and -6, PSMD1, -6, -12, PSME3, LMNB1, and STK24). In the complement cascade, the majority of the proteins were common among the three batches, representing both the alternative and classical pathways. Complement factor C1QB, a molecular effector of the classical pathway in response to antigen-antibody response, was only detected in B. Proteins involved in interferon (IFN) signaling pathways, such as Eukaryotic Translation Initiation Factors EIF4-A1, -A2, -A3, and -G1, were identified in both R and W, while EIF4E was specifically identified in R. Only one among the interferon-stimulating genes (ISGs), MX1, was specifically identified in the W batch. Among the 23 proteins involved in MMP pathways, 12 were common among the three batches, and only three unique proteins, including MMP-9, were in B. In the cell junction signaling pathway, only two proteins, nectin cell adhesion molecule 4 (PVRL4) and cadherin (CDH1), were common to the three batches, whereas more proteins were related to B, with four specifically identified, i.e., ACTN1, FLNA, PLEC, and VASP. Finally, the majority of the lipid metabolism-related proteins were identified in R, with 20 molecular effectors mostly represented by the phospholipase A2 (PLA2) family, including PLA2G2A, -G4B, -G4E, and -G4D, related to phospholipid metabolism. Only one enzyme, 3-ketodihydrosphingosine reductase (KDSR), related to de novo sphingolipid biosynthesis, was specifically identified in B. Likewise, a fatty acid metabolism effector, hydroxyacyl-CoA dehydrogenase (HADHA), was specifically detected in R.

## 3. Discussion

The importance of the biological function of TF in the protection of the OS justifies the interest in deepening our knowledge of its composition and the role of its molecular effectors in reacting to changes in the OS [63]. In this study, nano-LC-MS/MS analysis was performed using nanoElute UHPLC coupled to a timsTOF Pro mass spectrometer, which enables tunable high performance for differentiating many isobaric and isomeric molecules present within biological systems [64]. The addition of ion mobility separation to the chromatographic-mass separation increases sensitivity and reduces spectral complexity in this MS [40]. The timsTOF Pro enables the analysis of samples with a minimal sample size less than 200 ng with a sequencing speed exceeding 100 Hz [39]. Tear samples can be collected using various methods [65]. However, the ScS approach remains the most common method due to its advantages over the other methods [14]. In order to use this powerful MS technology, we sought to optimize sample preparation. Thus, we investigated the various sections of the ScS, considering the whole strip (W) and its two parts, the bulb (B), and the rest of the ScS (R). The upper section (B), in contact with the conjunctiva, has the feature of cell adhesion lacking in the remaining part (R). The healthy SEP was investigated using different parts of the ScS with the timsTOF Pro to enrich the protein dataset while revealing the differences among protein profiles in W, B, and R. Indeed, processing B and R separately increased the total NIPs, even though the protein quantity of each batch (W, B, and R) was initially normalized before the MS/MS analysis. The composition of the protein content in W should be more varied and complex than in B and R, as it includes proteins from the totality of the strip. However, new and specific proteins for each piece of the strip were identified, when the strip was cut into two pieces. Thus, factors such as the ScS area and the mechanical action during the protein elution step seem to participate in the protein composition as well as the extraction yield. This suggests that the complexity of the protein composition and the pre-analytical phases of sample preparation greatly influence the nano-LC-MS/MS data analysis. As compared to other tear proteome studies [19,20,21], the large number of proteins identified in this study has increased our knowledge and contributed to enriching the tear proteome dataset to better decipher all the functions of tears. This might also have facilitated the detection of proteins due to their lower quantities in B and R. Eluting these two regions separately and pooling them before one-run MS/MS analysis could improve protein identification. Interestingly, as previously reported, enzymes represented the largest group of proteins in the SEP. The composition of enzymes in the healthy SEP revealed that hydrolases, oxidoreductases, and transferases formed the main enzyme families [21]. In our study, the number of identified enzyme families enriched the proteome dataset, where the percentage of identified hydrolases increased from 41% to 47.5% and the percentage of oxidoreductases increased from 15% to 22.1% compared to the results of this study. Nevertheless, the percentage of transferases decreased from 27% to 16.7% [21]. Likewise, another tear enzyme family, with antioxidant enzymes such as superoxide dismutases (SOD-1, -2, and -3), glutathione peroxidases (GPX-1, -3, and -4), catalase (CAT), and glutathione reductase (GSR), is responsible for the elimination of excessive amounts of reactive oxygen species [66]. In addition to the enrichment in tear enzyme identification, several calcium-binding proteins as Annexin A and S100A family members have been identified. These enzymes have a broad range of intracellular and extracellular regulatory functions encompassing the regulation of cellular apoptosis, energy metabolism, protein phosphorylation, and inflammation, among others [67]. Interestingly, the regulation of these proteins of interest is known to be associated with OSD [1]. The specificity of this study was derived from combining the timsTOF analysis with the elution of three different areas of the ScS, revealing major common proteins as well as unique proteins identified with a single batch. Additionally, although common proteins were identified between two or three batches, they differed by their mean of spectral counts. For instance, mucin-5AC, the major mucin secreted by the conjunctival goblet cells [55], was found in both the entire ScS (W) and the proximal section (B) of the ScS, which is in contact with the conjunctival mucosa, but was not found in the distal portion (R) of the ScS, which does not contain cells and is wetted only by capillary action. The additional recovery observed from the proximal portion (B) might be explained by the relationship between the molecular weight and the membrane pore size of the ScS, where a diameter of 20–25 µm allows large proteins such as mucin-5AC (641 kDa) to be preferentially retained [45]. Thus, migration to the remaining section of the ScS (R) might be hampered. This specific identification in the whole ScS and the proximal section (B), with different spectral means, was also observed for Mucin-5A and Mucin-5B. Therefore, the STT seems to be the most convenient collection method for tear film mucins, supported by the fact that the quantity of Mucin-5AC is higher in ScS than in basal tears or eye-flush tears [54].

The different relative abundance depending on sample preparation enables improvements in protein identification, either for comprehensive proteomic studies or for routine assays such as Western blotting and ELISA. For example, myeloperoxidase (MPO), involved in inflammation, host defense, and neutrophil function [68], was specifically found in B, but not in R. Thus, the sample preparation of B alone will probably be beneficial for the enrichment of the analytical tear sample. In the report of the Tear Film Subcommittee of the TFOS DEWS II, 102 extracellular and 20 intracellular proteins were referenced [1]. Of these 20 intracellular proteins, 12 of them were found in this study. The spectral means of these 12 proteins were higher in B than in R and W. This result logically suggests that B concentrates intracellular proteins. Likewise, among the 102 extracellular proteins, albumin, lactotransferrin, complement C3, lipocalin-1 enolase-1, and annexin A2 displayed a higher spectral count in B compared to in R. It could be hypothesized that these tear proteins accumulate in B and, a lesser extent, migrate to R via capillary action.

Interestingly, the mean spectral count of the most unique proteins in B and R was higher than that of the unique proteins in W. Indeed, the mean spectral count of 80 unique proteins in W was less than 2, while several unique proteins in B and R had mean spectral counts reaching 6.7 and 19.3, respectively. This finding could suggest that the unique proteins in B and R could be more informative compared to the unique proteins in W. Despite the widespread use of MS in proteomic studies, the identification of low-abundance proteins such as cytokines remains a challenge, and multiplex technology is still used for their quantification [69,70]. This is also supported by the fact that the concentrations of cytokines in tears are very low [57]. However, the resolutive specificity of the timsTOF Pro enables the identification of these molecules. Indeed, seven cytokines, consisting of interleukins and chemokines, were identified in the various batches. The specificities of identification were also reported according to the ScS sections, such as for IL-19 in W and IL-36γ, IL-36Ra, and IL-36α, which were identified only in R. CXCL17 and IL1-RN were identified in all three batches. IL-18 was shared by both W and B, and CXCL10 was shared by both W and R.

IL-1RN, IL-18, CXCL17, and CXC10 were previously detected in a few tear proteomic studies, while IL-36α, IL-36γ, and IL-36Ra were identified only in our study, as opposed to the four other studies [19,20,21,24]. IL-36,a new member of the IL-1 cytokine family, contains IL-36α, IL-36β, IL-36γ, and IL-36Ra and plays a crucial role in inflammation and immunity [71]. The modulation of IL-36α has been reported in Sjögren’s syndrome [62]. IL-36γ induces Mucin 5AC expression [72]. The role of IL-36 members in OSD is of great interest for further investigation. These results highlight the interest to study separately the various remaining sections of the ScS for exploring cytokine modulations in OSD.

The enrichment of the SEP dataset also allows the description of the multiple signaling pathways in healthy human tears. Six major signaling pathways have been reported for the OS, and proteins involved in these pathways have been determined. These pathways involve the following: (1) apoptosis; (2) complement; (3) IFN signaling; (4) MMPs; (5) cell junction; and (6) lipid metabolism. In apoptosis pathways, more proteins were identified than in other pathways. Apoptosis, as a response to physiological stimuli to maintain homeostatic mechanisms during cellular development, aging, and injuries, is highly significant in OS changes [73,74]. Proteasome 20S and 26S subunits (PSMA/B and C/D, respectively) and proteasome subunit activator 3 (PSME3) constitute almost half of the proteins from this pathway. Additionally, the well-described apoptotic enzymes such as CASP-3/7 [74] were specifically identified in W and B. The complement system is a crucial mediator of the innate immune response, and it prevents the spread of infection to other cells and tissues through opsonization, attracting immune cells or direct lysis, removing damaged cells/tissues and preventing the development of chronic inflammation [75]. Furthermore, the protein composition linked to the complement system differs according to the collection method and the size of the palpebral fissure. Thus, it has been reported that the expression levels of these proteins are higher in tear samples collected via the capillary method in comparison to by the STT [23]. Another study demonstrated the identification of C1Q, C3, CFB, C4, C5, and C9 in closed-eye tears and only the identification of C3, CFB, and C4 in open-eye and reflex tears [76]. The methodology used in this study enriched the dataset of complement cascades by adding C1R, C1QC, C6, C4BPA, C7, C8A, CFD, CFH, and CFI complement factors. The interferon-signaling pathway is also an important cellular response in OSD, which supports further investigations to understand the regulation of these molecular effectors [60,77,78,79]. Proteins identified in interferon signaling pathways, such as eukaryotic translation-initiation factors (EIF4A-1, -2, and -3, EIF4G1, and EIF4E), important regulators of mRNA translation [80], were identified in R and W, but not in B. Additionally, the human leukocyte antigen (HLA) class I proteins (HLA-A/B/C) could be identified in each region of the strip, except for HLA-E, which is unique to R. Regarding the extracellular matrix (ECM) and cell junction, two pathways were identified according to the identified proteins in each section of the ScS. The enzymes involved in the proteolysis of the ECM, such as MMPs, play a great role in wound healing and inflammation, and their increased activity has been reported in OSD [81]. As previously reported, MMP-9 in human corneal epithelial cells correlates positively with increasing osmolarity, which is mediated by the activation of c-Jun N-terminal kinases (JNKs) and stress-activated protein kinase (SAPK) pathways [82]. Furthermore, the MMP-9 test is a valuable diagnostic tool in DED [83]. The majority of identified protein components of the MMP pathway were shared by all batches. Interestingly, MMP-9 was identified only in B, but with a very low spectral mean, which could be explained by the healthy status of the donors. This finding should guide the choice of ScS preparation for further MMP-9 investigation. Additionally, collagen alpha chain proteins such as COL6A1, COL14A1, and COL9A3 were mostly found in all batches, except for COL9A3, which was not identified in B. A comparison with previous comprehensive proteomic studies [19,20,21,24] identified new collagen alpha chain components in tears—COL6A1 and COL9A3. These molecular effectors involved in the integrity of cellular membranes in the allergic response, infectious conditions, and other OSDs [84] could provide new insight for exploring potential targets of interest.

Finally, lipids are essential components in cellular functions, signaling networks, and energy metabolism [85]. The members of this molecular family are key mediators in intercellular and intracellular processes in both inflammatory processes and cell death phenomena [86,87]. Interestingly, these bioactive lipids and their modulation were also described in studies of an in vitro DED model [88,89]. This metabolic involvement is supported by the identification of several enzymes. The secretory calcium-dependent phospholipase A2 family, including PLA2-G4B, -G4D, and -G4E, was specifically identified in R. These enzymes are involved in the inflammatory response and host antimicrobial defense and play a role in the glycerophospholipid catabolic process/phospholipid metabolic process [90]. Likewise, several other lipid metabolism pathways were identified with tear enzymes, such as glycosphingolipid metabolism and fatty acid metabolism (HADHA). De novo sphingolipid biosynthesis pathways were also identified, based on KDSR, a specific enzyme identified only in R. Among the identified molecules involved in lipid metabolism, 12 of them were newly identified in our study as opposed to the other three comprehensive tear proteomic studies that we compared [19,20,21].

Lastly, analyzing tear and cellular proteins in the various sections of the ScS increased the NIPs as well as our knowledge of the SEP. Eluting these two portions before MS analysis provides additional protein characterization information compared to eluting the entire strip. High NIPs such as enzymes and other molecular effectors might help to enrich the investigation of signaling pathway regulation and cellular processes in OS homeostasis. This proteome profile could be also slightly enriched with the use of a larger cohort.

## 4. Materials and Methods

### 4.1. Sample Collection and Processing

The study was performed according to the tenets of the Declaration of Helsinki and GCP, and written consent was obtained from all subjects after explaining the protocol and the scope of the study. The study was approved by the Ethics Committee CPP–Ile-de-France (number: 2018-A02800-55). Two healthy subjects (HSs) who participated in this study were volunteers. Two healthy volunteers were males with age 32 and 33, respectively. All ScS collected for this study reached the full length of wetness within 5 min of the sampling time. They did not wear contact lenses, used no systemic or ocular medication, had no past history of systemic or ophthalmic diseases known to alter tear production and had not undergone any ophthalmic surgeries. Both HSs in this study had to pass an ocular examination including tear break-up time (TBUT), corneal fluorescein staining (CFS), the evaluation of conjunctival hyperemia, and a STT to exclude the presence of any clinical pathology.

**Figure 4:**
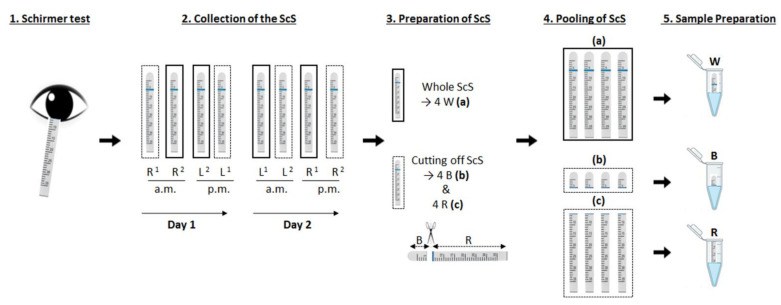
Illustrations of tear sample collection with ScS and sample processing. Eight ScS were collected from two HSs without anesthesia in 4 different sampling periods over 2 days. Two equivalent groups of the ScS were obtained by pooling them. In the first group, whole strips (W) were used; in the second group, the strips were cut to separate the bulbs (B) and the rest of the strips (R). The 4 pooled W strips (a), 4 B strips (b), and 4 R strips (c) were extracted in ammonium bicarbonate to prepare an analytical sample for protein quantification and MS analysis. R, right eye; L, left eye; a.m., in the morning; p.m., in the afternoon; ^1^, healthy subject-1; ^2^, healthy subject-2; ScS, Schirmer strips; W, the whole strip; B, the bulb; R, the rest of the strip.

The bulb (B) of the ScS (Schirmer-plus^®^, Gecis; Neung Sur Beuvron, France) was gently inserted into the lower conjunctival cul-de-sac without local anesthesia, after which the subjects closed their eyes. The ScS were collected after 5 minutes, unless the entire strip was fully saturated with tears before. Sample collection from the 2 HSs and processing is illustrated in Figure 4. For each subject, ScS samples were collected from both eyes in the morning (a.m.) and afternoon (p.m.) for two consecutive days. Collected strips were placed in a sterile plastic microtube and immediately stored at −80 °C until the analytical procedure. To batch the samples, 8 wetted strips were separated into two batches. In the first batch (**a**), the 4 whole strips were pooled (W). In the second batch, the whole ScS was cut into two parts to provide the upper part containing the bulb (B) and the remaining part (R). This homogeneous sectioning provided two batches of samples, (**b**) and (**c**), with 4 bulbs and 4 rests, respectively. As for the W samples, the ScS were also pooled. Each pooled batch (**a**–**c**) was eluted in 1 mL of 100 mM ammonium bicarbonate (AmBic; Sigma-Aldrich, St. Louis, MO, USA) containing protease inhibitor cocktail (P2714; Sigma-Aldrich, St. Louis, MO, USA), placed on an IKA^®^ VXR basic Vibrax^®^ (IKA^®^-Werke GmbH & Co. KG, Staufen, Germany) orbital shaker at 1500 motions/minute at 4 °C for 4 h. The samples were centrifuged at 14,000 rpm at 4 °C for 5 min, and the supernatants were collected. Protein quantification assay was carried out using a BCA Protein Assay Kit (Thermo Scientific, Pierce, IL, USA) and using Spark^®^ Multimode Microplate Reader (Tekan, Männedorf, Switzerland).The samples were stored at −80 °C until MS analysis.

### 4.2. Sample Preparation for Nano-LC-MS/MS Analysis

Sample preparation was performed on 10 µg of proteins for each pooled sample, adjusted to the same volume and same protein concentration. Then, dithiothreitol (#3483-12-3; Sigma-Aldrich, St. Louis, MO, USA) was added to the samples up to 5 mM as a final concentration to reduce the proteins in a 37 °C water bath for 30 min. Subsequently, alkylation was performed by adding iodoacetamide (#144-48-9; Sigma-Aldrich, St. Louis, MO, USA) up to 15 mM for 30 min in the dark and at room temperature. The samples were then subjected to two-step in-solution digestion, first by adding 200 ng of Lys-C protease (#90307; Thermo Fisher Scientific) (protease/protein mass ratio equal to 1:50) and incubating the samples for 120 min at 37 °C and then by adding 200 ng of trypsin (#90058; Thermo Fisher Scientific; protease/protein mass ratio equal to 1:50) for overnight incubation at 37 °C. Following the digestion, the automated StageTips desalting of the peptides was performed using DigestProMSi (Intavis, Cologne, Germany). The eluted peptides were dried using a vacuum centrifuge and resuspended in 0.1% formic acid (#F0507; Sigma-Aldrich, St. Louis, MO, USA) and 2% acetonitrile (#271004; Sigma-Aldrich, St. Louis, MO, USA). For each sample, 500 ng of peptides were injected in triplicate into a nanoElute UHPLC spectrometer (Bruker, Champs-sur-Marne, France) coupled to a timsTOF Pro mass spectrometer (Bruker, Champs-sur-Marne, France). The peptides were directly loaded and separated on an Aurora2 RP-C18 analytical column (25 cm, 75 μm i.d., 120 Å, 1.6 µm; IonOpticks, Fitzroy, VIC Australia) at a flow rate of 400 nL/min at 40 °C. Mobile phase A consisted of 2% acetonitrile and 0.1% formic acid, and mobile phase B consisted of 99.9% acetonitrile containing 0.1% formic acid. A 90 min elution gradient was performed by increasing mobile phase B from 0% B to 3% over 1 min, from 3% B to 15% over 56 min, then from 15% B to 23% over 21 min, and from 23% B to 32% over 13 min. MS acquisition was run in data-dependent acquisition (DDA) mode with PASEF. The accumulation time was set to 100 ms in the TIMS tunnel. The capillary voltage was set to 1.6 kV, the mass ranged from 100 to 1700 *m/z* in MS and MS/MS, and the mobility ranged from 0.6 to 1.6 cm^2^/(Vs). Dynamic exclusion was activated for ions within a mass of 0.015 *m/z* and a mobility of 0.015 cm^2^/(Vs) and released after 0.4 min. Exclusion criteria was defined as 4 times higher ion intensity of a precursor. Low-abundance precursors below the target value of 20,000 arbitrary units (a.u.) and intensity of 2500 a.u. were selected several times for PASEF-MS/MS, until the target value was reached. Parent ion selection was achieved by using a two-dimensional *m/z* and 1/k_0_ selection area filter, allowing the exclusion of singly charged ions. The total cycle time was 1.1 s with 10 PASEF cycles.

### 4.3. Nano-LC-MS/MS Data Analysis

Raw MS/MS data were analyzed with Bruker Compass Data Analysis (version 5.1) and further processed with MaxQuant software (version 1.6.8.0) for protein identification [91]. The search parameters were set as follows: mass tolerances of 25 ppm for MS1 and 40 ppm for MS2, carbamidomethyl as fixed modification, acetyl (protein N-terminal), and oxidation (on M residue) as variable modifications; up to 2 missed cleavages were allowed. Protein identifications were performed against the Uniprot Homo sapiens database. The Uniprot homo sapiens database version used was UP000005640_9606 in 2019 (https://www.uniprot.org/, accessed on 2 August 2019) with 20,672 entries on 2 August 2019, while the *p*-values of peptides and proteins were adjusted to obtain the corresponding FDRs of <1%, with a minimum of 1 peptide per protein. The semi-quantitative estimations of the protein content of the samples were performed, taking into account the numbers of matched spectra per protein. The spectral counting method was used for the comparison. This method is defined as the total number of spectra identified for a protein, and it has been accepted as a practical, label-free, semi-quantitative measure of protein abundance in proteomics studies [92].

To identify new tear proteins using a timsTOF Pro in this study, a non-supervised comparison analysis was conducted with four recent studies using advanced MS technologies, i.e., Kandhavelu, et al. [20], (B) Dor, et al. [21], Aass, et al. [20], and Ponzini, et al. [25] (Figure S1). These three studies analyzing tears from HSs were selected for their high numbers of identified proteins.

### 4.4. Functional Annotation and Signaling Pathway Network Analysis

**Figure 5:**
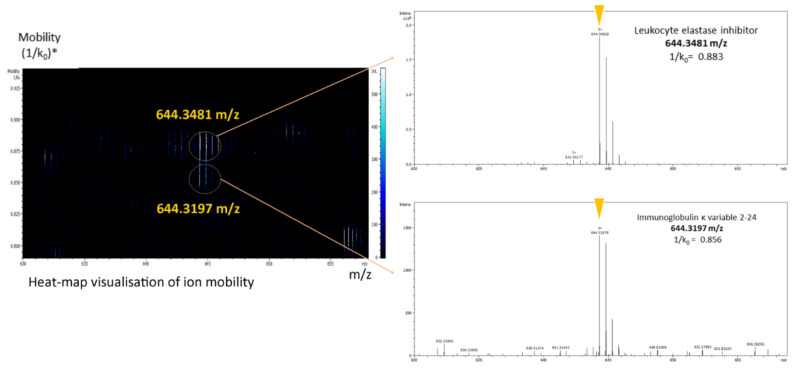
Heat-map visualization of ion mobility. The peptide ions of two different proteins with almost the same mass-to-charge ratio (*m/z*) at a single time point were differentiated by the timsTOF Pro analysis. The circled lines indicate the *m/z* and mobility positions of two precursor ions selected for fragmentation by parallel accumulation-serial fragmentation (PASEF). *; (1/k_0_), the drift time.

All identified proteins from the three groups (W, B, and R) were examined by comparing gene ontology (GO) terms. The proportions of the associated proteins in each group were obtained from the PANTHER Classification System (http://www.pantherdb.org/, accessed on 3 May 2021) [93]. Among 1582 identified proteins, Panther software could classify 1562 proteins according to their functional classification. The Reactome database was used for signaling pathway analysis (https://reactome.org/, accessed on 16 March 2021) [94]. As an example of the powerful abilities of this new-generation MS device, the timsTOF Pro, to separate different molecules with the same mass-to-charge ratio (*m/z*), Figure 5 presents how the peptide characterization was assessed by sequencing two of the targeted peptides possessing almost the same mass—leukocyte elastase inhibitor and immunoglobulin kappa variable 2-24 (644.3438 and 644.3144 *m/z* for HNSSGSILFLGR and FSGSGAGTDFTLK, respectively). These proteins were co-eluted at the same retention time and with a very similar *m/z*, but the peptides were differentiated by the timsTOF Pro, thanks to its third dimension ion-mobility feature.

## 5. Conclusions

By merging three samples corresponding to different sections of the ScS, 1582 proteins were identified in this study. Enzymes constituted the largest protein group among the identified proteins. Investigating two portions of the ScS separately enabled the identification of a number of additional proteins. Eluting sections B and R separately before analysis with this robust, highly sensitive MS device, the timsTOF Pro, might offer a holistic view of the main proteins present on the OS, from both cellular and soluble components, akin to combining a conjunctival imprint and a tear sample. The timsTOF Pro might bring a new dimension to the profiling of the tear proteome in OSD due to its unique sensitivity, enabling in-depth proteomic analysis from a small tear sample. The dataset created will contribute to the tear proteome as well as to the description and comparison of multiple signaling pathways associated with the OS.

This study may be the basis for new extensive studies on ScS specimens collected from both HSs and patients with various OSD. Collecting tear specimens with the ScS and eluting proteins from B and R separately before MS analysis might be the best method for future clinical studies of the OS proteome.

## Figures and Tables

**Table 1:** Overview of proteomics studies that have created the largest datasets for the healthy human tear proteome using LC-MS/MS from 2006 to 2020. NIPs, number of identified proteins; HSs, healthy subjects; MS, mass spectrometry; FDR, false discovery rate.

Group, Year	Goal	Tear Sampling Method	Sample Preparation	MS Technology	Protein Identification Criteria	NIPs in HSs
de Souza et al., Genome Biol., 2006 [22]	Characterization of the protein content of the human tear fluid from a HSs	Microcapillary method	With pre-fractionation of proteins with one-dimensional SDS-PAGE (13 fractions) or without (in-solution digestion of the whole samples)	Hybrid linear ion trap–Fourier-transform (LTQ-FT) and linear ion trap-Orbitrap (LTQ-Orbitrap)	Two peptides with Mascot scores of >35, Two peptides with Mascot scores of >27 (*p* ≤ 0.01), or one peptide with an Mascot score of >54 (*p* ≤ 0.0001), when MS3 was performed	491
Zhou et al., J. Proteomics, 2012 [18]	Analysis of the human tear proteome from HSs	Schirmer strips	Offline SCX fractionation of peptides (6 fractions)	TripleTOF 5600 system	FDR <1% for peptides	1543
Aass et al., Anal. Biochem., 2015 [19]	Optimizing extraction method from Schirmer strips to study the tear proteome	Schirmer strips	Offline SCX fractionation of peptides (16 fractions)	LTQ-Orbitrap XL hybrid	Peptide and protein with FDRs of <1% (high) and 5% (relaxed)	1526
Kandhavelu et al., J. Proteomics, 2016 [20]	Comparison of tear proteins in control and fungal keratitis patients	Capillary method	N-linked glycoprotein enrichment or one-dimensional SDS-PAGE pre-fractionation of proteins (26 fractions)	LTQ-Orbitrap Velos Pro	One peptide with an FDR of <5%	1873
Dor et al., Exp. Eye Res., 2019 [21]	Characterization of healthy human tear protein composition	Schirmer strips	Off-gel electrophoresis of peptides (12 fractions)	LTQ-Orbitrap Velos Pro TripleTOF 5600+ in SWATH-MS mode	Two peptides with FDRs of <1%	1351
Nättinen et al., Trans. Vis. Sci. Tech., 2020 [23]	Investigation of protein profile differences between capillary and Schirmer strip tear fluid samples	Schirmer strips	No pre-fractionation (in-solution digestion of whole samples)	TripleTOF 5600 + in SWATH-MS mode	FDR <1%	908
		Capillary method				404
Hua et al., BMC Ophthalmol., 2020 [24]	Quantification of potential candidate biomarkers for HSV-1 epithelial keratitis	Microcapillary method	No pre-fractionation (in-solution digestion of whole samples)	LTQ-Orbitrap XL	FDR <1%	949
Ponzini et al., Int. J. Mol. Sci., 2021 [25]	Demonstration of feasibility of single-tear quantitative proteomics	Capillary method	No pre-fractionation (in-solution digestion of whole samples)	Orbitrap fusion	One peptide with an FDR of <1.0%	932
Zysset-Burri et al., Inv. Ophthalmol. Vis. Sci., 2021 [26]	Exploring the interplay between the ocular surface microbiome and the tear proteome	Schirmer strips	With pre-fractionation of (5 factions) proteins with one-dimensional SDS-PAGE	QExactive HF	FDR <1%	2172

**Table 2:** List of identified proteins previously described in ocular surface disease. The mean spectral count and standard deviation (SD) of each protein in the whole strip (W), the bulb (B), and the rest (R) of the strip are presented. The proteins are classified as those common in all batches (1), two batches (2), or one batch (3) and are ranked according to their mean of spectra in W. *, proteins reported in dry eye disease studies. Accession number refers to UniProt identification.

Accession Number	Protein Name	W	B	R
		Mean ± SD		
(1) Common in all batches				
P12273	Prolactin-induced protein [1]	142.3 ± 14	108 ± 15.4	121.7 ± 2.5
P98160	Heparan sulfate proteoglycan 2 [1,29]	113.3 ± 5.5	56.3 ± 4.9	123.7 ± 1.5
P61626	Lysozyme C * [6,29,44]	111.3 ± 6.7	104 ± 4.6	108.7 ± 5.5
P01876	Ig alpha-1 chain C region [6,29]	107 ± 2.6	61.7 ± 3.2	74 ± 4.4
P02768	Serum albumin * [6,45]	101.7 ± 2.1	204.3 ± 8.1	67 ± 4
P02788	Lactotransferrin * [29,44]	100.7 ± 5.6	130.3 ± 7.1	101.7 ± 3.5
P01024	Complement C3 [22,44]	72.7 ± 3.2	69.7 ± 1.5	62.7 ± 2.1
Q9UGM3	Salivary agglutinin [1,44]	50.7 ± 4	48 ± 2	54.3 ± 0.6
P31025	Lipocalin-1 * [6,29,44]	45.3 ± 2.1	50 ± 3.6	36.7 ± 2.3
P00352	Retinal dehydrogenase 1 [23,44]	35.3 ± 2.1	46 ± 4	24.7 ± 2.1
O75556	Mammaglobin-B [1,44]	35.3 ± 0.6	18 ± 1	6.3 ± 1.2
P06733	Alpha-enolase [1,44]	31.3 ± 1.2	52 ± 4.6	27.3 ± 0.6
P02787	Serotransferrin [46]	24.7 ± 3.1	59 ± 4	26.7 ± 1.5
P07900	Heat shock protein HSP 90-alpha [23,47]	22.3 ± 1.5	8 ± 2.6	24.3 ± 1.5
P04083	Annexin A1 [23,44]	22.3 ± 0.6	24.7 ± 0.6	18 ± 1
P06702	Protein S100-A9 [1]	19 ± 1	28.7 ± 2.1	22 ± 1
P07858	Cathepsin B [1]	18.3 ± 1.5	19.7 ± 1.5	22 ± 1.7
P47895	Aldehyde dehydrogenase family 1 member A3 [23,48]	18 ± 2.6	23.7 ± 1.5	6.3 ± 2.1
P00738	Haptoglobin [1]	18 ± 2	19 ± 1.7	14 ± 0
P30740	Serpin B1 [22,23]	15.7 ± 2.1	24.3 ± 1.2	8.7 ± 3.2
P05109	Protein S100-A8 [1]	15.7 ± 0.6	22.3 ± 3.2	22 ± 2.6
P25311	Zinc-alpha-2-glycoprotein [1]	12.7 ± 1.5	14.3 ± 1.5	16.3 ± 1.5
P07355	Annexin A2 [23,44]	12.7 ± 1.2	36.7 ± 3.5	25 ± 1
P18510	Interleukin 1 receptor antagonist protein [49]	9.7 ± 1.5	12.3 ± 1.5	7 ± 1
Q8WUM4	Programmed cell death 6-interacting protein [50,51]	9 ± 0	2.7 ± 0.6	5 ± 1.7
P23528	Cofilin-1 [1]	8.7 ± 3.8	15.3 ± 2.1	8.7 ± 2.1
P07476	Involucrin [52]	6 ± 1	3 ± 1.7	20.3 ± 2.3
P01023	Alpha-2-macroglobulin [22,52]	5.3 ± 1.5	15.7 ± 2.3	7.7 ± 1.2
Q6UXB2	C-X-C motif chemokine ligand 17 [53]	1 ± 1	0.7 ± 1.2	1 ± 1
(2) Common to two batches				
P98088	Mucin-5AC [44,54,55]	10.3 ± 0.6	54.3 ± 3.8	0
Q14116	Interleukin 18 [56]	0.7 ± 0.6	2 ± 1	0
P02778	C-X-C motif chemokine ligand 10 [57]	2 ± 0	0	0.3 ± 0.6
(3) Unique to one batch				
Q9UHD0	Interleukin 19 [58]	0.7 ± 1.2	0	0
P14780	Matrix metallopeptidase 9 * [49,59,60]	0	0.3 ± 0.6	0
P29508	Serpin B3 [61]	0	0	23.7 ± 2.5
P48594	Serpin B4 [61]	0	0	12 ± 1
Q9UHA7	Interleukin 36 alpha [62]	0	0	0.3 ± 0.6

## Data Availability

The mass spectrometry proteomics data have been deposited to the ProteomeXchange Consortium via the PRIDE partner repository with the dataset identifier PXD030334 (accessed on 12 December 2021).

## Supplementary Materials

The following are available online at https://www.mdpi.com/article/10.3390/metabo12010002/s1. Table S1: Raw data of all identified proteins provided by the ScS extract of the 3 batches (W + B + R); Table S2: List of identified proteins with the highest mean of the spectral count in the whole strip (W); Table S3: List of identified proteins with the highest mean of the spectral count in the ScS bulb (B); Table S4: List of identified proteins with the highest mean of the spectral count in the rest (R) of the ScS; Table S5: Biological processes associated with identified proteins in the whole strip (W), bulb (B), rest of the strip (R), and all identified proteins (W + B + R); Table S6: Molecular functions of identified proteins in the whole strip (W), bulb (B), rest of the strip (R), and all identified proteins; Table S7: List of proteins identified in the 6 chosen signaling pathways. Figure S1: Venn diagram comparing other four comprehensive proteomics studies; Figure S2: Functional analysis and structural classification of unique proteins in each batch. (**a**) Subgroups of biological processes. (**b**) Subgroups of molecular function. (**c**) The list and number of protein classes in each ScS section.
