# Supplementary material for: Proteomic Analysis of Tears and Conjunctival Cells Collected with Schirmer Strips Using timsTOF Pro: Preanalytical Considerations
Source: Metabolites. 2021 Dec 21;12(1):2. doi: 10.3390/metabo12010002 (PMC8778087; doi:10.3390/metabo12010002)
Supplement: Supplementary file 1 [file metabolites-12-00002-s001.zip › metabolites-1479124-supplementary-for-pub.pdf]

Supplementary tables

Table S1. Raw data of all identified proteins provided by the Schirmer strip extract of the 3 batches (W+B+R).

					Number of peptides									Specific unique peptides									MS/MS count								
				Sample	1 [B]			2 [R]			3 [W]			1 [B]			2 [R]			3 [W]			1 [B]			2 [R]			3 [W]		
Protein names	Gene names	Number of protein isoforms	Number of Peptides	Specific Unique peptides	B-1	B-2	B-3	R-1	R-2	R-3	W-1	W-2	W-3	B-1	B-2	B-3	R-1	R-2	R-3	W-1	W-2	W-3	B-1	B-2	B-3	R-1	R-2	R-3	W-1	W-2	W-3
Prolactin-inducible protein	PIP	1	16	16	4	14	13	14	15	15	14	15	15	12	14	13	14	15	15	14	15	15	95	104	125	122	119	124	129	141	157
Endorepellin	HSPG2	1	161	161	54	49	48	103	98	104	94	80	92	54	49	48	103	98	104	94	80	92	62	54	53	124	125	122	119	108	113
Lysozyme C	LYZ	1	29	29	23	22	21	23	23	21	22	19	21	23	22	21	23	23	21	22	19	21	109	100	103	109	103	114	104	113	117
Ig alpha-1 chain C region	IGHA1	1	26	17	20	17	19	22	21	20	20	20	21	15	14	14	16	15	14	14	14	15	58	64	63	77	69	76	110	105	106
Serum albumin	ALB	2	95	88	76	70	73	46	44	45	51	50	50	70	65	67	42	40	41	47	46	46	209	195	209	71	63	67	101	104	100
Lactotransferrin	LTF	1	85	83	68	68	70	67	67	68	63	61	60	66	66	68	66	66	67	61	59	58	138	124	129	98	105	102	107	101	94
Polymeric immunoglobulin receptor	PIGR	1	55	55	4	32	31	39	41	39	39	39	40	34	32	31	39	41	39	39	39	40	71	55	54	87	79	84	104	99	93
Cystatin-S	CST4	2	17	14	13	14	14	13	14	14	16	15	16	10	11	11	10	11	11	13	12	13	43	39	46	78	71	81	90	87	96
Complement C3	C3	1	114	107	57	57	56	54	55	54	58	58	58	54	54	52	51	52	50	54	55	52	71	68	70	62	65	61	74	69	75
Proline-rich protein 4	PRR4	1	8	8	4	6	6	6	6	6	7	7	7	6	6	6	6	6	6	7	7	7	26	23	32	42	48	54	72	71	71
Ceruloplasmin	CP	2	53	53	36	33	34	39	39	37	36	36	37	36	33	34	39	39	37	36	36	37	44	45	41	51	52	48	53	53	55
Deleted in malignant brain tumors 1 protein	DMBT1	1	39	39	29	29	29	31	31	31	31	33	28	29	29	29	31	31	31	31	33	28	50	48	46	54	55	54	53	53	46
Extracellular glycoprotein lacritin	LACRT	1	12	12	6	9	7	11	11	11	10	11	9	6	9	7	11	11	11	10	11	9	14	22	16	31	31	26	43	48	58
Lipocalin-1	LCN1	2	27	27	24	21	24	19	20	19	22	18	19	24	21	24	19	20	19	22	18	19	54	49	47	38	38	34	47	46	43
Mammaglobin-B	SCGB2A1	1	12	12	4	7	6	3	4	4	7	7	7	7	7	6	3	4	4	7	7	7	17	19	18	5	7	7	35	35	36
Retinal dehydrogenase 1	ALDH1A1	1	40	37	33	28	29	22	21	20	26	27	25	31	27	27	21	20	18	25	26	24	50	42	46	27	24	23	37	36	33
Ig kappa chain C region	IGKC	1	9	9	8	8	9	7	8	7	8	8	8	8	8	9	7	8	7	8	8	8	17	21	16	13	18	16	30	33	34
Alpha-enolase	ENO1	1	41	38	33	28	34	23	22	23	24	23	22	30	25	31	21	19	20	22	21	20	56	47	53	27	27	28	32	30	32
Immunoglobulin J chain	IGJ	1	18	18	13	13	11	15	13	14	13	12	13	13	13	11	15	13	14	13	12	13	19	22	22	29	28	26	27	33	32
Alpha-actinin-4	ACTN4	4	56	40	10	12	9	27	28	24	27	23	27	6	7	6	17	17	17	20	17	19	11	13	9	28	30	26	32	28	32
Clusterin	CLU	1	28	28	14	15	16	13	16	13	16	17	18	14	15	16	13	16	13	16	17	18	17	18	19	20	23	19	28	26	30
Heat shock 70 kDa protein 1B	HSPA1B/A	2	36	25	31	26	30	22	22	25	23	24	25	21	17	20	15	14	17	15	15	17	39	33	35	27	26	29	26	29	29
Myosin-9	MYH9	9	80	69	13	14	11	39	37	39	28	28	28	11	11	8	32	32	33	23	22	23	12	13	11	39	39	40	27	28	28
Pyruvate kinase PKM	PKM	2	41	41	29	31	31	14	15	16	23	26	20	29	31	31	14	15	16	23	26	20	43	41	41	16	16	18	28	31	23
Actin, cytoplasmic 2	ACTG1	1	31	1	17	15	17	16	15	14	16	18	17	1	1	1	1	1	1	1	1	1	26	25	26	26	24	22	23	30	29
Nucleobindin-2	NUCB2	1	26	26	4	12	14	13	14	14	17	15	16	15	12	14	13	14	14	17	15	16	25	18	20	18	23	21	29	24	27
Myosin-14	MYH14	1	91	84	4	30	29	8	10	8	26	25	23	28	27	26	4	7	5	22	21	20	33	33	30	9	11	8	27	28	24
Nucleobindin-1	NUCB1	1	32	32	4	15	17	23	20	18	23	23	22	15	15	17	23	20	18	23	23	22	18	17	21	27	23	22	26	25	28
Mesothelin	MSLN	1	26	26	17	18	14	20	21	23	21	20	20	17	18	14	20	21	23	21	20	20	20	22	17	23	27	26	24	27	25
Keratin, type II cytoskeletal 1	KRT1	1	50	4	20	17	20	40	41	43	23	20	22	0	0	0	3	4	3	0	1	0	22	18	22	58	56	64	26	25	25
Serotransferrin	TF	1	57	56	4	47	42	24	28	27	24	22	26	44	46	41	24	28	27	24	22	26	63	59	55	25	28	27	24	22	28
Phosphoglycerate kinase 1	PGK1	1	27	24	4	26	21	15	11	16	17	18	18	20	23	18	13	9	14	15	16	16	36	38	33	22	18	22	25	24	24
Lactoperoxidase	LPO	1	42	42	15	15	16	25	24	21	21	21	22	15	15	16	25	24	21	21	21	22	19	18	17	32	30	26	24	24	24
Ig mu chain C region	IGHM	1	27	27	14	11	12	15	13	11	19	19	16	14	11	12	15	13	11	19	19	16	18	14	16	18	14	12	23	26	18
Annexin A1	ANXA1	1	31	31	19	21	21	16	17	16	17	19	18	19	21	21	16	17	16	17	19	18	24	25	25	17	19	18	22	23	22
Heat shock protein HSP 90-alpha	HSP90AA1	4	43	29	10	5	9	25	21	24	21	24	19	8	4	7	15	11	14	14	16	14	10	5	9	26	23	24	22	24	21
Gelsolin	GSN	1	34	16	20	20	21	16	15	13	18	19	17	10	12	12	9	8	5	10	12	10	27	22	28	19	19	18	23	23	20
Glycogen phosphorylase, liver form	PYGL	1	41	33	4	20	23	3	3	2	18	19	20	18	17	19	2	2	2	14	16	16	22	22	26	3	3	2	21	19	23
Ig alpha-2 chain C region	IGHA2	1	16	7	11	9	10	10	10	11	12	11	11	6	6	5	4	4	5	6	5	5	14	14	12	15	12	14	23	18	22
SPARC-like protein 1	SPARCL1	1	22	22	4	14	12	16	18	16	17	18	17	13	14	12	16	18	16	17	18	17	15	17	14	19	22	20	19	21	22
Ezrin	EZR	1	41	31	27	22	21	17	13	13	18	18	16	20	16	17	11	9	10	14	13	12	34	28	28	18	15	13	22	20	19
Heat shock cognate 71 kDa protein	HSPA8	2	37	33	27	26	27	20	21	19	17	16	16	24	23	23	17	18	16	14	13	13	36	33	36	25	26	24	22	20	17

Cathepsin D	CTSD	1	20	20	11	12	9	15	13	14	16	15	15	11	12	9	15	13	14	16	15	15	17	18	14	20	19	19	19	18	20
Protein S100-A9	S100A9	1	14	14	4	9	10	10	9	8	10	9	9	10	9	10	10	9	8	10	9	9	31	27	28	21	22	23	19	18	20
Protein-glutamine gamma-glutamyltransferase 2	TGM2	1	36	36	4	27	25	11	9	7	17	15	17	25	27	25	11	9	7	17	15	17	33	34	36	13	9	8	20	16	19
Cathepsin B	CTSB	1	20	20	14	13	14	14	16	17	12	13	12	14	13	14	14	16	17	12	13	12	21	18	20	20	23	23	20	17	18
Aldehyde dehydrogenase family 1 member A3	ALDH1A3	2	27	26	18	18	20	5	8	8	16	17	14	17	18	19	4	8	7	16	17	14	24	22	25	4	8	7	19	20	15
Haptoglobin	HP	1	24	12	15	17	14	13	12	13	12	14	14	8	8	7	7	6	8	7	7	7	18	21	18	14	14	14	16	18	20
Ubiquitin-like modifier-activating enzyme 1	UBA1	1	38	38	4	7	5	9	10	7	16	16	15	7	7	5	9	10	7	16	16	15	8	8	6	10	11	8	18	17	17
Complement factor B	CFB	2	33	33	17	18	16	13	15	12	15	15	15	17	18	16	13	15	12	15	15	15	19	23	20	14	16	13	17	18	17
Elongation factor 2	EEF2	1	37	37	10	11	7	22	21	18	14	19	16	10	11	7	22	21	18	14	19	16	10	11	7	23	21	18	14	20	18
Galectin-3-binding protein	LGALS3BP	1	24	24	10	13	10	16	17	18	15	15	14	10	13	10	16	17	18	15	15	14	13	16	12	21	20	22	18	18	16
Glutathione S-transferase P	GSTP1	1	16	16	11	11	10	8	7	7	10	10	10	11	11	10	8	7	7	10	10	10	20	20	20	11	9	10	18	17	16
Submaxillary gland androgen-regulated protein 3B	SMR3B	1	2	2	4	2	2	2	2	2	2	2	2	2	2	2	2	2	2	2	2	2	26	20	18	18	16	16	15	17	19
Cystatin-SN	CST1	1	13	10	10	10	10	10	11	10	11	11	11	7	7	7	7	8	7	8	8	8	10	9	9	9	10	9	19	16	15
Heat shock protein beta-1	HSPB1	1	16	16	14	12	12	9	7	8	11	12	10	14	12	12	9	7	8	11	12	10	22	17	18	10	9	10	17	16	17
Peroxiredoxin-1	PRDX1	1	22	19	4	18	17	10	11	9	11	11	14	14	16	15	8	9	7	9	10	12	21	24	24	13	14	12	16	15	19
Glyceraldehyde-3-phosphate dehydrogenase	GAPDH	1	22	22	17	14	15	13	11	11	13	12	12	17	14	15	13	11	11	13	12	12	35	27	29	15	13	13	18	17	15
Calumenin	CALU	1	19	19	9	10	10	13	12	13	13	11	12	9	10	10	13	12	13	13	11	12	12	12	12	17	16	19	19	15	16
Fructose-bisphosphate aldolase A	ALDOA	1	26	23	23	22	23	13	14	11	16	14	15	20	19	20	11	12	8	14	11	12	33	36	38	15	14	12	17	15	17
Proline-rich protein 1	PROL1	1	8	8	4	6	6	7	7	6	7	7	7	6	6	6	7	7	6	7	7	7	12	15	11	13	17	11	17	15	17
Tubulin beta-4B chain	TUBB4B	1	26	1	4	6	5	10	9	12	16	13	17	1	1	0	0	0	1	1	1	1	8	6	6	11	9	12	17	14	17
Transcobalamin-1	TCN1	1	14	14	4	9	9	10	13	13	9	8	10	9	9	9	10	13	13	9	8	10	15	15	13	16	20	19	16	14	18
Leukocyte elastase inhibitor	SERPINB1	2	26	25	19	19	19	5	10	9	12	15	12	18	18	18	4	9	8	11	14	11	23	25	25	5	11	10	14	18	15
Tripeptidyl-peptidase 1	TPP1	1	14	14	4	6	8	11	11	10	11	12	9	6	6	8	11	11	10	11	12	9	9	9	11	15	15	14	16	17	14
Protein S100-A8	S100A8	1	17	17	4	12	11	13	13	11	10	10	9	13	12	11	13	13	11	10	10	9	26	21	20	25	20	21	16	16	15
DnaJ homolog subfamily C member 3	DNAJC3	1	21	21	1	1	0	10	12	9	14	14	13	1	1	0	10	12	9	14	14	13	1	1	0	10	13	10	16	16	14
6-phosphogluconate dehydrogenase, decarboxylating	PGD	1	22	22	4	14	16	5	6	7	14	11	14	20	14	16	5	6	7	14	11	14	23	16	16	6	7	7	15	14	16
Alpha-amylase 1	AMY1A/B/2A	4	17	17	6	5	9	10	8	9	13	13	12	6	5	9	10	8	9	13	13	12	6	6	10	11	10	10	15	16	14
Golgi membrane protein 1	GOLM1	1	21	21	10	13	13	13	13	13	12	12	13	10	13	13	13	13	13	12	12	13	11	14	14	15	15	14	15	13	17
Fructose-1,6-bisphosphatase 1	FBP1	1	22	21	18	17	17	7	7	6	12	11	13	17	16	16	7	7	6	11	10	12	26	24	25	7	7	6	15	14	15
Putative elongation factor 1-alpha-like 3	EEF1A1P5;EEF1A1	2	17	9	8	9	9	9	9	9	9	8	10	6	7	6	7	6	7	7	6	7	10	12	11	13	13	15	15	13	16
Selenium-binding protein 1	SELENBP1	1	37	37	4	25	27	7	8	7	16	12	14	26	25	27	7	8	7	16	12	14	32	31	34	7	8	7	16	12	15
Chloride intracellular channel protein 1	CLIC1	1	20	20	15	14	15	9	8	6	12	12	13	15	14	15	9	8	6	12	12	13	19	18	18	9	8	6	14	13	16
Phosphatidylethanolamine-binding protein 1	PEBP1	1	15	15	4	12	11	9	8	7	10	10	10	12	12	11	9	8	7	10	10	10	20	19	20	10	9	8	12	15	15
Triosephosphate isomerase	TPI1	1	23	23	4	17	17	14	15	13	12	11	11	18	17	17	14	15	13	12	11	11	22	22	23	16	17	13	15	14	13
Prosaposin	PSAP	1	20	19	4	12	12	15	14	14	12	14	12	12	12	12	15	14	14	12	14	12	14	13	13	17	17	17	14	15	13
Serpin B5	SERPINB5	1	24	24	4	9	11	13	15	13	14	12	12	10	9	11	13	15	13	14	12	12	10	10	11	14	15	14	15	13	13
Ribonuclease inhibitor	RNH1	1	29	29	4	14	16	13	15	16	13	13	13	17	14	16	13	15	16	13	13	13	19	14	19	13	15	16	14	13	14
Complement factor H	CFH	2	45	41	24	19	18	14	14	16	13	13	15	22	17	16	14	14	15	13	13	15	24	19	18	14	14	17	13	13	15
Protein disulfide-isomerase	P4HB	1	30	30	4	8	10	25	22	23	12	12	12	10	8	10	25	22	23	12	12	12	13	8	11	28	25	26	14	14	13
Ras GTPase-activating-like protein IQGAP1	IQGAP1	1	50	49	2	1	1	14	13	13	15	13	12	2	1	1	14	13	13	15	13	12	2	1	1	15	13	13	15	13	12
Rab GDP dissociation inhibitor beta	GDI2	1	25	17	9	8	8	6	6	6	11	10	11	6	5	6	5	5	6	8	7	8	9	8	8	6	6	6	14	12	13
Neutrophil gelatinase-associated lipocalin	LCN2	1	11	11	7	7	8	10	10	10	9	9	9	7	7	8	10	10	10	9	9	9	8	7	9	11	11	11	13	12	14
14-3-3 protein zeta/delta	YWHAZ	1	22	18	4	14	12	13	13	13	12	11	12	12	13	11	11	12	12	11	9	10	14	15	13	12	12	13	14	12	13
Procollagen-lysine,2-oxoglutarate 5-dioxygenase 1	PLOD1	1	26	26	4	7	9	16	13	13	11	11	12	7	7	9	16	13	13	11	11	12	7	7	9	18	15	15	12	13	14
Cytosol aminopeptidase	LAP3	1	31	31	21	21	21	3	4	2	11	12	12	21	21	21	3	4	2	11	12	12	30	32	29	3	4	2	12	13	13
Keratin, type I cytoskeletal 19	KRT19	1	40	1	20	13	16	5	5	5	11	12	12	0	0	0	0	0	0	0	0	0	21	13	16	5	5	4	11	14	13
Transgelin-2	TAGLN2	1	22	21	4	16	18	7	8	7	15	11	11	15	15	17	7	8	7	14	10	11	19	19	22	7	9	7	16	11	11
Hemopexin	HPX	1	22	22	15	15	15	8	6	7	12	11	11	15	15	15	8	6	7	12	11	11	19	20	18	8	7	9	13	12	13
Ig lambda-6 chain C region	IGLC6	4	10	3	4	6	6	7	7	5	4	4	4	1	2	2	2	2	2	1	2	2	7	14	13	13	15	9	15	14	9
WD repeat-containing protein 1	WDR1	1	24	24	4	20	20	12	11	13	13	10	13	20	20	20	12	11	13	13	10	13	25	29	25	12	13	15	13	11	14

Zinc-alpha-2-glycoprotein	AZGP1	1	20	20	11	10	12	13	13	15	10	11	9	11	10	12	13	13	15	10	11	9	16	13	14	15	16	18	13	14	11
Annexin A2	ANXA2;ANXA2P2	2	33	33	26	29	27	20	23	21	11	11	14	26	29	27	20	23	21	11	11	14	33	40	37	24	26	25	12	12	14
Aldehyde dehydrogenase, dimeric NADP-preferring	ALDH3A1	2	27	26	20	17	17	7	8	6	10	11	11	20	16	16	7	8	6	10	10	11	26	23	22	7	8	6	12	13	12
Filamin-B	FLNB	1	55	53	23	16	22	19	17	16	11	14	12	21	14	20	18	16	15	10	13	11	23	16	22	21	18	17	11	14	12
78 kDa glucose-regulated protein	HSPA5	1	33	32	17	14	16	28	27	25	14	13	11	16	14	15	27	26	25	14	13	10	18	17	18	35	31	33	14	13	10
Argininosuccinate synthase	ASS1	1	29	29	17	16	16	4	2	3	9	11	10	17	16	16	4	2	3	9	11	10	20	18	20	4	2	3	12	12	12
Alcohol dehydrogenase class 4 mu/sigma chain	ADH7	1	27	27	12	14	13	4	3	3	12	10	10	12	14	13	4	3	3	12	10	10	13	18	16	4	3	3	13	10	13
45 kDa calcium-binding protein	SDF4	1	17	17	4	6	7	9	8	9	12	10	11	4	6	7	9	8	9	12	10	11	5	7	8	9	9	9	13	11	12
Sulfhydryl oxidase 1	QSOX1	1	18	18	4	1	1	10	10	11	10	12	11	1	1	1	10	10	11	10	12	11	1	1	1	10	10	11	11	13	12
Tubulin alpha-1B chain	TUBA1B	3	20	0	7	7	6	12	13	11	11	9	11	0	0	0	0	0	0	0	0	0	7	7	6	14	15	14	12	11	13
Annexin A3	ANXA3	1	22	22	15	19	16	4	2	5	10	10	11	15	19	16	4	2	5	10	10	11	17	22	18	4	2	5	11	12	12
Peroxiredoxin-6	PRDX6	1	19	19	4	6	7	5	7	5	10	10	9	7	6	7	5	7	5	10	10	9	7	6	7	6	8	6	12	12	11
Plastin-3	PLS3	1	22	16	4	0	0	8	6	9	11	11	10	1	0	0	4	3	5	6	7	6	1	0	0	8	6	9	12	12	11
Neutral alpha-glucosidase AB	GANAB	1	24	24	4	4	5	8	7	6	10	10	12	4	4	5	8	7	6	10	10	12	5	5	6	9	7	8	11	11	13
Annexin A5	ANXA5	1	24	24	19	17	20	6	4	6	11	11	10	19	17	20	6	4	6	11	11	10	20	18	21	6	4	6	11	11	10
Apolipoprotein A-I	APOA1	1	32	32	14	10	10	7	6	4	11	11	10	14	10	10	7	6	4	11	11	10	14	10	10	7	6	4	11	11	10
Cystatin-C	CST3	1	8	8	6	6	5	6	7	6	7	7	7	6	6	5	6	7	6	7	7	7	9	10	8	9	10	7	11	11	10
Adenylyl cyclase-associated protein 1	CAP1	2	26	26	4	6	6	7	10	8	12	10	9	4	6	6	7	10	8	12	10	9	4	6	6	8	10	8	12	10	10
Soluble calcium-activated nucleotidase 1	CANT1	1	13	13	3	3	5	9	10	9	10	10	11	3	3	5	9	10	9	10	10	11	3	3	5	9	10	9	10	11	11
Transitional endoplasmic reticulum ATPase	VCP	1	41	41	4	8	8	18	13	16	9	13	8	11	8	8	18	13	16	9	13	8	11	9	8	19	13	16	10	14	8
Fatty acid-binding protein, epidermal	FABP5	2	17	17	10	11	11	13	12	14	8	9	8	10	11	11	13	12	14	8	9	8	15	17	16	21	19	21	10	13	9
Mucin-5AC	MUC5AC	1	72	68	4	44	51	0	0	0	11	10	9	45	41	49	0	0	0	11	10	9	56	50	57	0	0	0	11	10	10
Ribonuclease T2	RNASET2	1	10	10	4	3	5	6	7	7	6	6	7	7	3	5	6	7	7	6	6	7	8	3	6	8	12	9	10	10	11
Alcohol dehydrogenase [NADP(+)]	AKR1A1	1	18	18	16	14	15	10	8	7	10	8	9	16	14	15	10	8	7	10	8	9	18	17	15	10	8	8	11	9	10
Calpain-1 catalytic subunit	CAPN1	1	24	24	1	1	1	7	4	5	10	10	9	1	1	1	7	4	5	10	10	9	1	1	1	7	4	5	10	10	9
Interleukin-1 receptor antagonist protein	IL1RN	1	10	10	9	8	9	5	4	4	5	7	7	9	8	9	5	4	4	5	7	7	14	11	12	8	7	6	8	10	11
Heat shock protein HSP 90-beta	HSP90AB1	2	37	21	5	3	4	18	23	19	18	15	15	2	2	2	6	11	7	9	5	8	3	2	2	11	14	10	11	7	11
L-lactate dehydrogenase A chain	LDHA	3	30	28	20	23	21	12	13	13	9	10	8	18	21	19	11	12	12	8	9	7	24	29	27	13	13	13	10	11	8
Neuroblast differentiation-associated protein AHNAK	AHNAK	1	114	114	28	30	31	47	38	42	10	7	12	28	30	31	47	38	42	10	7	12	30	30	32	51	41	46	10	7	12
Glutathione reductase, mitochondrial	GSR	1	19	19	12	11	12	0	1	2	8	9	9	12	11	12	0	1	2	8	9	9	14	16	15	0	1	2	9	10	9
Peroxiredoxin-5, mitochondrial	PRDX5	1	15	15	4	12	12	9	10	6	8	8	9	13	12	12	9	10	6	8	8	9	18	16	16	9	11	6	10	8	10
Histone H1.4	HIST1H1E	3	10	3	7	3	6	6	7	7	5	4	5	3	2	2	2	3	2	2	2	3	9	6	9	9	10	10	10	9	9
Granulins	GRN	1	17	17	11	9	7	10	10	9	7	9	7	11	9	7	10	10	9	7	9	7	13	11	7	10	12	11	9	10	9
Chitinase-3-like protein 2	CHI3L2	1	13	13	4	5	4	11	10	9	9	12	7	4	5	4	11	10	9	9	12	7	4	5	4	13	11	10	9	12	7
Protein disulfide-isomerase A3	PDIA3	1	31	31	4	20	17	15	17	15	9	9	10	20	20	17	15	17	15	9	9	10	20	21	18	15	17	15	9	9	10
UMP-CMP kinase	CMPK1	1	12	12	8	9	10	3	4	4	6	7	6	8	9	10	3	4	4	6	7	6	12	12	14	3	4	4	9	10	8
Thymosin beta-4	TMSB4X	1	7	7	4	5	6	4	4	3	4	3	4	6	5	6	4	4	3	4	3	4	17	16	16	6	5	4	9	7	11
Programmed cell death 6-interacting protein	PDCD6IP	1	29	29	4	2	3	4	4	7	9	9	9	3	2	3	4	4	7	9	9	9	3	2	3	4	4	7	9	9	9
Peptidyl-prolyl cis-trans isomerase A	PPIA	7	12	12	11	10	12	8	6	6	8	7	9	11	10	12	8	6	6	8	7	9	21	20	17	10	7	8	9	8	10
Isocitrate dehydrogenase [NADP] cytoplasmic	IDH1	1	16	16	10	8	9	4	1	4	7	7	7	10	8	9	4	1	4	7	7	7	12	9	11	4	1	4	8	9	9
Monocyte differentiation antigen CD14	CD14	1	11	11	1	1	1	5	5	3	7	7	7	1	1	1	5	5	3	7	7	7	1	1	1	5	6	3	8	9	9
Cofilin-1	CFL1	1	16	13	11	10	13	7	7	9	11	5	6	8	8	11	5	5	6	8	3	3	16	13	17	8	7	11	13	6	7
Nicotinate phosphoribosyltransferase	NAPRT	1	28	28	4	18	15	2	3	1	9	8	8	15	18	15	2	3	1	9	8	8	16	21	17	2	3	1	9	8	8
Alpha-2-HS-glycoprotein	AHSG	2	11	11	7	7	7	3	4	3	7	6	6	7	7	7	3	4	3	7	6	6	8	8	11	4	4	4	9	8	8
Thioredoxin	TXN	1	9	9	4	6	6	4	6	5	4	4	4	7	6	6	4	6	5	4	4	4	11	8	10	4	7	5	9	8	8
Hypoxia up-regulated protein 1	HYOU1	1	14	14	1	1	1	6	7	6	8	8	9	1	1	1	6	7	6	8	8	9	1	1	1	6	7	6	8	8	9
Phosphatidylethanolamine-binding protein 4	PEBP4	1	8	8	4	6	5	7	5	6	7	7	7	6	6	5	7	5	6	7	7	7	8	7	5	10	6	7	9	8	8
Secreted frizzled-related protein 1	SFRP1	1	10	10	4	6	5	5	8	6	5	7	6	3	6	5	5	8	6	5	7	6	4	9	7	8	10	7	8	8	9
Echinoderm microtubule-associated protein-like 2	EML2	4	20	20	15	16	13	1	2	1	5	8	6	15	16	13	1	2	1	5	8	6	20	21	16	1	2	1	8	9	7
Antithrombin-III	SERPINC1	2	21	21	12	14	11	1	1	2	8	7	6	12	14	11	1	1	2	8	7	6	16	17	16	1	1	2	9	9	6

14-3-3 protein epsilon	YWHAE	1	20	18	4	7	8	5	5	6	8	7	8	8	6	7	3	4	5	7	6	7	9	6	8	3	4	6	9	7	8
Metalloproteinase inhibitor 1	TIMP1	1	8	8	4	6	7	6	7	7	6	6	5	7	6	7	6	7	7	6	6	5	10	9	10	8	10	11	9	8	7
Protein S100-P	S100P	1	11	11	4	8	9	0	0	1	5	7	5	6	8	9	0	0	1	5	7	5	8	11	12	0	0	1	7	9	7
Glutamine-fructose-6-phosphate aminotransferase 1	GFPT1	2	20	20	1	0	0	1	2	1	8	9	6	1	0	0	1	2	1	8	9	6	1	0	0	1	2	1	8	9	6
Galectin-3	LGALS3	1	10	10	7	7	7	5	4	5	5	4	5	7	7	7	5	4	5	5	4	5	12	12	12	7	6	7	8	7	8
Phosphoglycerate mutase 1	PGAM1	3	17	17	10	10	12	7	7	7	7	6	7	10	10	12	7	7	7	7	6	7	11	12	13	8	8	8	9	6	8
Beta-hexosaminidase subunit beta	HEXB	1	14	14	4	4	5	5	7	8	7	7	7	4	4	5	5	7	8	7	7	7	5	5	5	6	8	10	7	9	7
Rho GDP-dissociation inhibitor 2	ARHGDIB	1	16	16	10	7	9	2	1	2	7	8	7	10	7	9	2	1	2	7	8	7	10	7	9	2	1	2	7	8	7
Phospholipid transfer protein	PLTP	1	16	16	4	2	2	3	3	3	9	6	5	2	2	2	3	3	3	9	6	5	2	2	2	3	3	3	9	7	6
PDZ and LIM domain protein 1	PDLIM1	1	18	18	4	12	15	4	4	3	8	7	7	14	12	15	4	4	3	8	7	7	16	13	17	4	4	3	8	7	7
Vitamin D-binding protein	GC	3	26	26	17	17	17	6	7	6	9	8	5	17	17	17	6	7	6	9	8	5	20	20	23	6	7	6	9	8	5
Myosin light polypeptide 6	MYL6	2	12	11	7	7	7	5	6	6	5	6	6	7	7	7	5	6	6	5	6	6	10	11	9	7	8	7	6	8	8
Tropomyosin alpha-4 chain	TPM4	3	19	14	17	12	14	7	8	7	5	7	7	13	10	10	4	5	4	3	4	5	20	13	15	8	9	7	6	8	8
Calmodulin-3	CALM-3	3	10	10	7	7	7	7	5	6	4	4	5	7	7	7	7	5	6	4	4	5	10	11	11	10	8	9	8	7	7
Lipolysis-stimulated lipoprotein receptor	LSR	1	14	14	4	3	4	8	8	11	9	6	7	4	3	4	8	8	11	9	6	7	4	3	4	8	8	11	9	6	7
Ig gamma-1 chain C region	IGHG1	1	10	5	9	9	8	9	10	10	7	6	8	5	5	4	5	5	5	3	4	5	11	11	9	12	11	12	7	8	7
Calmodulin-like protein 5	CALML5	1	14	14	9	8	9	11	12	12	7	7	7	9	8	9	11	12	12	7	7	7	11	10	12	16	20	19	8	7	7
Alcohol dehydrogenase 1C	ADH1C/A	3	15	8	10	7	10	0	0	0	5	7	8	6	5	6	0	0	0	4	5	6	13	10	13	0	0	0	6	7	8
Actin-related protein 2/3 complex subunit 1B	ARPC1B	1	14	13	6	5	8	3	2	1	7	7	7	5	4	7	3	2	1	6	6	6	6	5	8	3	2	1	7	7	7
Proteasome activator complex subunit 1	PSME1	1	10	10	4	1	1	2	3	3	6	7	6	1	1	1	2	3	3	6	7	6	1	1	1	2	3	3	7	8	6
Cytosolic non-specific dipeptidase	CNDP2	1	26	26	11	11	9	2	4	3	7	8	6	11	11	9	2	4	3	7	8	6	12	12	10	2	4	3	7	8	6
Perilipin-3	PLIN3	1	19	19	4	5	5	6	4	4	7	8	6	7	5	5	6	4	4	7	8	6	7	5	5	6	4	4	7	8	6
F-actin-capping protein subunit alpha-1	CAPZA1	1	13	12	4	7	8	6	4	5	6	7	5	4	6	7	6	4	5	6	7	5	5	7	9	6	4	5	7	8	6
Ig-like domain-containing protein	IGHV3OR16-9	1	4	1	2	2	3	2	2	2	2	2	2	1	1	1	1	1	1	1	1	1	5	4	3	5	7	4	8	7	6
Profilin-1	PFN1	2	13	13	11	7	7	6	6	7	6	5	6	11	7	7	6	6	7	6	5	6	16	10	11	6	7	7	7	6	8
Serine protease HTRA1	HTRA1	1	14	14	0	0	1	7	8	5	8	7	6	0	0	1	7	8	5	8	7	6	0	0	1	7	8	5	8	7	6
Clathrin heavy chain 1	CLTC	2	43	43	0	1	0	8	8	8	7	7	6	0	1	0	8	8	8	7	7	6	0	1	0	9	8	8	7	8	6
Fibrinogen beta chain	FGB	2	25	25	5	3	5	15	18	14	7	6	8	5	3	5	15	18	14	7	6	8	5	3	5	16	19	15	7	6	8
Anterior gradient protein 2 homolog	AGR2	1	12	11	10	10	9	0	0	0	6	5	6	9	9	8	0	0	0	5	4	5	16	14	13	0	0	0	7	6	7
Dipeptidyl peptidase 3	DPP3	1	20	20	13	14	14	4	4	3	7	7	5	13	14	14	4	4	3	7	7	5	16	14	15	5	4	3	8	7	5
Puromycin-sensitive aminopeptidase	NPEPPS	2	22	22	11	13	13	6	6	8	7	6	7	11	13	13	6	6	8	7	6	7	12	13	14	6	6	8	7	6	7
14-3-3 protein sigma	SFN	1	23	21	4	9	11	16	15	14	7	6	6	14	8	10	14	14	13	6	5	5	16	10	12	17	19	18	8	6	6
Bifunctional purine biosynthesis protein PURH	ATIC	1	32	32	28	21	17	0	1	1	7	4	7	28	21	17	0	1	1	7	4	7	35	26	21	0	1	1	7	4	8
Aldo-keto reductase family 1 member C1	AKR1C1	1	23	2	18	14	15	2	2	2	5	6	5	2	2	1	0	0	0	1	1	1	26	18	21	2	2	2	6	7	6
Sorbitol dehydrogenase	SORD	1	18	18	4	9	9	3	2	1	6	7	6	11	9	9	3	2	1	6	7	6	11	9	9	3	2	1	6	7	6
Leukotriene A-4 hydrolase	LTA4H	1	25	25	19	17	17	2	2	3	4	7	4	19	17	17	2	2	3	4	7	4	22	19	20	2	2	3	5	9	5
Alpha-1-antitrypsin	SERPINA1	1	23	23	4	10	12	3	4	4	6	5	6	12	10	12	3	4	4	6	5	6	13	12	13	3	4	4	6	6	7
Proteasome subunit alpha type-7	PSMA7	2	13	13	9	9	10	5	5	5	5	6	7	9	9	10	5	5	5	5	6	7	10	10	13	5	5	5	5	6	8
Macrophage-capping protein	CAPG	1	15	15	3	4	2	5	5	5	5	7	7	3	4	2	5	5	5	5	7	7	3	4	2	5	5	5	5	7	7
Glucose-6-phosphate isomerase	GPI	1	23	17	16	16	17	7	5	6	7	6	5	12	12	13	4	4	4	5	4	4	23	20	18	7	5	6	7	7	5
Reticulocalbin-1	RCN1	1	12	12	4	5	4	5	4	5	5	5	5	4	5	4	5	4	5	5	5	5	5	8	6	7	6	6	6	6	7
Retinoic acid receptor responder protein 1	RARRES1	1	8	8	4	1	0	7	6	7	6	5	6	1	1	0	7	6	7	6	5	6	1	1	0	8	6	8	7	6	6
Glutathione peroxidase 1	GPX1	1	13	13	6	8	7	0	0	0	6	4	6	6	8	7	0	0	0	6	4	6	7	10	10	0	0	0	7	4	7
GDP-mannose 4,6 dehydratase	GMDS	1	13	13	6	8	6	1	0	1	6	5	7	6	8	6	1	0	1	6	5	7	6	9	6	1	0	1	6	5	7
Plasma protease C1 inhibitor	SERPING1	1	13	13	4	8	11	1	2	2	5	5	8	8	8	11	1	2	2	5	5	8	9	9	11	1	2	2	5	5	8
Prostasin	PRSS8	1	5	5	4	2	1	2	2	2	5	4	4	1	2	1	2	2	2	5	4	4	1	3	2	3	3	4	7	6	5
Transketolase	TKT	1	27	27	4	13	14	4	3	3	7	6	4	19	13	14	4	3	3	7	6	4	22	16	16	5	4	4	8	6	4
Involucrin	IVL	1	19	19	4	4	1	17	16	16	5	7	6	4	4	1	17	16	16	5	7	6	4	4	1	23	19	19	5	7	6
Protein S100-A6	S100A6	1	13	13	4	8	7	2	1	3	6	6	4	5	8	7	2	1	3	6	6	4	6	9	9	2	1	3	6	7	4
Proteasome activator complex subunit 2	PSME2	1	12	12	4	1	0	4	2	4	4	6	5	0	1	0	4	2	4	4	6	5	0	1	0	4	2	4	5	6	6

F-actin-capping protein subunit beta	CAPZB	1	15	14	5	4	4	4	3	4	7	5	5	5	4	4	4	3	4	7	5	5	6	4	4	4	3	4	7	5	5
Drebrin-like protein	DBNL	1	11	11	9	9	7	4	4	4	4	5	6	9	9	7	4	4	4	4	5	6	10	9	8	4	4	4	5	6	6
Na(+)/H(+) exchange regulatory cofactor NHE-RF1	SLC9A3R1	1	15	15	4	10	12	4	5	3	7	5	5	11	10	12	4	5	3	7	5	5	12	11	13	4	6	3	7	5	5
Antileukoproteinase	SLPI	1	7	7	4	3	4	2	4	4	4	3	3	5	3	4	2	4	4	4	3	3	5	3	5	2	5	6	7	4	6
Heat shock 70 kDa protein 4	HSPA4	1	21	20	13	11	9	5	5	4	6	5	6	12	10	8	5	5	4	6	5	5	13	11	9	5	5	4	6	5	6
Beta-2-microglobulin	B2M	1	8	8	4	3	4	5	6	5	4	5	4	4	3	4	5	6	5	4	5	4	5	3	6	5	6	6	5	6	6
Glutamine synthetase	GLUL	1	13	13	9	7	8	6	8	9	6	6	5	9	7	8	6	8	9	6	6	5	9	7	8	7	9	10	6	6	5
Fibrinogen gamma chain	FGG	1	17	17	3	5	6	12	11	12	3	4	7	3	5	6	12	11	12	3	4	7	3	5	6	14	14	15	4	5	8
Glycogen phosphorylase, brain form	PYGB	1	26	19	4	7	8	1	1	0	8	7	8	6	5	5	0	0	0	4	4	4	6	5	6	0	0	0	5	6	5
Alpha/beta hydrolase domain-containing protein 14B	ABHD14B	1	7	7	2	1	3	0	0	0	5	5	4	2	1	3	0	0	0	5	5	4	2	1	3	0	0	0	5	6	5
Proteasome subunit alpha type-2	PSMA2	1	9	9	4	7	5	1	1	2	4	4	5	6	7	5	1	1	2	4	4	5	9	9	8	1	1	2	5	5	6
Transthyretin	TTR	1	9	9	4	5	7	2	2	1	5	5	5	3	5	7	2	2	1	5	5	5	3	5	7	2	2	1	5	6	5
Tissue alpha-L-fucosidase	FUCA1	1	10	10	4	3	2	2	3	4	3	5	4	4	3	2	2	3	4	3	5	4	4	3	2	2	3	4	5	6	5
GDP-L-fucose synthase	TSTA3	1	16	16	4	9	8	3	4	3	6	5	5	10	9	8	3	4	3	6	5	5	10	10	9	3	4	3	6	5	5
Heme-binding protein 2	HEBP2	1	11	11	6	8	6	3	4	3	6	5	5	6	8	6	3	4	3	6	5	5	7	9	7	3	4	3	6	5	5
Phospholipase A2, membrane associated	PLA2G2A	1	6	6	4	3	3	3	2	3	4	3	3	5	3	3	3	2	3	4	3	3	7	6	5	4	3	4	6	5	5
Golgi apparatus protein 1	GLG1	1	12	12	2	1	2	4	5	3	6	5	5	2	1	2	4	5	3	6	5	5	2	1	2	4	5	3	6	5	5
Actin-related protein 3	ACTR3	3	18	18	10	8	9	3	6	4	5	6	5	10	8	9	3	6	4	5	6	5	11	8	11	3	6	4	5	6	5
Peroxiredoxin-2	PRDX2	1	13	12	4	6	8	7	3	6	6	5	7	6	5	7	6	2	5	5	5	6	6	6	7	7	2	5	5	5	6
Beta-mannosidase	MANBA	1	11	11	4	3	3	4	4	6	5	5	6	4	3	3	4	4	6	5	5	6	4	3	3	4	4	6	5	5	6
Nucleoside diphosphate kinase B	NME2;NME2P1	2	10	6	9	6	7	4	5	3	5	4	4	6	3	4	2	2	1	2	2	2	14	11	12	5	6	4	6	5	5
Carbonyl reductase [NADPH] 1	CBR1	1	14	12	9	10	10	6	4	5	4	7	5	7	8	8	4	2	3	2	5	3	10	12	11	6	4	5	4	7	5
Vinculin	VCL	1	19	19	4	1	0	5	6	5	6	5	5	1	1	0	5	6	5	6	5	5	1	1	0	5	6	5	6	5	5
Peptidyl-glycine alpha-amidating monooxygenase	PAM	1	10	10	4	3	2	6	4	7	5	5	6	3	3	2	6	4	7	5	5	6	3	3	2	6	4	7	5	5	6
Malate dehydrogenase, cytoplasmic	MDH1	1	14	14	7	8	9	6	6	6	5	5	6	7	8	9	6	6	6	5	5	6	9	11	12	7	7	7	5	5	6
Alpha-2-macroglobulin	A2M	2	71	65	15	12	16	7	7	9	4	7	5	14	11	15	6	6	9	4	6	4	17	13	17	7	7	9	4	7	5
Polypeptide N-acetylgalactosaminyltransferase 6	GALNT6	1	10	9	0	1	0	7	8	7	4	4	6	0	0	0	6	7	6	3	3	5	0	1	0	9	8	8	4	5	7
Ig gamma-3 chain C region	IGHG3	1	12	7	7	7	7	8	8	8	5	2	3	3	3	3	4	3	3	1	0	0	10	9	10	13	12	12	7	4	5
Protein S100-A7	S100A7	1	13	9	4	4	4	11	12	11	4	5	7	3	2	2	8	8	7	2	2	4	5	4	4	14	19	18	4	5	7
Tryptophan--tRNA ligase, cytoplasmic	WARS	1	10	10	4	2	2	0	0	0	4	4	4	3	2	2	0	0	0	4	4	4	4	3	3	0	0	0	5	5	5
UTP--glucose-1-phosphate uridylyltransferase	UGP2	1	16	16	4	0	0	0	0	0	5	5	4	1	0	0	0	0	0	5	5	4	1	0	0	0	0	0	5	5	5
Calpain-2 catalytic subunit	CAPN2	1	23	23	0	0	0	0	0	0	5	5	5	0	0	0	0	0	0	5	5	5	0	0	0	0	0	0	5	5	5
Inorganic pyrophosphatase	PPA1	1	16	16	4	7	5	0	1	2	5	4	6	9	7	5	0	1	2	5	4	6	9	7	5	0	1	2	5	4	6
Aminopeptidase B	RNPEP	1	24	24	4	17	17	1	1	2	6	4	4	16	17	17	1	1	2	6	4	4	16	18	17	1	1	2	6	4	5
Catechol O-methyltransferase	COMT	1	15	15	8	9	8	1	1	2	4	4	5	8	9	8	1	1	2	4	4	5	11	11	11	1	1	2	5	5	5
Kininogen-1	KNG1	4	23	23	13	15	13	2	4	2	5	5	5	13	15	13	2	4	2	5	5	5	13	17	14	2	4	2	5	5	5
Alcohol dehydrogenase class-3	ADH5	1	11	11	8	7	8	3	2	2	4	4	4	8	7	8	3	2	2	4	4	4	10	9	9	4	2	2	5	5	5
Sialidase-1	NEU1	1	8	8	4	0	0	1	2	3	3	4	5	0	0	0	1	2	3	3	4	5	0	0	0	1	3	4	4	5	6
N-acetylglucosamine-1-phosphotransferase subunit gamma	GNPTG	1	6	6	1	2	1	4	2	3	6	4	5	1	2	1	4	2	3	6	4	5	1	2	1	4	2	3	6	4	5
Ig kappa chain V-III region B6	IGKV3-20	1	5	3	4	4	3	3	3	4	4	4	4	2	2	1	2	2	2	2	2	2	4	4	3	3	3	4	5	5	5
Slit homolog 3 protein	SLIT3	1	9	9	4	0	0	4	3	3	4	4	7	0	0	0	4	3	3	4	4	7	0	0	0	4	3	3	4	4	7
Prominin-1	PROM1	1	12	12	4	1	1	4	3	3	5	5	5	5	1	1	4	3	3	5	5	5	5	1	1	5	3	3	5	5	5
14-3-3 protein beta/alpha	YWHAB	1	16	11	4	7	7	6	5	5	5	7	7	6	5	6	4	4	4	4	5	4	7	6	6	4	4	4	5	5	5
WAP four-disulfide core domain protein 2	WFDC2	1	5	5	4	4	5	3	4	3	4	4	4	5	4	5	3	4	3	4	4	4	5	4	6	3	5	4	5	4	6
Di-N-acetylchitobiase	CTBS	1	9	9	5	4	5	5	3	4	4	5	6	5	4	5	5	3	4	4	5	6	5	4	5	5	3	4	4	5	6
Ig lambda-1 chain C regions	IGLC1/L5	2	8	1	4	5	5	6	6	4	4	3	3	1	1	1	1	1	1	1	1	1	3	3	2	4	3	6	6	5	4
Collagen alpha-1(VI) chain	COL6A1	1	13	13	1	1	1	4	5	6	5	4	5	1	1	1	4	5	6	5	4	5	1	1	1	4	6	6	6	4	5
Glucosidase 2 subunit beta	PRKCSH	1	12	12	4	6	5	6	7	5	6	4	5	5	6	5	6	7	5	6	4	5	5	6	5	6	7	5	6	4	5
Nicotinamide phosphoribosyltransferase	NAMPT	1	15	15	4	2	0	8	7	5	5	4	5	1	2	0	8	7	5	5	4	5	1	2	0	8	8	5	6	4	5
Kynureninase	KYNU	1	13	13	8	7	9	0	0	0	5	4	4	8	7	9	0	0	0	5	4	4	12	11	12	0	0	0	5	5	4

Pyridoxal kinase	PDXK	1	11	11	4	6	8	0	0	0	4	5	5	8	6	8	0	0	0	4	5	5	9	6	8	0	0	0	4	5	5
Talin-1	TLN1	1	33	30	4	0	0	2	0	1	5	5	4	0	0	0	2	0	1	4	5	4	0	0	0	2	0	1	5	5	4
Glyoxalase domain-containing protein 4	GLOD4	1	14	14	9	5	5	2	1	1	4	5	5	9	5	5	2	1	1	4	5	5	11	7	7	2	1	1	4	5	5
Proteasome subunit beta type-1	PSMB1	1	9	9	4	7	6	3	3	3	5	4	2	7	7	6	3	3	3	5	4	2	8	10	8	3	3	3	6	5	3
Ig heavy chain V-III region JON	IGHV3-21	6	7	3	2	2	4	4	1	3	3	4	4	0	0	1	2	0	1	1	2	2	3	2	5	5	2	3	4	5	5
Prothrombin	F2	2	19	19	12	11	10	5	3	3	6	3	5	12	11	10	5	3	3	6	3	5	14	12	10	5	3	3	6	3	5
Beta-2-glycoprotein 1	APOH	2	12	12	8	7	6	5	3	5	5	4	4	8	7	6	5	3	5	5	4	4	11	7	7	5	3	5	5	4	5
14-3-3 protein theta	YWHAQ	1	13	10	4	4	4	7	5	5	5	5	5	3	3	3	5	4	4	4	4	4	3	3	3	5	4	4	5	5	4
60S acidic ribosomal protein P2	RPLP2	1	7	6	4	5	5	7	6	7	4	3	3	4	5	5	6	6	6	4	3	3	6	8	6	9	9	10	7	4	3
Complement C4-A	C4A	3	60	2	7	7	9	15	13	16	5	4	5	1	0	1	1	1	1	0	0	0	7	7	9	15	14	16	5	4	5
Caspase-14	CASP14	1	18	18	3	4	2	14	11	14	3	4	4	3	4	2	14	11	14	3	4	4	3	4	2	16	13	17	4	5	5
Glycerol-3-phosphate dehydrogenase 1-like protein	GPD1L	1	13	12	1	1	3	0	0	0	5	3	5	1	1	3	0	0	0	5	3	5	1	1	3	0	0	0	5	3	5
Glutathione synthetase	GSS	1	21	21	12	10	11	1	0	0	5	3	5	12	10	11	1	0	0	5	3	5	12	10	11	1	0	0	5	3	5
Niban-like protein 1	FAM129B	1	14	14	2	2	2	0	1	1	4	4	5	2	2	2	0	1	1	4	4	5	2	2	3	0	1	1	4	4	5
High mobility group protein B1	HMGB1/P1	3	12	9	11	8	9	1	2	2	3	4	4	8	6	6	1	2	2	3	3	3	12	9	10	1	2	2	4	5	4
Glutathione peroxidase 3	GPX3	1	5	5	1	1	2	1	3	2	5	4	4	1	1	2	1	3	2	5	4	4	1	1	2	1	3	2	5	4	4
Proteasome subunit alpha type-6	PSMA6	1	14	14	4	11	11	3	2	2	5	4	4	11	11	11	3	2	2	5	4	4	12	15	15	3	2	2	5	4	4
Adipogenesis regulatory factor	ADIRF	1	6	6	5	5	4	2	3	4	4	4	5	5	5	4	2	3	4	4	4	5	6	6	6	2	3	4	4	4	5
Ig kappa chain V-I region HK102	IGKV1-5	1	4	4	2	2	1	3	3	3	3	3	4	2	2	1	3	3	3	3	3	4	2	2	1	3	3	3	3	4	6
Transaldolase	TALDO1	1	13	13	4	7	6	2	3	3	2	5	4	5	7	6	2	3	3	2	5	4	6	8	7	3	3	4	3	6	4
Angiogenin	ANG	1	6	6	3	4	2	3	2	4	3	3	4	3	4	2	3	2	4	3	3	4	4	5	3	3	2	5	5	4	4
Carboxypeptidase Q	CPQ	1	5	5	0	0	0	3	3	4	3	5	5	0	0	0	3	3	4	3	5	5	0	0	0	3	3	4	3	5	5
Protein deglycase DJ-1	PARK7	1	12	12	4	8	7	4	4	2	3	3	4	8	8	7	4	4	2	3	3	4	11	12	11	5	5	3	4	4	5
Thrombospondin-1	THBS1	3	11	11	0	0	0	3	2	5	3	3	4	0	0	0	3	2	5	3	3	4	0	0	0	4	2	7	4	4	5
Zymogen granule protein 16 homolog B	ZG16B	1	8	8	4	4	4	4	5	4	2	4	3	3	4	4	4	5	4	2	4	3	5	5	5	5	6	5	3	5	5
Thymidine phosphorylase	TYMP	1	19	19	4	0	1	13	11	11	5	3	5	0	0	1	13	11	11	5	3	5	0	0	1	15	13	13	5	3	5
Protein Niban	FAM129A	1	16	16	0	0	0	0	0	0	4	4	4	0	0	0	0	0	0	4	4	4	0	0	0	0	0	0	4	4	4
Angiopoietin-related protein 1	ANGPTL1	2	8	8	0	0	0	0	1	1	3	3	4	0	0	0	0	1	1	3	3	4	0	0	0	0	1	1	4	3	5
Nucleosome assembly protein 1-like 4	NAP1L4	1	9	7	4	6	5	1	1	3	4	5	3	4	4	5	1	1	3	2	3	1	4	7	5	1	1	3	4	5	3
Eukaryotic translation initiation factor 5A-1	EIF5A;EIF5AL1;EIF5A2	3	10	10	7	6	6	2	2	2	4	5	3	7	6	6	2	2	2	4	5	3	8	8	7	2	2	2	4	5	3
Rho GDP-dissociation inhibitor 1	ARHGDIA	1	13	13	8	3	6	2	2	2	3	5	4	8	3	6	2	2	2	3	5	4	9	3	7	2	2	2	3	5	4
Stress-induced-phosphoprotein 1	STIP1	1	21	21	4	13	11	3	4	4	4	5	3	11	13	11	3	4	4	4	5	3	12	14	14	3	4	4	4	5	3
Heterogeneous nuclear ribonucleoproteins A2/B1	HNRNPA2B1	1	17	17	8	7	6	4	5	3	3	5	4	8	7	6	4	5	3	3	5	4	8	8	7	4	5	3	3	5	4
Proteasome subunit alpha type-5	PSMA5	1	8	8	4	6	6	3	2	4	2	5	2	5	6	6	3	2	4	2	5	2	6	7	8	4	3	5	3	6	3
Attractin	ATRN	1	8	8	1	2	2	3	3	4	3	4	2	1	2	2	3	3	4	3	4	2	1	3	3	4	3	5	4	5	3
Biotinidase	BTD	1	5	5	2	1	1	3	3	3	4	3	2	2	1	1	3	3	3	4	3	2	3	1	1	4	4	4	5	4	3
Prolyl endopeptidase	PREP	1	23	23	4	14	15	5	4	5	4	4	4	19	14	15	5	4	5	4	4	4	20	15	16	5	4	5	4	4	4
GTP-binding nuclear protein Ran	RAN	1	9	9	4	4	3	5	3	4	3	3	3	3	4	3	5	3	4	3	3	3	4	4	3	6	4	5	4	4	4
Cellular retinoic acid-binding protein 2	CRABP2	1	7	7	4	4	3	5	5	7	3	3	3	4	4	3	5	5	7	3	3	3	5	5	4	7	7	9	4	4	4
Guanine nucleotide-binding protein subunit beta-2-like 1	GNB2L1	1	15	15	13	11	11	8	8	9	4	4	4	13	11	11	8	8	9	4	4	4	16	14	13	9	8	9	4	4	4
Serine hydroxymethyltransferase, cytosolic	SHMT1	1	14	13	4	7	10	0	0	0	4	3	4	7	6	9	0	0	0	4	3	4	7	7	10	0	0	0	4	3	4
Cysteine-rich protein 2	CRIP2	1	4	4	4	4	4	0	0	0	3	4	4	4	4	4	0	0	0	3	4	4	5	4	4	0	0	0	3	4	4
Phosphoglucomutase-1	PGM1	1	10	10	4	1	2	0	0	0	4	4	3	2	1	2	0	0	0	4	4	3	2	1	2	0	0	0	4	4	3
Glutathione S-transferase theta-1	GSTT1	1	10	10	0	0	0	0	0	0	3	3	5	0	0	0	0	0	0	3	3	5	0	0	0	0	0	0	3	3	5
EF-hand domain-containing protein D2	EFHD2	2	16	16	9	8	6	0	1	1	4	4	3	9	8	6	0	1	1	4	4	3	9	8	6	0	1	1	4	4	3
Protein ERGIC-53	LMAN1	1	8	8	0	0	0	1	1	1	3	2	5	0	0	0	1	1	1	3	2	5	0	0	0	1	1	1	3	2	6
V-type proton ATPase catalytic subunit A	ATP6V1A	1	17	17	2	3	3	1	1	2	4	3	4	2	3	3	1	1	2	4	3	4	2	3	3	1	1	2	4	3	4
Protein S100-A12	S100A12	1	6	6	4	3	3	2	2	2	2	2	2	5	3	3	2	2	2	2	2	2	8	6	7	2	2	3	4	3	4
T-complex protein 1 subunit beta	CCT2	1	22	22	1	0	0	3	2	4	4	3	4	1	0	0	3	2	4	4	3	4	1	0	0	3	2	4	4	3	4
Myosin regulatory light chain 12A	MYL12A/B	3	7	7	7	7	6	4	3	4	4	3	4	7	7	6	4	3	4	4	3	4	7	7	6	4	3	4	4	3	4

Protein NDRG2	NDRG2	1	10	9	4	5	5	5	3	4	3	3	5	3	5	5	5	3	4	3	3	5	3	5	5	5	3	4	3	3	5
Ribonuclease 4	RNASE4	1	7	7	4	2	3	3	3	3	2	2	3	2	2	3	3	3	3	2	2	3	3	3	4	4	4	4	3	3	5
Dipeptidyl peptidase 1	CTSC	1	9	9	4	5	5	5	5	4	3	2	4	4	5	5	5	5	4	3	2	4	4	5	5	6	5	4	4	2	5
T-complex protein 1 subunit theta	CCT8	1	18	17	0	0	0	6	5	7	4	3	4	0	0	0	6	5	7	4	3	4	0	0	0	6	5	7	4	3	4
Filaggrin	FLG	2	15	15	3	4	3	10	8	9	2	2	4	3	4	3	10	8	9	2	2	4	4	5	4	13	10	11	3	3	5
Adenylate kinase isoenzyme 1	AK1	1	7	7	2	2	2	0	0	0	3	3	3	2	2	2	0	0	0	3	3	3	3	3	2	0	0	0	3	3	4
ATP-dependent 6-phosphofructokinase, liver type	PFKL	1	19	17	4	0	1	0	0	0	5	3	2	0	0	0	0	0	0	4	2	2	0	0	1	0	0	0	5	3	2
Adseverin	SCIN	1	27	27	4	0	1	0	0	0	3	2	5	0	0	1	0	0	0	3	2	5	0	0	1	0	0	0	3	2	5
Thymosin beta-10	TMSB10	2	5	5	5	4	5	0	1	1	3	2	3	5	4	5	0	1	1	3	2	3	10	8	8	0	1	1	4	2	4
Beta-1,3-N-acetylglucosaminyltransferase lunatic fringe	LFNG	1	6	6	1	1	1	1	0	2	3	3	3	1	1	1	1	0	2	3	3	3	1	1	1	1	0	2	3	3	4
Myotrophin	MTPN	1	9	9	7	7	8	2	1	1	4	3	3	7	7	8	2	1	1	4	3	3	9	7	9	2	1	1	4	3	3
Calpastatin	CAST	1	15	15	8	8	7	2	2	0	4	3	3	8	8	7	2	2	0	4	3	3	8	8	7	2	2	0	4	3	3
F-actin-capping protein subunit alpha-2	CAPZA2	1	8	7	1	3	4	1	1	2	3	3	3	1	2	3	1	1	2	3	3	3	1	2	3	1	1	2	3	3	4
Complement factor I	CFI	2	11	11	6	5	6	1	2	2	4	3	3	6	5	6	1	2	2	4	3	3	6	5	6	1	2	2	4	3	3
Specifically androgen-regulated gene protein	SARG	1	10	10	4	6	7	2	2	2	3	3	4	7	6	7	2	2	2	3	3	4	7	6	7	2	2	2	3	3	4
Adenine phosphoribosyltransferase	APRT	1	13	13	7	6	6	2	2	2	4	4	2	7	6	6	2	2	2	4	4	2	7	6	6	2	2	2	4	4	2
Calcium-regulated heat stable protein 1	CARHSP1	1	4	4	4	3	2	1	1	1	3	2	2	4	3	2	1	1	1	3	2	2	5	4	3	2	2	2	4	3	3
Lactadherin	MFGE8	1	7	7	0	0	0	1	1	4	3	3	4	0	0	0	1	1	4	3	3	4	0	0	0	1	1	4	3	3	4
Glyoxylate reductase/hydroxypyruvate reductase	GRHPR	1	9	9	7	6	6	3	2	2	3	3	4	7	6	6	3	2	2	3	3	4	7	6	6	3	2	2	3	3	4
Platelet-activating factor acetylhydrolase IB subunit alpha	PAFAH1B1	1	13	13	4	10	7	2	3	3	3	3	4	9	10	7	2	3	3	3	3	4	10	11	8	2	3	3	3	3	4
Serine/threonine-protein phosphatase PP1-beta catalytic subunit	PPP1CB	1	12	4	4	7	6	2	3	3	3	3	4	4	3	2	1	1	2	1	2	2	8	7	6	2	3	3	3	3	4
Latexin	LXN	1	4	4	2	2	2	2	2	2	2	3	2	2	2	2	2	2	2	2	3	2	3	3	2	2	3	3	3	4	3
Dystroglycan	DAG1	1	5	5	1	2	2	2	3	3	3	3	4	1	2	2	2	3	3	3	3	4	1	2	2	2	3	3	3	3	4
Protein FAM3D	FAM3D	1	6	6	1	1	1	4	2	2	2	3	4	1	1	1	4	2	2	2	3	4	1	1	1	4	2	2	3	3	4
Adenosylhomocysteinase	AHCY	1	13	13	10	9	10	3	3	3	4	3	3	10	9	10	3	3	3	4	3	3	11	10	11	3	3	3	4	3	3
Immunoglobulin kappa variable 1-6	IGKV1-6	1	3	0	2	2	2	2	2	3	2	2	3	0	0	0	0	0	0	0	0	0	3	3	4	3	3	4	3	3	4
Eukaryotic initiation factor 4A-I	EIF4A1	1	12	7	0	0	0	4	4	3	2	3	4	0	0	0	1	1	1	0	0	2	0	0	0	5	5	3	2	3	5
Zinc finger protein 185	ZNF185	1	17	17	4	7	7	4	4	6	3	4	3	6	7	7	4	4	6	3	4	3	7	7	7	4	4	6	3	4	3
Glucosamine-6-phosphate isomerase 1	GNPDA1	1	12	7	10	7	9	0	0	0	4	2	3	5	2	4	0	0	0	3	1	2	11	9	10	0	0	0	4	2	3
Calcyphosin	CAPS	1	14	14	10	9	7	0	0	0	2	1	3	10	9	7	0	0	0	2	1	3	11	10	8	0	0	0	3	2	4
Quinone oxidoreductase	CRYZ	1	10	10	9	7	6	0	0	0	2	3	3	9	7	6	0	0	0	2	3	3	9	8	6	0	0	0	3	3	3
Coronin-1B	CORO1B	1	12	12	4	4	6	0	0	0	3	3	3	4	4	6	0	0	0	3	3	3	4	4	6	0	0	0	3	3	3
Protein phosphatase 1 regulatory subunit 7	PPP1R7	1	11	11	4	4	4	0	0	0	4	3	2	3	4	4	0	0	0	4	3	2	3	4	4	0	0	0	4	3	2
Proteasome subunit beta type-8	PSMB8	1	10	10	4	8	9	1	0	1	3	3	3	7	8	9	1	0	1	3	3	3	7	8	9	1	0	1	3	3	3
Ras-related protein Rap-1b	RAP1B	2	10	2	2	1	1	0	1	1	4	2	3	1	1	1	0	1	1	1	0	1	2	1	1	0	1	1	4	2	3
Ig heavy chain V-II region NEWM	IGHV4-61	11	3	3	1	1	2	1	1	1	1	1	1	1	1	2	1	1	1	1	1	1	1	1	2	1	1	1	3	3	3
Calpain small subunit 1	CAPNS1	1	16	15	1	1	1	1	0	2	3	3	3	1	1	1	1	0	2	3	3	3	1	1	1	1	0	2	3	3	3
HLA class I histocompatibility antigen, Cw-17 alpha chain	HLA-C	1	9	6	2	2	2	1	1	2	3	3	3	0	0	0	0	0	1	2	2	2	2	2	2	1	1	2	3	3	3
Proteasome subunit alpha type-3	PSMA3	1	10	10	4	8	9	2	1	2	4	3	2	6	8	9	2	1	2	4	3	2	6	8	10	2	1	2	4	3	2
Twinfilin-1	TWF1	1	13	12	4	2	3	3	1	1	3	3	3	1	1	2	3	1	1	2	3	2	2	2	3	3	1	1	3	3	3
4-trimethylaminobutyraldehyde dehydrogenase	ALDH9A1	1	14	14	11	9	11	2	2	2	4	2	3	11	9	11	2	2	2	4	2	3	12	9	12	2	2	2	4	2	3
Thioredoxin-like protein 1	TXNL1	1	7	7	4	4	3	2	2	2	3	3	3	5	4	3	2	2	2	3	3	3	6	5	4	2	2	2	3	3	3
Thioredoxin domain-containing protein 17	TXNDC17	1	7	7	4	4	4	2	2	2	3	3	3	3	4	4	2	2	2	3	3	3	4	5	5	2	2	2	3	3	3
Peptidyl-prolyl cis-trans isomerase B	PPIB	1	12	12	4	2	3	2	1	1	3	3	3	2	2	3	2	1	1	3	3	3	2	2	3	2	2	2	3	3	3
Histidine triad nucleotide-binding protein 1	HINT1	1	10	10	7	7	6	2	2	3	3	2	2	7	7	6	2	2	3	3	2	2	8	7	8	2	2	3	3	3	3
Ubiquitin thioesterase OTUB1	OTUB1	1	8	8	4	2	4	3	3	1	4	3	2	2	2	4	3	3	1	4	3	2	2	2	4	3	3	1	4	3	2
T-complex protein 1 subunit alpha	TCP1	1	20	20	4	0	0	2	3	2	4	2	3	0	0	0	2	3	2	4	2	3	0	0	0	2	3	2	4	2	3
Superoxide dismutase [Cu-Zn]	SOD1	1	8	8	4	5	7	3	2	3	4	3	2	5	5	7	3	2	3	4	3	2	5	6	7	3	2	3	4	3	2
Ig kappa chain V-III region POM	IGKV3-15	1	2	0	4	2	2	2	2	2	2	2	2	0	0	0	0	0	0	0	0	0	2	3	3	3	3	3	2	3	4
Fructose-bisphosphate aldolase C	ALDOC	1	7	4	7	5	5	4	4	5	3	4	4	4	2	2	2	2	2	1	1	1	9	6	6	3	3	4	3	3	3

Glutaredoxin-1	GLRX	1	6	6	3	4	4	3	4	3	4	3	2	3	4	4	3	4	3	4	3	2	3	4	4	3	4	3	4	3	2
Ig kappa chain V-IV region	IGKV4-1	1	4	4	2	3	2	2	4	4	3	2	3	2	3	2	2	4	4	3	2	3	2	3	2	2	4	4	3	2	4
Cystatin-B	CSTB	1	4	4	4	4	3	3	2	3	2	2	2	4	4	3	3	2	3	2	2	2	6	6	5	4	3	4	3	3	3
Omega-amidase NIT2	NIT2	1	13	13	4	7	11	4	5	5	3	4	2	11	7	11	4	5	5	3	4	2	13	9	14	4	5	5	3	4	2
Alpha-N-acetylglucosaminidase	NAGLU	1	9	9	4	4	2	7	2	5	2	3	4	3	4	2	7	2	5	2	3	4	3	4	2	7	2	5	2	3	4
40S ribosomal protein SA	RPSA	1	10	10	4	7	5	6	6	4	4	2	3	7	7	5	6	6	4	4	2	3	7	7	6	6	6	4	4	2	3
Eukaryotic translation initiation factor 6	EIF6	1	8	8	4	3	2	5	4	5	4	3	2	4	3	2	5	4	5	4	3	2	4	3	2	6	5	6	4	3	2
Heterogeneous nuclear ribonucleoprotein A1	HNRNPA1/L2	3	9	9	5	6	6	6	5	6	3	3	3	5	6	6	6	5	6	3	3	3	5	6	6	7	6	6	3	3	3
Calreticulin	CALR	1	13	13	7	8	9	7	7	5	3	3	2	7	8	9	7	7	5	3	3	2	8	9	10	9	8	6	4	3	2
Periplakin	PPL	1	66	66	4	7	13	38	34	41	2	3	4	10	7	13	38	34	41	2	3	4	10	7	13	42	36	44	2	3	4
Cytosolic 10-formyltetrahydrofolate dehydrogenase	ALDH1L1	2	27	27	13	14	14	0	0	0	2	3	3	13	14	14	0	0	0	2	3	3	14	16	15	0	0	0	2	3	3
Apolipoprotein A-IV	APOA4	2	31	31	4	4	3	0	0	0	3	3	2	4	4	3	0	0	0	3	3	2	4	4	3	0	0	0	3	3	2
Kinesin-like protein KIF21A	KIF21A	4	22	22	3	1	2	0	0	0	2	3	2	3	1	2	0	0	0	2	3	2	3	1	2	0	0	0	2	4	2
Sialic acid synthase	NANS	1	12	12	4	3	6	1	1	0	2	3	3	4	3	6	1	1	0	2	3	3	4	6	8	1	1	0	2	3	3
Peptidyl-prolyl cis-trans isomerase FKBP1A	FKBP1A	2	6	6	3	4	4	1	0	1	2	3	2	3	4	4	1	0	1	2	3	2	3	5	4	1	0	1	2	4	2
Cysteine-rich protein 1	CRIP1	1	3	3	3	3	3	0	1	1	3	2	2	3	3	3	0	1	1	3	2	2	4	4	4	0	1	1	4	2	2
Complement component C7	C7	2	13	13	3	2	2	1	1	0	3	2	3	3	2	2	1	1	0	3	2	3	3	3	2	1	1	0	3	2	3
Collagen alpha-3(IX) chain	COL9A3	1	3	3	0	0	0	1	0	1	3	2	3	0	0	0	1	0	1	3	2	3	0	0	0	1	0	1	3	2	3
Ubiquitin carboxyl-terminal hydrolase 5	USP5	1	19	19	4	0	0	1	1	1	3	3	2	1	0	0	1	1	1	3	3	2	1	0	0	1	1	1	3	3	2
Palmitoyl-protein thioesterase 1	PPT1	1	3	3	4	0	0	1	1	1	3	2	3	0	0	0	1	1	1	3	2	3	0	0	0	1	1	1	3	2	3
Alkaline phosphatase, tissue-nonspecific isozyme	ALPL	1	6	6	2	1	2	1	1	2	2	3	3	2	1	2	1	1	2	2	3	3	2	1	3	1	1	2	2	3	3
Renin receptor	ATP6AP2	1	4	4	0	0	0	1	1	2	2	3	3	0	0	0	1	1	2	2	3	3	0	0	0	1	1	2	2	3	3
Glucose-6-phosphate 1-dehydrogenase	G6PD	1	17	17	8	6	6	2	2	1	3	2	3	8	6	6	2	2	1	3	2	3	8	6	6	2	2	1	3	2	3
Immunoglobulin heavy variable 3-72	IGHV3-72	1	6	2	2	2	0	1	2	2	2	3	3	1	2	0	1	1	1	1	1	1	2	2	0	1	2	2	2	3	3
Secretoglobin family 1D member 1	SCGB1D1	1	5	5	4	3	4	2	1	3	1	3	3	3	3	4	2	1	3	1	3	3	3	3	4	2	1	3	2	3	3
Cathepsin F	CTSF	1	6	6	0	0	0	2	3	1	3	2	3	0	0	0	2	3	1	3	2	3	0	0	0	2	3	1	3	2	3
Poly(rC)-binding protein 1	PCBP1	1	10	8	4	4	2	2	3	2	2	3	3	2	3	2	2	2	2	2	2	2	3	4	2	2	3	2	2	3	3
Tumor necrosis factor ligand superfamily member 13	TNFSF13	1	6	6	4	3	3	2	3	2	3	3	2	3	3	3	2	3	2	3	3	2	3	3	3	2	3	2	3	3	2
CD59 glycoprotein	CD59	1	5	5	2	1	2	1	1	2	2	2	1	2	1	2	1	1	2	2	2	1	3	2	2	2	2	3	3	3	2
Actin-related protein 2/3 complex subunit 4	ARPC4	1	7	7	3	3	4	2	3	3	3	3	2	3	3	4	2	3	3	3	3	2	3	3	4	2	3	3	3	3	2
Ras-related protein Rab-1A	RAB1A	3	8	4	1	1	1	3	3	2	3	2	3	0	0	0	0	1	0	1	0	1	1	1	1	3	3	2	3	2	3
Serine/threonine-protein phosphatase 2A 65 kDa regulatory subunit A alpha isoform	PPP2R1A	1	12	8	4	0	0	2	2	4	2	3	3	0	0	0	1	1	2	0	1	2	0	0	0	2	2	4	2	3	3
YEATS domain-containing protein 2	YEATS2	1	1	1	4	1	1	1	1	1	1	1	1	1	1	1	1	1	1	1	1	1	2	4	3	3	3	3	3	3	2
Epididymal secretory protein E1	NPC2	1	4	4	4	2	2	3	3	2	2	3	3	3	2	2	3	3	2	2	3	3	3	2	2	3	4	2	2	3	3
Dynactin subunit 2	DCTN2	1	10	10	5	5	6	5	4	2	3	2	3	5	5	6	5	4	2	3	2	3	5	5	6	5	4	2	3	2	3
Proline-rich protein 27	PRR27	1	3	3	4	2	2	3	3	3	1	3	1	0	2	2	3	3	3	1	3	1	0	2	2	4	5	4	2	5	1
Dynein light chain 1, cytoplasmic	DYNLL1	1	4	1	0	1	2	4	3	3	2	3	2	0	0	1	1	1	1	0	1	1	0	1	2	5	4	4	3	3	2
Elafin	PI3	1	6	6	4	3	2	5	3	4	2	2	2	2	3	2	5	3	4	2	2	2	2	3	2	6	4	5	3	2	3
Endoplasmin	HSP90B1	2	23	21	1	0	1	8	6	9	5	4	5	0	0	1	6	4	7	3	2	3	0	0	1	6	4	7	3	2	3
Apolipoprotein D	APOD	1	6	6	2	2	2	6	6	6	3	2	3	2	2	2	6	6	6	3	2	3	2	2	2	6	6	6	3	2	3
Malate dehydrogenase, mitochondrial	MDH2	1	15	15	11	8	8	8	8	9	2	3	3	11	8	8	8	8	9	2	3	3	13	9	9	9	9	10	2	3	3
Vimentin	VIM	2	32	28	16	17	16	10	10	9	2	3	3	15	14	13	9	8	8	2	3	3	18	19	19	11	10	10	2	3	3
Prelamin-A/C	LMNA	1	31	31	4	2	3	23	23	20	2	3	3	4	2	3	23	23	20	2	3	3	4	2	3	30	30	25	2	3	3
1,4-alpha-glucan-branching enzyme	GBE1	1	11	11	7	5	5	0	0	0	3	2	2	7	5	5	0	0	0	3	2	2	8	6	6	0	0	0	3	2	2
S-methyl-5-thioadenosine phosphorylase	MTAP	1	7	7	4	3	3	0	0	0	2	2	2	4	3	3	0	0	0	2	2	2	4	3	3	0	0	0	3	2	2
L-lactate dehydrogenase B chain	LDHB	1	14	12	6	3	5	1	1	1	3	4	3	4	1	3	0	0	0	2	3	2	4	1	3	0	0	0	2	3	2
Hepatoma-derived growth factor	HDGF	3	6	5	4	4	4	0	0	1	1	2	1	4	4	4	0	0	1	1	2	1	4	5	5	0	0	1	2	3	2
Proteasome subunit beta type-3	PSMB3	1	7	7	4	3	5	1	0	0	1	2	1	4	3	5	1	0	0	1	2	1	5	4	5	1	0	0	2	3	2
EGF-containing fibulin-like extracellular matrix protein 1	EFEMP1	1	6	6	1	1	0	0	1	0	2	2	3	1	1	0	0	1	0	2	2	3	1	1	0	0	1	0	2	2	3
Heterogeneous nuclear ribonucleoprotein D0	HNRNPD	1	11	11	4	2	3	1	1	0	2	2	3	4	2	3	1	1	0	2	2	3	5	3	3	1	1	0	2	2	3

Neutrophil defensin 3	DEFA3/A1	2	4	4	2	3	3	1	0	1	3	2	2	2	3	3	1	0	1	3	2	2	2	3	3	1	0	1	3	2	2	2
Vesicle-fusing ATPase	NSF	1	20	19	4	0	0	1	1	0	3	1	3	1	0	0	1	1	0	3	1	3	1	0	0	1	1	0	3	1	3	3
Proteasome subunit alpha type-1	PSMA1	1	13	13	4	7	8	1	1	1	2	2	3	8	7	8	1	1	1	2	2	3	8	7	9	1	1	1	2	2	3	3
Inter-alpha-trypsin inhibitor heavy chain H2	ITIH2	2	19	19	5	4	4	1	2	0	2	2	3	5	4	4	1	2	0	2	2	3	5	4	4	1	2	0	2	2	3	3
Cadherin-1	CDH1	1	9	9	2	3	2	1	1	1	2	2	3	2	3	2	1	1	1	2	2	3	2	3	2	1	1	1	2	2	3	3
Cullin-associated NEDD8-dissociated protein 1	CAND1	1	12	12	1	1	0	1	1	1	3	1	3	1	1	0	1	1	1	3	1	3	1	1	0	1	1	1	3	1	3
Vacuolar protein sorting-associated protein 4B	VPS4B	2	9	8	0	0	0	1	1	1	2	3	2	0	0	0	1	1	1	2	3	2	0	0	0	1	1	1	2	3	2
Beta-1,4-galactosyltransferase 1	B4GALT1	1	4	4	0	0	0	1	1	1	2	2	2	0	0	0	1	1	1	2	2	2	0	0	0	1	1	1	2	3	2
T-complex protein 1 subunit delta	CCT4	1	14	14	0	0	0	0	2	1	3	3	1	0	0	0	0	2	1	3	3	1	0	0	0	0	2	1	3	3	1
N(G),N(G)-dimethylarginine dimethylaminohydrolase 2	DDAH2	1	8	8	3	4	4	1	1	2	3	3	1	3	4	4	1	1	2	3	3	1	3	4	4	1	1	2	3	3	1
Immunoglobulin kappa variable 1-8	IGKV1-8/-9	2	4	3	1	1	1	1	1	1	2	1	2	0	0	0	0	0	0	1	0	1	2	2	2	2	1	1	3	1	3
Protein CREG1	CREG1	1	1	1	0	1	1	1	1	0	1	1	1	0	1	1	1	1	0	1	1	1	0	1	1	2	2	0	3	2	2
Inosine triphosphate pyrophosphatase	ITPA	1	5	5	0	0	0	1	2	1	2	2	3	0	0	0	1	2	1	2	2	3	0	0	0	1	2	1	2	2	3
Beta-1,3-galactosyl-O-glycosyl-glycoprotein beta-1,6-N-acetylglucosaminyltransferase 3	GCNT3	1	6	6	0	0	0	1	2	2	2	3	2	0	0	0	1	2	2	2	3	2	0	0	0	1	2	2	2	3	2
Beta-galactoside alpha-2,6-sialyltransferase 1	ST6GAL1	1	6	6	4	0	0	3	1	1	1	3	3	0	0	0	3	1	1	1	3	3	0	0	0	3	1	1	1	3	3
Protein S100-A4	S100A4	1	7	7	4	3	3	2	2	2	2	2	3	4	3	3	2	2	2	2	2	3	6	3	4	2	2	2	2	2	3
UV excision repair protein RAD23 homolog B	RAD23B	1	7	7	4	3	2	2	2	2	3	2	2	4	3	2	2	2	2	3	2	2	4	3	2	2	2	2	3	2	2
Protein SEC13 homolog	SEC13	1	5	5	4	3	3	1	2	3	3	2	2	2	3	3	1	2	3	3	2	2	2	3	3	1	2	3	3	2	2
Cathepsin L1	CTSL	1	6	6	3	2	2	2	2	2	2	3	2	3	2	2	2	2	2	2	3	2	3	2	2	2	2	2	2	3	2
Bone morphogenetic protein receptor type-2	BMPR2	1	1	1	1	1	1	1	1	1	1	1	1	1	1	1	1	1	1	1	1	1	2	2	1	2	2	2	2	3	2
Coagulation factor XII	F12	1	7	7	4	6	5	2	3	2	1	3	3	4	6	5	2	3	2	1	3	3	4	6	5	2	3	2	1	3	3
Dermcidin	DCD	1	6	6	2	1	2	2	2	3	2	2	3	2	1	2	2	2	3	2	2	3	2	1	2	2	2	3	2	2	3
Hsc70-interacting protein	ST13;ST13P5/P4	3	9	9	6	5	5	3	2	3	3	2	2	6	5	5	3	2	3	3	2	2	6	5	6	3	2	3	3	2	2
Actin-related protein 2/3 complex subunit 2	ARPC2	1	12	12	3	4	1	2	3	3	2	1	3	3	4	1	2	3	3	2	1	3	3	4	2	2	3	3	3	1	3
Actin-related protein 2	ACTR2	1	11	11	3	2	3	3	2	3	1	2	4	3	2	3	3	2	3	1	2	4	3	2	3	3	2	3	1	2	4
Protein S100-A11	S100A11	1	8	8	4	2	4	3	3	3	2	3	2	4	2	4	3	3	3	2	3	2	7	4	6	3	3	3	2	3	2
Astrocytic phosphoprotein PEA-15	PEA15	1	4	4	4	3	2	2	2	2	2	2	2	3	3	2	2	2	2	2	2	2	4	4	3	3	3	3	2	2	3
Homeodomain-only protein	HOPX	1	2	2	2	2	2	2	2	2	2	2	2	2	2	2	2	2	2	2	2	2	3	3	4	3	3	3	3	2	2
14-3-3 protein gamma	YWHAG	1	12	9	4	5	5	5	4	4	4	3	4	2	3	4	3	3	3	3	2	2	2	3	4	3	3	3	3	2	2
Cathepsin Z	CTSZ	1	7	7	2	3	2	3	3	3	2	2	3	2	3	2	3	3	3	2	2	3	2	3	2	3	3	3	2	2	3
40S ribosomal protein S12	RPS12	1	7	7	4	3	3	2	3	2	1	2	4	2	3	3	2	3	2	1	2	4	2	3	3	3	4	3	1	2	4
Rho-related GTP-binding protein RhoC	RHOC	2	7	1	1	1	1	3	4	3	2	3	1	0	0	0	0	0	0	0	0	0	1	1	1	3	4	3	2	4	1
Elongation factor 1-beta	EEF1B2	1	5	5	3	4	4	3	4	5	3	2	2	3	4	4	3	4	5	3	2	2	3	4	4	3	4	5	3	2	2
Pigment epithelium-derived factor	SERPINF1	2	15	15	7	8	6	5	3	5	3	2	2	7	8	6	5	3	5	3	2	2	7	9	6	5	3	5	3	2	2
Proliferation-associated protein 2G4	PA2G4	1	14	14	4	7	11	6	6	5	3	1	3	8	7	11	6	6	5	3	1	3	8	7	11	6	6	5	3	1	3
Cornifin-B	SPRR1B	1	6	4	4	3	2	4	4	5	2	1	2	1	2	1	3	3	3	1	0	1	2	3	4	5	6	7	3	2	2
Desmoglein-1	DSG1	1	11	11	0	0	0	8	8	11	3	2	2	0	0	0	8	8	11	3	2	2	0	0	0	10	10	14	3	2	2
Galectin-7	LGALS7	1	12	12	0	0	0	10	12	11	2	3	2	0	0	0	10	12	11	2	3	2	0	0	0	16	16	14	2	3	2
Myeloperoxidase	MPO	2	22	22	12	11	13	0	0	0	2	2	2	12	11	13	0	0	0	2	2	2	12	11	13	0	0	0	2	2	2
Proteasome subunit beta type-9	PSMB9	1	6	6	4	5	5	0	0	0	2	1	2	5	5	5	0	0	0	2	1	2	6	6	6	0	0	0	2	2	2
Angiotensinogen	AGT	1	7	7	5	5	4	0	0	0	2	2	2	5	5	4	0	0	0	2	2	2	5	5	4	0	0	0	2	2	2
NAD(P)H dehydrogenase [quinone] 1	NQO1	1	9	9	4	4	4	0	0	0	2	2	2	5	4	4	0	0	0	2	2	2	6	4	4	0	0	0	2	2	2
Solute carrier family 12 member 2	SLC12A2	1	12	12	4	2	2	0	0	0	2	2	2	5	2	2	0	0	0	2	2	2	5	2	2	0	0	0	2	2	2
Lumican	LUM	1	4	4	1	2	2	0	0	0	2	2	2	1	2	2	0	0	0	2	2	2	1	2	2	0	0	0	2	2	2
Casein kinase I isoform gamma-3	CSNK1G3	1	1	1	1	0	1	0	0	0	1	1	1	1	0	1	0	0	0	1	1	1	2	0	2	0	0	0	2	2	2
Cornulin	CRNN	1	3	3	0	0	0	0	0	0	2	3	1	0	0	0	0	0	0	2	3	1	0	0	0	0	0	0	2	3	1
Mannosyl-oligosaccharide 1,2-alpha-mannosidase IA	MAN1A1	1	2	2	0	0	0	0	0	0	2	2	2	0	0	0	0	0	0	2	2	2	0	0	0	0	0	0	2	2	2
Proteasome subunit beta type-4	PSMB4	1	8	8	4	5	5	0	1	0	3	2	1	6	5	5	0	1	0	3	2	1	8	6	5	0	1	0	3	2	1
Aldo-keto reductase family 1 member C2	AKR1C2	1	21	4	16	11	13	2	3	2	6	6	5	3	3	3	0	1	0	2	2	2	4	4	5	0	1	0	2	2	2
Trefoil factor 1	TFF1	1	4	4	4	3	2	1	0	0	2	2	2	3	3	2	1	0	0	2	2	2	3	3	3	1	0	0	2	2	2

Insulin-like growth factor-binding protein 6	IGFBP6	1	2	2	2	1	2	1	0	0	2	2	2	2	1	2	1	0	0	2	2	2	2	1	2	1	0	0	2	2	2
26S proteasome non-ATPase regulatory subunit 10	PSMD10	1	6	6	4	1	1	1	0	0	2	2	2	3	1	1	1	0	0	2	2	2	3	1	1	1	0	0	2	2	2
Syntenin-1	SDCBP	1	6	6	4	1	1	0	0	1	3	1	2	1	1	1	0	0	1	3	1	2	1	1	1	0	0	1	3	1	2
Bifunctional ATP-dependent dihydroxyacetone kinase/FAD-AMP lyase (cyclizing)	DAK	1	10	10	1	0	1	0	0	1	2	1	2	1	0	1	0	0	1	2	1	2	1	0	1	0	0	1	2	1	3
C-X-C motif chemokine 10	CXCL10	1	2	2	0	0	0	1	0	0	2	2	2	0	0	0	1	0	0	2	2	2	0	0	0	1	0	0	2	2	2
Thiosulfate sulfurtransferase/rhodanese-like domain-containing protein 1	TSTD1	1	6	6	4	5	3	1	1	0	2	2	2	6	5	3	1	1	0	2	2	2	6	5	3	1	1	0	2	2	2
LIM and SH3 domain protein 1	LASP1	1	7	7	5	3	2	1	0	0	2	2	2	5	3	2	1	0	0	2	2	2	5	3	2	2	0	0	2	2	2
Cellular nucleic acid-binding protein	CNBP	1	3	3	3	3	3	1	1	0	2	2	2	3	3	3	1	1	0	2	2	2	3	3	3	1	1	0	2	2	2
Flavin reductase (NADPH)	BLVRB	1	10	10	7	6	7	2	1	0	2	2	2	7	6	7	2	1	0	2	2	2	10	12	10	2	1	0	2	2	2
Low molecular weight phosphotyrosine protein phosphatase	ACP1	1	8	8	6	6	6	1	1	1	1	2	3	6	6	6	1	1	1	1	2	3	6	6	6	1	1	1	1	2	3
Rab GDP dissociation inhibitor alpha	GDI1	1	15	7	7	9	6	2	2	1	5	5	5	4	6	4	1	1	1	2	2	2	5	7	5	1	1	1	2	2	2
Tropomodulin-3	TMOD3	1	8	8	4	4	4	1	1	1	2	2	2	3	4	4	1	1	1	2	2	2	3	4	4	1	1	1	2	2	2
Tubulin polymerization-promoting protein family member 3	TPPP3	1	4	4	4	2	1	2	1	0	2	2	2	4	2	1	2	1	0	2	2	2	4	2	1	2	1	0	2	2	2
Ig heavy chain V-I region V35	IGHV1-2	1	4	3	1	2	2	2	1	0	2	1	3	1	1	1	1	1	0	2	1	2	1	2	2	2	1	0	2	1	3
Sulfatase-modifying factor 2	SUMF2	1	4	4	4	2	1	1	1	1	2	2	2	2	2	1	1	1	1	2	2	2	2	2	1	1	1	1	2	2	2
Nuclease-sensitive element-binding protein 1	YBX1	2	6	3	3	1	2	1	1	2	2	2	2	2	1	1	1	1	1	2	2	2	2	1	1	1	1	1	2	2	2
Ig heavy chain V-I region EU	IGHV1-69-2	2	3	2	1	1	0	1	1	1	2	2	2	0	0	0	0	0	0	1	1	1	1	1	0	1	1	1	2	2	2
E3 ubiquitin-protein ligase RNF216	RNF216	1	1	1	4	1	1	1	0	1	1	1	1	0	1	1	1	0	1	1	1	1	0	1	1	2	0	1	2	2	2
Immunoglobulin kappa variable 6-21	IGKV6-21	1	2	1	0	0	0	1	1	1	2	2	2	0	0	0	1	1	1	1	1	1	0	0	0	1	1	1	2	2	2
Obg-like ATPase 1	OLA1	1	8	8	4	0	0	1	1	1	2	2	2	0	0	0	1	1	1	2	2	2	0	0	0	1	1	1	2	2	2
CMP-N-acetylneuraminate-beta-galactosamide-alpha-2,3-sialyltransferase 1	ST3GAL1	1	3	3	4	0	0	1	2	0	2	2	1	0	0	0	1	2	0	2	2	1	0	0	0	1	2	0	2	2	2
Prostate stem cell antigen	PSCA	1	3	3	4	3	2	2	1	1	2	2	2	3	3	2	2	1	1	2	2	2	4	3	2	2	1	1	2	2	2
Macrophage migration inhibitory factor	MIF	1	4	4	3	2	2	2	1	1	2	2	2	3	2	2	2	1	1	2	2	2	3	2	2	2	1	1	2	2	2
Pro-cathepsin H	CTSH	1	6	6	2	1	1	0	2	2	3	1	2	2	1	1	0	2	2	3	1	2	2	1	1	0	2	2	3	1	2
Metalloproteinase inhibitor 2	TIMP2	1	8	8	4	0	2	1	1	2	2	2	1	1	0	2	1	1	2	2	2	1	1	0	2	1	1	2	3	2	1
Alpha-galactosidase A	GLA	1	4	4	0	0	0	1	1	2	3	1	2	0	0	0	1	1	2	3	1	2	0	0	0	1	1	2	3	1	2
Actin, alpha cardiac muscle 1	ACTC1/A1/A2/G2	4	19	4	9	7	7	9	7	7	7	8	6	1	1	1	1	1	1	1	1	1	3	3	2	1	1	3	2	2	2
Immunoglobulin kappa variable 2-24	IGKV2-24;IGKV2D-24	2	3	3	1	2	1	2	1	2	2	2	2	1	2	1	2	1	2	2	2	2	1	2	1	2	1	2	2	2	2
Small proline-rich protein 3	SPRR3	1	6	6	4	1	1	2	1	2	2	1	3	2	1	1	2	1	2	2	1	3	2	1	1	2	1	2	2	1	3
Multifunctional protein ADE2	PAICS	1	8	8	4	0	2	3	1	1	2	2	2	0	0	2	3	1	1	2	2	2	0	0	2	3	1	1	2	2	2
Protein FAM3B	FAM3B	1	6	6	0	1	0	2	2	1	2	2	2	0	1	0	2	2	1	2	2	2	0	1	0	2	2	1	2	2	2
Ubiquitin-conjugating enzyme E2 N	UBE2N	2	8	8	0	0	0	2	2	1	2	1	3	0	0	0	2	2	1	2	1	3	0	0	0	2	2	1	2	1	3
Glutaminyl-peptide cyclotransferase	QPCT	1	5	5	4	0	0	1	2	2	2	2	2	0	0	0	1	2	2	2	2	2	0	0	0	1	2	1	2	2	2
Tubulin-specific chaperone A	TBCA	1	6	6	4	5	4	3	2	1	1	2	3	4	5	4	3	2	1	1	2	3	6	6	6	3	2	1	1	2	3
Tumor protein D52	TPD52	1	7	7	4	7	4	2	2	2	2	2	2	5	7	4	2	2	2	2	2	2	5	7	4	2	2	2	2	2	2
Actin, cytoplasmic 1	ACTB	5	31	1	17	15	17	16	15	14	16	18	17	1	1	1	1	1	1	1	1	1	2	2	2	2	2	2	2	2	2
Ig kappa chain V-II region FR	IGKV2D-28;IGKV2-40	4	5	3	2	2	2	2	2	2	2	2	2	1	1	1	1	1	1	1	1	1	2	2	2	2	2	2	2	2	2
Immunoglobulin heavy variable 5-51	IGHV5-51	2	4	4	1	2	2	2	2	2	3	1	2	1	2	2	2	2	2	3	1	2	1	2	2	2	2	2	3	1	2
Ig lambda chain V-I region HA	IGKV1-47	2	3	3	1	2	2	2	2	2	2	2	2	1	2	2	2	2	2	2	2	2	1	2	2	2	2	2	2	2	2
Collagen alpha-1(XIV) chain	COL14A1	1	2	2	2	1	1	2	2	2	2	2	2	2	1	1	2	2	2	2	2	2	2	1	1	2	2	2	2	2	2
Folate receptor alpha	FOLR1	1	4	4	1	0	0	2	2	2	2	2	2	1	0	0	2	2	2	2	2	2	1	0	0	2	2	2	2	2	2
Alpha-actinin-1	ACTN1	1	30	14	4	5	3	12	13	9	9	8	10	0	0	0	2	2	2	2	2	2	0	0	0	2	2	2	2	2	2
Histidine-rich glycoprotein	HRG	1	15	15	9	8	6	2	2	2	1	1	1	9	8	6	2	2	2	1	1	1	10	9	7	3	2	2	2	2	2
Major vault protein	MVP	1	34	34	4	6	7	2	3	2	2	2	2	9	6	7	2	3	2	2	2	2	10	6	8	2	3	2	2	2	2
6-phosphogluconolactonase	PGLS	2	12	12	6	3	5	3	1	3	3	1	2	6	3	5	3	1	3	3	1	2	6	3	5	3	1	3	3	1	2
Microtubule-associated protein RP/EB family member 1	MAPRE1	1	10	10	4	2	3	3	3	2	2	2	2	4	2	3	3	3	2	2	2	2	4	2	3	3	3	2	2	2	2
N-acetyl-D-glucosamine kinase	NAGK	1	10	10	4	6	6	5	3	1	2	2	2	8	6	6	5	3	1	2	2	2	9	6	6	5	3	1	2	2	2
Sciellin	SCEL	1	15	15	4	6	3	4	3	2	2	2	2	5	6	3	4	3	2	2	2	2	5	6	3	4	3	2	2	2	2
Lysosomal alpha-mannosidase	MAN2B1	1	5	5	3	2	2	4	2	3	1	3	2	3	2	2	4	2	3	1	3	2	3	2	2	4	2	3	1	3	2
D-3-phosphoglycerate dehydrogenase	PHGDH	1	9	9	4	0	0	4	2	3	2	2	2	0	0	0	4	2	3	2	2	2	0	0	0	4	2	3	2	2	2

Translationally-controlled tumor protein	TPT1	3	7	7	4	3	4	3	2	2	2	2	2	4	3	4	3	2	2	2	2	2	4	3	4	4	3	3	2	2	2
T-complex protein 1 subunit zeta	CCT6A	1	15	14	0	0	0	2	4	4	2	2	2	0	0	0	2	3	3	2	2	2	0	0	0	2	4	4	2	2	2
Desmocollin-2	DSC2	1	6	6	1	0	1	3	4	4	3	2	1	1	0	1	3	4	4	3	2	1	1	0	1	3	4	4	3	2	1
Beta-hexosaminidase subunit alpha	HEXA	1	6	6	2	3	2	2	6	4	3	1	2	2	3	2	2	6	4	3	1	2	2	3	2	2	6	4	3	1	2
Prostatic acid phosphatase	ACPP	1	4	4	1	1	1	4	4	3	2	2	2	1	1	1	4	4	3	2	2	2	1	1	1	5	5	3	2	2	2
Cathepsin S	CTSS	1	5	5	2	4	4	5	5	4	1	2	3	2	4	4	5	5	4	1	2	3	3	4	4	5	5	4	1	2	3
Gamma-glutamylcyclotransferase	GGCT	1	10	10	3	2	1	6	4	4	1	3	2	3	2	1	6	4	4	1	3	2	3	2	1	6	4	4	1	3	2
Carboxypeptidase D	CPD	1	7	7	0	0	0	3	5	4	3	1	2	0	0	0	3	5	4	3	1	2	0	0	0	3	6	5	3	1	2
Elongation factor 1-delta	EEF1D	1	11	11	6	3	2	5	6	6	1	2	3	6	3	2	5	6	6	1	2	3	6	3	2	5	6	6	1	2	3
Fibrinogen alpha chain	FGA	2	20	20	3	2	2	6	4	6	2	1	3	3	2	2	6	4	6	2	1	3	3	2	2	6	4	7	2	1	3
Dihydropyrimidine dehydrogenase [NADP(+)]	DPYD	1	18	18	11	9	10	0	0	0	2	2	1	11	9	10	0	0	0	2	2	1	13	10	10	0	0	0	2	2	1
Chloride intracellular channel protein 6	CLIC6	2	9	9	5	6	5	0	0	0	1	2	2	5	6	5	0	0	0	1	2	2	6	7	6	0	0	0	1	2	2
Secernin-1	SCRN1	1	8	7	4	3	5	0	0	0	3	1	1	3	3	5	0	0	0	3	1	1	4	5	6	0	0	0	3	1	1
Phospholysine phosphohistidine inorganic pyrophosphate phosphatase	LHPP	1	5	5	3	2	3	0	0	0	1	2	2	3	2	3	0	0	0	1	2	2	4	2	4	0	0	0	1	2	2
Hemoglobin subunit alpha	HBA1	2	3	3	2	3	2	0	0	0	1	1	1	2	3	2	0	0	0	1	1	1	2	3	2	0	0	0	2	2	1
Glutathione S-transferase omega-1	GSTO1	1	8	8	3	2	2	0	0	0	2	2	1	3	2	2	0	0	0	2	2	1	3	2	2	0	0	0	2	2	1
Putative adenosylhomocysteinase 3	AHCYL2	1	9	5	1	3	2	0	0	0	2	2	1	0	0	0	0	0	0	0	0	0	1	3	2	0	0	0	2	2	1
ADP-ribosylation factor-like protein 3	ARL3	1	7	7	2	2	1	0	0	0	1	2	2	2	2	1	0	0	0	1	2	2	2	2	1	0	0	0	1	2	2
Inositol monophosphatase 1	IMPA1	1	8	8	0	0	0	0	0	0	1	2	2	0	0	0	0	0	0	1	2	2	0	0	0	0	0	0	1	2	2
Testican-3	SPOCK3	1	3	3	4	0	0	0	0	0	2	1	2	0	0	0	0	0	0	2	1	2	0	0	0	0	0	0	2	1	2
Xaa-Pro dipeptidase	PEPD	1	12	12	4	9	8	1	0	0	2	1	2	8	9	8	1	0	0	2	1	2	9	11	9	1	0	0	2	1	2
Breast carcinoma-amplified sequence 1	BCAS1	1	10	10	6	7	6	1	0	0	2	1	2	6	7	6	1	0	0	2	1	2	6	7	6	1	0	0	2	1	2
Cytoplasmic aconitate hydratase	ACO1	1	12	12	6	5	5	1	0	0	2	2	1	6	5	5	1	0	0	2	2	1	6	5	5	1	0	0	2	2	1
Complement factor D	CFD	1	7	7	3	1	1	1	0	0	1	2	2	3	1	1	1	0	0	1	2	2	3	1	1	1	0	0	1	2	2
Sialate O-acetylesterase	SIAE	1	4	4	4	0	0	1	0	0	2	1	2	2	0	0	1	0	0	2	1	2	2	0	0	1	0	0	2	1	2
Ras-related protein Rab-11B	RAB11B/A	2	6	6	0	0	1	1	0	0	1	2	2	0	0	1	1	0	0	1	2	2	0	0	1	1	0	0	1	2	2
C-1-tetrahydrofolate synthase, cytoplasmic	MTHFD1	1	21	20	0	0	0	0	0	1	2	2	1	0	0	0	0	0	1	2	2	1	0	0	0	0	0	1	2	2	1
Toll-like receptor 2	TLR2	1	2	2	4	0	0	0	1	0	2	1	2	0	0	0	0	1	0	2	1	2	0	0	0	0	1	0	2	1	2
Proteasome subunit alpha type-4	PSMA4	1	9	9	4	6	7	0	1	1	3	1	1	8	6	7	0	1	1	3	1	1	8	6	8	0	1	1	3	1	1
Copper transport protein ATOX1	ATOX1	1	5	5	2	2	3	1	1	0	2	1	2	2	2	3	1	1	0	2	1	2	2	2	3	1	1	0	2	1	2
Immunoglobulin lambda variable 8-61	IGLV8-61	1	1	1	1	1	1	1	0	1	1	1	1	1	1	1	1	0	1	1	1	1	1	1	1	1	0	1	1	2	2
Asparagine--tRNA ligase, cytoplasmic	NARS	1	10	10	4	3	3	1	1	1	2	1	2	3	3	3	1	1	1	2	1	2	3	3	3	1	1	1	2	1	2
Prothymosin alpha	PTMA	1	2	2	4	2	2	1	1	1	2	2	1	2	2	2	1	1	1	2	2	1	2	2	2	1	1	1	2	2	1
14-3-3 protein eta	YWHAH	1	9	7	4	2	2	3	2	2	4	1	3	2	1	1	1	1	1	3	0	2	2	1	1	1	1	1	3	0	2
Ig kappa chain V-III region VG	IGKV3D-11	2	4	4	1	1	1	1	1	1	3	1	1	1	1	1	1	1	1	3	1	1	1	1	1	1	1	1	3	1	1
Complement C1r subcomponent-like protein	C1RL	1	3	3	1	1	1	1	1	1	1	2	2	1	1	1	1	1	1	1	2	2	1	1	1	1	1	1	1	2	2
Lysine--tRNA ligase	KARS	1	8	8	1	1	1	1	1	1	2	2	1	1	1	1	1	1	1	2	2	1	1	1	1	1	1	1	2	2	1
Alpha-N-acetylgalactosaminidase	NAGA	1	4	4	4	1	1	1	1	1	2	1	2	1	1	1	1	1	1	2	1	2	1	1	1	1	1	1	2	1	2
V-type proton ATPase subunit S1	ATP6AP1	1	2	2	0	0	0	1	1	1	2	1	2	0	0	0	1	1	1	2	1	2	0	0	0	1	1	1	2	1	2
Ubiquitin-conjugating enzyme E2 variant 1	UBE2V1	1	6	3	4	0	0	1	1	1	2	2	1	0	0	0	1	1	1	1	1	1	0	0	0	1	1	1	2	2	1
S-formylglutathione hydrolase	ESD	1	10	10	5	6	6	2	1	1	2	2	1	5	6	6	2	1	1	2	2	1	7	8	8	2	1	1	2	2	1
Immunoglobulin heavy variable 3-74	IGHV3-74	3	5	1	2	3	3	2	2	2	3	3	2	0	1	0	0	1	0	1	1	0	1	2	1	1	2	1	2	2	1
Epidermal growth factor receptor kinase substrate 8-like protein 2	EPS8L2	1	10	10	1	1	1	2	1	1	1	2	2	1	1	1	2	1	1	1	2	2	1	1	1	2	1	1	1	2	2
Ig lambda chain V-III region LOI	IGKV3-21	1	3	2	4	1	1	1	1	1	2	2	1	0	0	0	0	0	0	1	1	1	1	1	1	1	1	2	2	2	1
Alanine--tRNA ligase, cytoplasmic	AARS	1	8	8	0	1	1	1	2	1	2	2	1	0	1	1	1	2	1	2	2	1	0	1	1	1	2	1	2	2	1
Multiple inositol polyphosphate phosphatase 1	MINPP1	1	5	5	0	0	0	1	2	1	2	2	1	0	0	0	1	2	1	2	2	1	0	0	0	1	2	1	2	2	1
Destrin	DSTN	1	10	10	5	3	4	2	2	1	2	1	2	5	3	4	2	2	1	2	1	2	6	4	5	2	2	1	2	1	2
Kallikrein-11	KLK11	1	5	5	3	4	4	1	2	2	1	2	2	3	4	4	1	2	2	1	2	2	3	4	4	1	2	2	1	2	2
Prostaglandin E synthase 3	PTGES3	1	5	5	4	3	3	1	2	2	2	2	1	3	3	3	1	2	2	2	2	1	3	3	4	1	2	2	2	2	1
Aflatoxin B1 aldehyde reductase member 2	AKR7A2	3	7	7	2	2	2	2	2	1	2	1	2	2	2	2	2	2	1	2	1	2	2	2	2	2	2	1	2	1	2

Q6P531	Gamma-glutamyltransferase 6	GGT6	1	4	4	0	0	0	3	0	2	2	2	1	0	0	0	3	0	2	2	2	1	0	0	0	3	0	2	2	2	1
P48723	Heat shock 70 kDa protein 13	HSPA13	1	6	6	0	0	0	2	2	1	2	1	2	0	0	0	2	2	1	2	1	2	0	0	0	2	2	1	2	1	2
P13796	Plastin-2	LCP1	1	21	17	0	0	0	4	3	5	5	3	5	0	0	0	2	1	2	2	1	2	0	0	0	2	1	2	2	1	2
Q6PCB0	von Willebrand factor A domain-containing protein 1	VWA1	1	3	3	4	0	0	2	2	1	1	2	2	0	0	0	2	2	1	1	2	2	0	0	0	2	2	1	1	2	2
Q14914	Prostaglandin reductase 1	PTGR1	1	8	8	4	1	2	2	1	3	2	2	1	1	1	2	2	1	3	2	2	1	1	1	2	2	1	3	2	2	1
Q9BWS9	Chitinase domain-containing protein 1	CHID1	1	5	5	1	1	1	3	1	2	1	3	1	1	1	1	3	1	2	1	3	1	1	1	1	3	1	2	1	3	1
P39023	60S ribosomal protein L3	RPL3	2	6	6	5	3	5	2	2	3	2	1	2	5	3	5	2	2	3	2	1	2	5	3	5	2	2	3	2	1	2
Q00688	Peptidyl-prolyl cis-trans isomerase FKBP3	FKBP3	1	6	6	2	4	2	2	2	2	1	2	2	2	4	2	2	2	2	1	2	2	3	5	3	3	2	2	1	2	2
Q99538	Legumain	LGMN	1	10	10	0	0	0	3	2	3	2	2	1	0	0	0	3	2	3	2	2	1	0	0	0	3	2	3	2	2	1
P13667	Protein disulfide-isomerase A4	PDIA4	1	14	14	4	2	3	3	3	3	1	2	2	5	2	3	3	3	3	1	2	2	5	2	3	3	3	3	1	2	2
P39019	40S ribosomal protein S19	RPS19	1	5	5	4	1	1	3	3	3	1	2	2	2	1	1	3	3	3	1	2	2	2	1	1	3	3	3	1	2	2
P68366	Tubulin alpha-4A chain	TUBA4A	1	19	5	4	6	5	12	13	11	9	7	9	1	1	1	3	3	3	2	1	2	1	1	1	3	3	3	2	1	2
Q14204	Cytoplasmic dynein 1 heavy chain 1	DYNC1H1	1	21	21	0	0	0	5	4	3	2	2	1	0	0	0	5	4	3	2	2	1	0	0	0	5	4	3	2	2	1
Q9HBR0	Putative sodium-coupled neutral amino acid transporter 10	SLC38A10	1	8	8	4	1	0	6	3	6	2	2	1	0	1	0	6	3	6	2	2	1	0	1	0	6	3	6	2	2	1
Q96G03	Phosphoglucomutase-2	PGM2	1	16	16	4	0	0	5	3	7	3	1	1	0	0	0	5	3	7	3	1	1	0	0	0	5	3	7	3	1	1
P01011	Alpha-1-antichymotrypsin	SERPINA3	1	15	15	4	5	5	6	6	5	1	3	1	5	5	5	6	6	5	1	3	1	5	5	5	6	7	5	1	3	1
Q9BUT1	3-hydroxybutyrate dehydrogenase type 2	BDH2	1	6	6	4	5	4	0	0	0	1	1	2	4	5	4	0	0	0	1	1	2	5	6	5	0	0	0	1	1	2
P04899	Guanine nucleotide-binding protein G(i) subunit alpha-2	GNAI2	5	15	11	5	5	5	0	0	0	1	1	2	4	3	3	0	0	0	1	1	1	5	5	5	0	0	0	1	1	2
Q9P2T1	GMP reductase 2	GMPR2	1	6	5	5	4	4	0	0	0	1	1	2	4	3	3	0	0	0	0	0	2	6	4	4	0	0	0	1	1	2
Q6GMV3	Putative peptidyl-tRNA hydrolase PTRHD1	PTRHD1	1	3	3	4	3	2	0	0	0	2	1	1	3	3	2	0	0	0	2	1	1	3	3	2	0	0	0	2	1	1
Q14376	UDP-glucose 4-epimerase	GALE	1	9	9	2	2	3	0	0	0	1	1	2	2	2	3	0	0	0	1	1	2	2	2	3	0	0	0	1	1	2
Q9UGI8	Testin	TES	1	4	4	4	3	2	0	0	0	1	2	1	2	3	2	0	0	0	1	2	1	2	3	2	0	0	0	1	2	1
Q03591	Complement factor H-related protein 1	CFHR1	2	7	2	3	3	3	0	0	1	2	1	1	1	1	1	0	0	0	1	1	1	1	1	1	0	0	0	2	1	1
Q15631	Translin	TSN	1	5	5	4	2	0	0	0	0	1	2	1	1	2	0	0	0	0	1	2	1	1	2	0	0	0	0	1	2	1
P22102	Trifunctional purine biosynthetic protein adenosine-3	GART	1	8	8	1	1	0	0	0	0	1	1	2	1	1	0	0	0	0	1	1	2	1	1	0	0	0	0	1	1	2
P49902	Cytosolic purine 5-nucleotidase	NT5C2	1	5	5	4	0	1	0	0	0	2	2	0	1	0	1	0	0	0	2	2	0	1	0	1	0	0	0	2	2	0
Q13444	Disintegrin and metalloproteinase domain-containing protein 15	ADAM15	1	3	3	0	0	0	0	0	0	2	1	1	0	0	0	0	0	0	2	1	1	0	0	0	0	0	0	2	1	1
P55957	BH3-interacting domain death agonist	BID	1	4	4	0	0	0	0	0	0	1	2	1	0	0	0	0	0	0	1	2	1	0	0	0	0	0	0	1	2	1
Q5VW32	BRO1 domain-containing protein BROX	BROX	1	4	4	0	0	0	0	0	0	2	0	2	0	0	0	0	0	0	2	0	2	0	0	0	0	0	0	2	0	2
Q9BTY2	Plasma alpha-L-fucosidase	FUCA2	1	2	2	0	0	0	0	0	0	1	2	1	0	0	0	0	0	0	1	2	1	0	0	0	0	0	0	1	2	1
Q8WWY8	Lipase member H	LIPH	1	2	2	0	0	0	0	0	0	1	2	1	0	0	0	0	0	0	1	2	1	0	0	0	0	0	0	1	2	1
P09237	Matrilysin	MMP7	1	5	5	0	0	0	0	0	0	1	2	1	0	0	0	0	0	0	1	2	1	0	0	0	0	0	0	1	2	1
Q16401	26S proteasome non-ATPase regulatory subunit 5	PSMD5	1	5	5	4	0	0	0	0	0	2	1	1	0	0	0	0	0	0	2	1	1	0	0	0	0	0	0	2	1	1
Q9H173	Nucleotide exchange factor SIL1	SIL1	1	4	4	4	0	0	0	0	0	2	2	0	0	0	0	0	0	0	2	2	0	0	0	0	0	0	0	2	2	0
P68402	Platelet-activating factor acetylhydrolase IB subunit beta	PAFAH1B2	1	5	5	4	3	3	0	1	0	1	1	1	1	3	3	0	1	0	1	1	1	1	3	3	0	1	0	1	2	1
Q9BRF8	Serine/threonine-protein phosphatase CPPED1	CPPED1	1	7	7	4	3	5	0	1	1	1	1	2	4	3	5	0	1	1	1	1	2	4	3	5	0	1	1	1	1	2
Q10567	AP-1 complex subunit beta-1	AP1B1	1	12	6	2	2	2	1	0	1	1	1	2	1	1	1	1	0	1	0	0	1	2	2	2	1	0	1	1	1	2
P61960	Ubiquitin-fold modifier 1	UFM1	1	2	2	4	1	0	0	1	1	1	2	1	1	1	0	0	1	1	1	2	1	1	1	0	0	1	1	1	2	1
P48643	T-complex protein 1 subunit epsilon	CCT5	1	15	15	0	0	0	1	1	0	1	1	2	0	0	0	1	1	0	1	1	2	0	0	0	1	1	0	1	1	2
P49721	Proteasome subunit beta type-2	PSMB2	1	9	9	4	3	4	1	1	1	2	1	1	4	3	4	1	1	1	2	1	1	4	3	4	1	1	1	2	1	1
O14579	Coatomer subunit epsilon	COPE	1	7	7	1	2	1	1	1	1	2	1	1	1	2	1	1	1	1	2	1	1	1	2	1	1	1	1	2	1	1
Q9NQX5	Neural proliferation differentiation and control protein 1	NPDC1	1	2	2	4	1	1	1	1	1	2	1	1	1	1	1	1	1	1	2	1	1	1	1	1	1	1	1	2	1	1
Q9NZ08	Endoplasmic reticulum aminopeptidase 1	ERAP1	1	5	5	0	1	0	1	1	1	1	2	1	0	1	0	1	1	1	1	2	1	0	1	0	1	1	1	1	2	1
Q86SQ4	G-protein coupled receptor 126	GPR126	1	4	4	0	0	0	1	1	1	1	2	1	0	0	0	1	1	1	1	2	1	0	0	0	1	1	1	1	2	1
P15289	Arylsulfatase A	ARSA	1	4	4	2	0	1	1	2	1	1	2	1	2	0	1	1	2	1	1	2	1	2	0	1	1	2	1	1	2	1
O95965	Integrin beta-like protein 1	ITGBL1	1	3	3	1	1	0	1	2	1	2	1	1	1	1	0	1	2	1	2	1	1	1	1	0	1	2	1	2	1	1
P26599	Polypyrimidine tract-binding protein 1	PTBP1	1	7	7	4	0	0	1	1	2	2	0	2	0	0	0	1	1	2	2	0	2	0	0	0	1	1	2	2	0	2
Q9UKK9	ADP-sugar pyrophosphatase	NUDT5	1	6	6	4	2	4	1	2	2	1	1	2	4	2	4	1	2	2	1	1	2	4	2	4	1	2	2	1	1	2
P63208	S-phase kinase-associated protein 1	SKP1	1	7	7	4	4	3	2	1	2	2	2	0	3	4	3	2	1	2	2	2	0	3	4	3	2	1	2	2	2	0
A0A0B4J1V0	Immunoglobulin heavy variable 3-15	IGHV3-15	1	6	3	4	2	2	2	1	2	1	3	2	3	2	2	2	1	2	1	2	1	3	2	2	2	1	2	1	2	1

P06310	Ig kappa chain V-II region RPMI 6410	IGKV2D-30	2	5	3	2	1	1	3	2	3	2	2	3	1	0	0	2	1	2	1	1	2	1	0	0	2	1	2	1	1	2
P49368	T-complex protein 1 subunit gamma	CCT3	1	17	17	0	1	0	1	1	3	2	1	1	0	1	0	1	1	3	2	1	1	0	1	0	1	1	3	2	1	1
Q06828	Fibromodulin	FMOD	1	4	4	0	0	0	1	1	3	1	1	1	0	0	0	1	1	3	1	1	1	0	0	0	1	1	3	2	1	1
Q86UD1	Out at first protein homolog	OAF	1	4	4	4	0	0	2	2	1	1	1	2	0	0	0	2	2	1	1	1	2	0	0	0	2	2	1	1	1	2
P16403	Histone H1.2	HIST1H1C	1	9	3	6	3	6	5	6	7	4	3	4	2	2	2	2	2	2	1	1	2	2	2	2	2	2	2	1	1	2
O75348	V-type proton ATPase subunit G 1	ATP6V1G1	2	4	4	1	2	2	2	2	2	1	1	2	1	2	2	2	2	2	1	1	2	1	2	2	2	2	2	1	1	2
P63220	40S ribosomal protein S21	RPS21	1	4	4	4	1	2	2	2	2	1	1	2	2	1	2	2	2	2	1	1	2	2	1	2	2	2	2	1	1	2
P46777	60S ribosomal protein L5	RPL5	1	10	10	4	6	5	3	2	2	2	0	2	6	6	5	3	2	2	2	0	2	6	7	5	3	2	2	2	0	2
Q9UHL4	Dipeptidyl peptidase 2	DPP7	1	8	8	2	2	3	3	2	3	0	2	2	2	2	3	3	2	3	0	2	2	3	2	3	3	2	3	0	2	2
P60953	Cell division control protein 42 homolog	CDC42	1	6	5	0	0	0	3	0	5	2	1	1	0	0	0	2	0	4	2	1	0	0	0	0	3	0	5	2	1	1
P62906	60S ribosomal protein L10a	RPL10A	1	7	7	4	4	5	2	2	2	1	0	1	2	4	5	2	2	2	1	0	1	3	5	6	3	3	3	2	0	2
E9PAV3	Nascent polypeptide-associated complex subunit alpha, muscle-specific form	NACA	2	3	3	3	3	3	3	3	3	2	1	1	3	3	3	3	3	3	2	1	1	3	3	3	3	3	3	2	1	1
P62979	Ubiquitin-40S ribosomal protein S27a	RPS27A;UBA52;UBB;UBC	5	4	4	4	4	4	3	3	3	2	1	1	4	4	4	3	3	3	2	1	1	4	4	4	3	4	3	2	1	1
P55036	26S proteasome non-ATPase regulatory subunit 4	PSMD4	2	6	6	3	3	4	4	1	4	1	2	1	3	3	4	4	1	4	1	2	1	3	3	4	4	1	5	1	2	1
P05386	60S acidic ribosomal protein P1	RPLP1	1	5	3	4	2	2	4	2	3	2	1	1	2	2	2	3	2	2	2	1	1	2	2	2	4	3	3	2	1	1
P21281	V-type proton ATPase subunit B, brain isoform	ATP6V1B2	2	13	13	1	1	2	3	3	3	2	2	0	1	1	2	3	3	3	2	2	0	1	1	2	4	3	3	2	2	0
O75368	SH3 domain-binding glutamic acid-rich-like protein	SH3BGRL	1	10	10	4	7	7	3	4	4	2	1	1	6	7	7	3	4	4	2	1	1	7	9	8	3	4	4	2	1	1
P62424	60S ribosomal protein L7a	RPL7A	1	11	11	4	8	6	3	5	4	2	1	1	8	8	6	3	5	4	2	1	1	9	8	6	3	5	4	2	1	1
P33176	Kinesin-1 heavy chain	KIF5B	3	21	21	0	0	0	3	5	4	2	1	1	0	0	0	3	5	4	2	1	1	0	0	0	3	5	4	2	1	1
P00747	Plasminogen	PLG	3	31	31	10	11	11	4	5	4	1	1	2	10	11	11	4	5	4	1	1	2	10	11	11	4	5	4	1	1	2
P61247	40S ribosomal protein S3a	RPS3A	1	14	14	4	6	4	6	6	6	1	2	1	6	6	4	6	6	6	1	2	1	6	7	5	8	6	6	1	2	1
Q9P2E9	Ribosome-binding protein 1	RRBP1	1	19	19	4	9	8	7	7	7	2	1	1	7	9	8	7	7	7	2	1	1	7	9	8	7	7	7	2	1	1
P00491	Purine nucleoside phosphorylase	PNP	1	9	9	4	4	5	7	7	7	1	2	1	5	4	5	7	7	7	1	2	1	5	4	6	7	7	7	1	2	1
P27482	Calmodulin-like protein 3	CALML3	1	11	11	3	3	3	7	8	7	1	2	1	3	3	3	7	8	7	1	2	1	3	4	4	8	9	8	1	2	1
Q14134	Tripartite motif-containing protein 29	TRIM29	1	13	13	4	5	5	9	9	9	1	1	2	5	5	5	9	9	9	1	1	2	5	5	5	9	9	9	1	1	2
Q6UWP8	Suprabasin	SBSN	1	13	13	4	0	0	9	12	11	1	1	2	0	0	0	9	12	11	1	1	2	0	0	0	14	17	15	1	1	2
P14923	Junction plakoglobin	JUP	1	19	17	0	0	0	15	15	16	3	1	0	0	0	0	13	13	14	3	1	0	0	0	0	19	19	18	3	1	0
P68871	Hemoglobin subunit beta	HBB	7	10	10	8	8	8	0	0	0	0	2	1	8	8	8	0	0	0	0	2	1	9	8	9	0	0	0	0	2	1
P39687	Acidic leucine-rich nuclear phosphoprotein 32 family member A	ANP32A	3	8	7	7	6	6	0	0	0	1	1	1	6	5	5	0	0	0	1	1	1	8	6	6	0	0	0	1	1	1
P50995	Annexin A11	ANXA11	1	20	20	5	6	6	0	0	0	1	2	0	5	6	6	0	0	0	1	2	0	6	7	7	0	0	0	1	2	0
P04217	Alpha-1B-glycoprotein	A1BG	1	17	17	5	5	6	0	0	0	1	1	1	5	5	6	0	0	0	1	1	1	6	6	7	0	0	0	1	1	1
Q9H0W9	Ester hydrolase C11orf54	C11orf54	1	7	7	5	5	4	0	0	0	1	1	1	5	5	4	0	0	0	1	1	1	5	6	4	0	0	0	1	1	1
Q96FV2	Secernin-2	SCRN2	1	6	6	4	4	4	0	0	0	1	1	1	6	4	4	0	0	0	1	1	1	6	4	4	0	0	0	1	1	1
Q9H2H8	Peptidyl-prolyl cis-trans isomerase-like 3	PPIL3	1	6	6	4	3	2	0	0	0	1	1	1	5	3	2	0	0	0	1	1	1	5	3	3	0	0	0	1	1	1
P02511	Alpha-crystallin B chain	CRYAB	1	11	11	3	3	4	0	0	0	1	1	1	3	3	4	0	0	0	1	1	1	3	3	4	0	0	0	1	1	1
P55210	Caspase-7	CASP7	1	6	6	4	3	2	0	0	0	1	2	0	4	3	2	0	0	0	1	2	0	4	3	2	0	0	0	1	2	0
P40306	Proteasome subunit beta type-10	PSMB10	1	5	5	4	2	2	0	0	0	1	1	1	3	2	2	0	0	0	1	1	1	3	3	2	0	0	0	1	1	1
Q15369	Transcription elongation factor B polypeptide 1	TCEB1	1	3	3	4	3	3	0	0	0	1	1	1	2	3	3	0	0	0	1	1	1	2	3	3	0	0	0	1	1	1
O43278	Kunitz-type protease inhibitor 1	SPINT1	1	4	4	4	2	2	0	0	0	1	1	1	3	2	2	0	0	0	1	1	1	3	2	2	0	0	0	1	1	1
P09210	Glutathione S-transferase A2	GSTA2/A1	4	7	7	2	2	2	0	0	0	1	1	1	2	2	2	0	0	0	1	1	1	2	2	2	0	0	0	1	1	1
P62877	E3 ubiquitin-protein ligase RBX1	RBX1	1	2	2	4	2	2	0	0	0	1	1	1	2	2	2	0	0	0	1	1	1	2	2	2	0	0	0	1	1	1
Q99584	Protein S100-A13	S100A13	1	5	5	4	2	1	0	0	0	1	1	1	3	2	1	0	0	0	1	1	1	3	2	1	0	0	0	1	1	1
P05976	Myosin light chain 1/3, skeletal muscle isoform	MYL1;MYL3	2	1	1	1	1	1	0	0	0	1	1	1	1	1	1	0	0	0	1	1	1	2	2	1	0	0	0	1	1	1
P09104	Gamma-enolase	ENO2	1	5	2	4	4	4	2	3	3	3	3	2	1	1	1	0	0	0	1	1	0	3	1	1	0	0	0	1	2	0
Q9BVG4	Protein PBDC1	PBDC1	1	3	3	4	2	1	0	0	0	1	1	1	2	2	1	0	0	0	1	1	1	2	2	1	0	0	0	1	1	1
P24158	Myeloblastin	PRTN3	1	4	4	4	1	1	0	0	0	1	1	1	3	1	1	0	0	0	1	1	1	3	1	1	0	0	0	1	1	1
P30520	Adenylosuccinate synthetase isozyme 2	ADSS	2	10	10	2	1	1	0	0	0	0	1	2	2	1	1	0	0	0	0	1	2	2	1	1	0	0	0	0	1	2
O15511	Actin-related protein 2/3 complex subunit 5	ARPC5	1	3	3	1	2	1	0	0	0	1	0	2	1	2	1	0	0	0	1	0	2	1	2	1	0	0	0	1	0	2
P02656	Apolipoprotein C-III	APOC3	1	1	1	1	1	1	0	0	0	1	1	1	1	1	1	0	0	0	1	1	1	1	1	1	0	0	0	1	1	1
P60022	Beta-defensin 1	DEFB1	1	1	1	1	1	1	0	0	0	1	1	1	1	1	1	0	0	0	1	1	1	1	1	1	0	0	0	1	1	1

Q9H098	Protein FAM107B	FAM107B	1	2	1	1	1	1	0	0	0	1	1	1	1	1	1	0	0	0	1	1	1	1	1	1	0	0	0	1	1	1
Q96RP9	Elongation factor G, mitochondrial	GFM1	1	1	1	1	1	1	0	0	0	1	1	1	1	1	1	0	0	0	1	1	1	1	1	1	0	0	0	1	1	1
P17936	Insulin-like growth factor-binding protein 3	IGFBP3	1	1	1	1	1	1	0	0	0	1	1	1	1	1	1	0	0	0	1	1	1	1	1	1	0	0	0	1	1	1
P01704	Ig lambda chain V-II region TOG	IGKV2-14	1	1	1	4	1	1	0	0	0	1	1	1	1	1	1	0	0	0	1	1	1	1	1	1	0	0	0	1	1	1
P48444	Coatomer subunit delta	ARCN1	1	8	8	0	0	2	0	0	0	1	1	1	0	0	2	0	0	0	1	1	1	0	0	2	0	0	0	1	1	1
P49908	Selenoprotein P	SEPP1	1	2	2	4	1	0	0	0	0	1	1	1	1	1	0	0	0	0	1	1	1	1	1	0	0	0	0	1	1	1
P53621	Coatomer subunit alpha	COPA	1	18	18	0	1	0	0	0	0	1	1	1	0	1	0	0	0	0	1	1	1	0	1	0	0	0	0	1	1	1
Q96GG9	DCN1-like protein 1	DCUN1D1	1	2	2	0	0	1	0	0	0	1	1	1	0	0	1	0	0	0	1	1	1	0	0	1	0	0	0	1	1	1
P51148	Ras-related protein Rab-5C	RAB5C	1	6	3	4	0	0	0	0	0	1	1	1	1	0	0	0	0	0	1	1	1	1	0	0	0	0	0	1	1	1
Q04609	Glutamate carboxypeptidase 2	FOLH1	2	3	3	0	0	0	0	0	0	0	1	2	0	0	0	0	0	0	0	1	2	0	0	0	0	0	0	0	1	2
Q16222	UDP-N-acetylhexosamine pyrophosphorylase	UAP1	2	8	8	0	0	0	0	0	0	1	1	1	0	0	0	0	0	0	1	1	1	0	0	0	0	0	0	1	1	1
A0A0C4DH73	Ig kappa chain V-I region Wes	IGKV1-12	2	2	1	1	1	1	1	1	1	2	2	2	0	0	0	0	0	0	1	1	1	0	0	0	0	0	0	1	1	1
Q9H0U4	Ras-related protein Rab-1B	RAB1B/1C	2	7	3	1	1	1	3	2	2	3	3	3	0	0	0	0	0	0	1	1	1	0	0	0	0	0	0	1	1	1
Q9NT62	Ubiquitin-like-conjugating enzyme ATG3	ATG3	1	2	2	0	0	0	0	0	0	1	1	1	0	0	0	0	0	0	1	1	1	0	0	0	0	0	0	1	1	1
O95861	3(2),5-bisphosphate nucleotidase 1	BPNT1	1	13	13	0	0	0	0	0	0	1	1	1	0	0	0	0	0	0	1	1	1	0	0	0	0	0	0	1	1	1
P15502	Elastin	ELN	1	1	1	0	0	0	0	0	0	1	1	1	0	0	0	0	0	0	1	1	1	0	0	0	0	0	0	1	1	1
P46976	Glycogenin-1	GYG1	1	3	3	0	0	0	0	0	0	1	1	1	0	0	0	0	0	0	1	1	1	0	0	0	0	0	0	1	1	1
Q96RW7	Hemicentin-1	HMCN1	1	2	2	0	0	0	0	0	0	1	0	2	0	0	0	0	0	0	1	0	2	0	0	0	0	0	0	1	0	2
P51608	Methyl-CpG-binding protein 2	MECP2	1	1	1	0	0	0	0	0	0	1	1	1	0	0	0	0	0	0	1	1	1	0	0	0	0	0	0	1	1	1
Q9HAB8	Phosphopantothenate--cysteine ligase	PPCS	1	5	5	4	0	0	0	0	0	1	1	1	0	0	0	0	0	0	1	1	1	0	0	0	0	0	0	1	1	1
P10586	Receptor-type tyrosine-protein phosphatase F	PTPRF	1	3	3	4	0	0	0	0	0	1	1	1	0	0	0	0	0	0	1	1	1	0	0	0	0	0	0	1	1	1
P47897	Glutamine--tRNA ligase	QARS	1	12	12	4	0	0	0	0	0	1	1	1	0	0	0	0	0	0	1	1	1	0	0	0	0	0	0	1	1	1
Q9H788	SH2 domain-containing protein 4A	SH2D4A	1	4	4	4	0	0	0	0	0	1	1	1	0	0	0	0	0	0	1	1	1	0	0	0	0	0	0	1	1	1
P50591	Tumor necrosis factor ligand superfamily member 10	TNFSF10	1	2	2	4	0	0	0	0	0	1	1	1	0	0	0	0	0	0	1	1	1	0	0	0	0	0	0	1	1	1
Q8NBS9	Thioredoxin domain-containing protein 5	TXNDC5	1	4	4	4	0	0	0	0	0	1	1	1	0	0	0	0	0	0	1	1	1	0	0	0	0	0	0	1	1	1
P54578	Ubiquitin carboxyl-terminal hydrolase 14	USP14	1	10	10	4	0	0	0	0	0	0	1	2	0	0	0	0	0	0	0	1	2	0	0	0	0	0	0	0	1	2
Q8IZ83	Aldehyde dehydrogenase family 16 member A1	ALDH16A1	1	14	14	3	5	2	0	1	0	1	1	1	3	5	2	0	1	0	1	1	1	3	5	2	0	1	0	1	1	1
Q16204	Coiled-coil domain-containing protein 6	CCDC6	1	5	5	3	3	3	0	1	0	1	0	2	3	3	3	0	1	0	1	0	2	3	3	3	0	1	0	1	0	2
P08294	Extracellular superoxide dismutase [Cu-Zn]	SOD3	1	6	6	4	3	2	1	0	0	1	1	1	3	3	2	1	0	0	1	1	1	3	3	2	1	0	0	1	1	1
O15400	Syntaxin-7	STX7	1	4	4	4	2	2	1	0	0	1	1	1	1	2	2	1	0	0	1	1	1	1	2	2	1	0	0	1	1	1
Q14019	Coactosin-like protein	COTL1	1	2	2	1	2	1	0	1	0	1	1	1	1	2	1	0	1	0	1	1	1	1	2	1	0	1	0	1	1	1
P02760	Protein AMBP	AMBP	1	5	5	1	1	1	0	1	0	1	1	1	1	1	1	0	1	0	1	1	1	1	1	1	0	1	0	1	1	1
P55064	Aquaporin-5	AQP5	1	4	4	1	1	1	0	0	1	2	0	1	1	1	1	0	0	1	2	0	1	1	1	1	0	0	1	2	0	1
Q96QK1	Vacuolar protein sorting-associated protein 35	VPS35	1	8	8	4	1	1	0	1	0	1	1	1	1	1	1	0	1	0	1	1	1	1	1	1	0	1	0	1	1	1
A0A075B7D0	Ig-like domain-containing protein	IGHV1OR15-1	1	1	1	1	0	0	0	1	0	1	1	1	1	0	0	0	1	0	1	1	1	1	0	0	0	1	0	1	1	1
Q15113	Procollagen C-endopeptidase enhancer 1	PCOLCE	1	2	2	4	0	1	1	0	0	1	1	1	0	0	1	1	0	0	1	1	1	0	0	1	1	0	0	1	1	1
O15305	Phosphomannomutase 2	PMM2	2	6	6	0	0	0	1	0	0	1	1	1	0	0	0	1	0	0	1	1	1	0	0	0	1	0	0	1	1	1
O94985	Calsyntenin-1	CLSTN1	1	1	1	0	0	0	1	0	0	1	1	1	0	0	0	1	0	0	1	1	1	0	0	0	1	0	0	1	1	1
Q10471	Polypeptide N-acetylgalactosaminyltransferase 2	GALNT2	1	5	5	0	0	0	0	0	1	1	1	1	0	0	0	0	0	1	1	1	1	0	0	0	0	0	1	1	1	1
P28482	Mitogen-activated protein kinase 1	MAPK1	1	9	7	0	0	0	1	0	0	1	1	1	0	0	0	1	0	0	1	1	1	0	0	0	1	0	0	1	1	1
Q13492	Phosphatidylinositol-binding clathrin assembly protein	PICALM	1	2	2	4	0	0	0	0	1	1	1	1	0	0	0	0	0	1	1	1	1	0	0	0	0	0	1	1	1	1
A0AVT1	Ubiquitin-like modifier-activating enzyme 6	UBA6	1	5	5	4	0	0	0	0	1	1	2	0	0	0	0	0	0	1	1	2	0	0	0	0	0	0	1	1	2	0
P21291	Cysteine and glycine-rich protein 1	CSRP1	1	8	8	5	6	5	1	0	1	2	0	1	5	6	5	1	0	1	2	0	1	6	7	5	1	0	1	2	0	1
P05452	Tetranectin	CLEC3B	2	9	9	5	6	5	1	0	1	1	1	1	5	6	5	1	0	1	1	1	1	5	6	5	1	0	1	1	1	1
P43487	Ran-specific GTPase-activating protein	RANBP1	1	5	5	4	5	3	1	0	1	1	1	1	3	5	3	1	0	1	1	1	1	5	7	4	1	0	1	1	1	1
P31146	Coronin-1A	CORO1A	1	12	12	3	3	2	1	1	0	1	1	1	3	3	2	1	1	0	1	1	1	3	3	2	1	1	0	1	1	1
Q9H444	Charged multivesicular body protein 4b	CHMP4B	2	3	3	3	2	1	1	1	0	1	1	1	3	2	1	1	1	0	1	1	1	3	2	1	1	1	0	1	1	1
O15145	Actin-related protein 2/3 complex subunit 3	ARPC3	1	3	3	2	2	1	0	1	1	1	0	2	2	2	1	0	1	1	1	0	2	3	2	1	0	1	1	1	0	2
P30048	Thioredoxin-dependent peroxide reductase, mitochondrial	PRDX3	1	6	6	4	2	2	1	0	1	2	1	0	2	2	2	1	0	1	2	1	0	2	2	2	1	0	1	2	1	0
Q9H299	SH3 domain-binding glutamic acid-rich-like protein 3	SH3BGRL3	1	4	4	4	1	3	1	1	0	1	1	1	2	1	3	1	1	0	1	1	1	2	1	3	1	1	0	1	1	1

Q9Y5P6	Mannose-1-phosphate guanyltransferase beta	GMPPB	1	5	5	1	1	1	1	1	0	1	1	1	1	1	1	1	1	0	1	1	1	1	1	1	1	1	0	1	1	1
P15814	Immunoglobulin lambda-like polypeptide 1	IGLL1	1	1	1	1	1	1	1	1	0	1	1	1	1	1	1	1	1	0	1	1	1	1	1	1	1	1	0	1	1	1
Q9UNH7	Sorting nexin-6	SNX6	2	8	8	1	0	0	0	1	1	0	1	2	1	0	0	0	1	1	0	1	2	1	0	0	0	1	1	0	1	2
O75460	Serine/threonine-protein kinase/endoribonuclease IRE1	ERN1	1	1	1	0	0	1	1	0	1	1	1	1	0	0	1	1	0	1	1	1	1	0	0	1	1	0	1	1	1	1
A0A0C4DH25	Immunoglobulin kappa variable 3D-20	IGKV3D-20	1	3	1	2	2	3	1	2	3	3	3	3	0	0	1	0	1	1	1	1	1	0	0	1	0	1	1	1	1	1
P16519	Neuroendocrine convertase 2	PCSK2	1	1	1	4	1	0	1	1	0	1	1	1	0	1	0	1	1	0	1	1	1	0	1	0	1	1	0	1	1	1
O95969	Secretoglobin family 1D member 2	SCGB1D2	1	1	1	4	0	1	0	1	1	1	1	1	0	0	1	0	1	1	1	1	1	0	0	1	0	1	1	1	1	1
Q14142	Tripartite motif-containing protein 14	TRIM14	1	1	1	4	0	0	1	1	0	1	1	1	1	0	0	1	1	0	1	1	1	1	0	0	1	1	0	1	1	1
P36543	V-type proton ATPase subunit E 1	ATP6V1E1	1	4	4	0	0	0	1	1	0	1	1	1	0	0	0	1	1	0	1	1	1	0	0	0	1	1	0	1	1	1
O00204	Sulfotransferase family cytosolic 2B member 1	SULT2B1	1	4	4	4	0	0	1	0	1	1	1	1	0	0	0	1	0	1	1	1	1	0	0	0	1	0	1	1	1	1
P13798	Acylamino-acid-releasing enzyme	APEH	1	16	16	12	10	13	1	1	1	1	1	1	12	10	13	1	1	1	1	1	1	15	11	14	1	1	1	1	1	1
Q16531	DNA damage-binding protein 1	DDB1	1	14	14	9	6	7	1	1	1	1	1	1	9	6	7	1	1	1	1	1	1	9	6	7	1	1	1	1	1	1
P55263	Adenosine kinase	ADK	1	10	10	3	4	5	1	1	1	1	1	1	3	4	5	1	1	1	1	1	1	3	4	5	1	1	1	1	1	1
Q9UNZ2	NSFL1 cofactor p47	NSFL1C	1	5	5	4	4	4	1	2	0	1	2	0	3	4	4	1	2	0	1	2	0	3	4	4	1	2	0	1	2	0
Q15370	Transcription elongation factor B polypeptide 2	TCEB2	1	3	3	4	2	3	1	1	1	1	1	1	3	2	3	1	1	1	1	1	1	3	2	3	1	1	1	1	1	1
P61956	Small ubiquitin-related modifier 2	SUMO2	3	3	3	3	2	1	1	1	1	1	1	1	3	2	1	1	1	1	1	1	1	3	2	2	1	1	1	1	1	1
Q01105	Protein SET	SET;SETSIP	2	6	6	2	2	2	1	1	1	2	1	0	2	2	2	1	1	1	2	1	0	2	2	2	1	1	1	2	1	0
O60888	Protein CutA	CUTA	1	2	2	1	1	2	1	1	1	1	1	1	1	1	2	1	1	1	1	1	1	1	1	2	1	1	1	1	1	1
O60784	Target of Myb protein 1	TOM1	1	5	5	4	1	2	1	1	1	1	1	1	1	1	2	1	1	1	1	1	1	1	1	2	1	1	1	1	1	1
A2NJV5	Immunoglobulin kappa variable 2-29	A18;IGKV2D-26;IGKV2	3	3	1	2	2	2	2	2	2	2	2	2	1	1	1	1	1	1	1	1	1	1	1	1	1	1	1	1	1	1
P17066	Heat shock 70 kDa protein 6	HSPA6/A7	2	9	1	8	8	9	6	7	8	8	8	7	1	1	1	1	1	1	1	1	1	1	1	1	1	1	1	1	1	1
O43505	Beta-1,4-glucuronyltransferase 1	B4GAT1	1	2	2	1	1	1	1	1	1	1	1	1	1	1	1	1	1	1	1	1	1	1	1	1	1	1	1	1	1	1
Q8WXG9	G-protein coupled receptor 98	GPR98	1	2	2	1	1	1	1	1	1	1	1	1	1	1	1	1	1	1	1	1	1	1	1	1	1	1	1	1	1	1
Q6UXB2	VEGF coregulated chemokine 1	CXCL17	1	6	6	2	0	0	1	0	2	2	0	1	2	0	0	1	0	2	2	0	1	2	0	0	1	0	2	2	0	1
O00757	Fructose-1,6-bisphosphatase isozyme 2	FBP2	1	3	1	2	1	2	1	1	1	2	2	2	1	0	1	1	1	1	1	1	1	1	0	1	1	1	1	1	1	1
Q13162	Peroxiredoxin-4	PRDX4	1	3	1	4	1	2	2	2	2	2	2	2	1	0	1	1	1	1	1	1	1	1	0	1	1	1	1	1	1	1
P35321	Cornifin-A	SPRR1A	1	3	1	4	2	1	2	2	3	2	2	2	1	1	0	1	1	1	1	1	1	1	1	0	1	1	1	1	1	1
Q9NP79	Vacuolar protein sorting-associated protein VTA1 homolog	VTA1	1	4	3	4	1	1	1	1	1	1	1	1	0	1	1	1	1	1	1	1	1	0	1	1	1	1	1	1	1	1
P04430	Ig kappa chain V-I region BAN	IGKV1-16	1	1	1	4	1	0	1	1	1	1	1	1	1	1	0	1	1	1	1	1	1	1	1	0	1	1	1	1	1	1
Q15056	Eukaryotic translation initiation factor 4H	EIF4H	1	3	3	0	1	0	1	1	1	1	1	1	0	1	0	1	1	1	1	1	1	0	1	0	1	1	1	1	1	1
P0DP01	Immunoglobulin heavy variable 1-8	IGHV1-8	1	2	1	1	2	0	2	2	2	2	2	2	0	1	0	1	1	1	1	1	1	0	1	0	1	1	1	1	1	1
P01599	Ig kappa chain V-I region Gal	IGKV1-17	1	3	1	4	2	1	2	2	3	2	2	3	0	1	0	1	1	1	1	1	1	0	1	0	1	1	1	1	1	1
P05198	Eukaryotic translation initiation factor 2 subunit 1	EIF2S1	1	2	2	0	0	0	1	1	1	1	1	1	0	0	0	1	1	1	1	1	1	0	0	0	1	1	1	1	1	1
Q14974	Importin subunit beta-1	KPNB1	1	8	8	0	0	0	1	1	1	1	1	1	0	0	0	1	1	1	1	1	1	0	0	0	1	1	1	1	1	1
P62195	26S protease regulatory subunit 8	PSMC5	1	7	7	4	0	0	1	1	1	1	1	1	0	0	0	1	1	1	1	1	1	0	0	0	1	1	1	1	1	1
Q16181	Septin-7	SEPTIN7	1	6	6	4	0	0	1	1	1	1	1	1	0	0	0	1	1	1	1	1	1	0	0	0	1	1	1	1	1	1
Q8IWU5	Extracellular sulfatase Sulf-2	SULF2	1	3	3	4	0	0	2	0	1	1	1	1	0	0	0	2	0	1	1	1	1	0	0	0	2	0	1	1	1	1
Q9Y3F4	Serine-threonine kinase receptor-associated protein	STRAP	1	8	8	4	4	6	2	1	1	1	1	1	6	4	6	2	1	1	1	1	1	6	5	6	2	1	1	1	1	1
Q15843	NEDD8	NEDD8	1	4	4	4	2	2	1	1	2	1	1	1	2	2	2	1	1	2	1	1	1	3	3	2	1	1	2	1	1	1
P28325	Cystatin-D	CST5	1	2	2	0	0	0	1	2	1	1	1	1	0	0	0	1	2	1	1	1	1	0	0	0	1	2	1	1	1	1
Q9GZM7	Tubulointerstitial nephritis antigen-like	TINAGL1	1	4	4	4	0	0	1	0	3	0	1	2	0	0	0	1	0	3	0	1	2	0	0	0	1	0	3	0	1	2
P01594	Ig kappa chain V-I region AU	IGKV1-33	2	1	1	1	1	1	1	1	1	1	1	1	1	1	1	1	1	1	1	1	1	1	1	1	2	1	2	1	1	1
P35606	Coatomer subunit beta	COPB2	1	13	13	0	0	0	2	2	1	1	1	1	0	0	0	2	2	1	1	1	1	0	0	0	2	2	1	1	1	1
P08582	Melanotransferrin	MFI2	1	5	5	0	0	0	2	1	2	1	1	1	0	0	0	2	1	2	1	1	1	0	0	0	2	1	2	1	1	1
Q13200	26S proteasome non-ATPase regulatory subunit 2	PSMD2	1	5	5	4	0	0	2	1	2	1	1	1	0	0	0	2	1	2	1	1	1	0	0	0	2	1	2	1	1	1
P06753	Tropomyosin alpha-3 chain	TPM3	1	15	10	4	6	9	5	5	5	4	4	2	6	4	5	2	2	2	2	1	0	6	4	5	2	2	2	2	1	0
P16070	CD44 antigen	CD44	1	3	3	3	3	3	2	2	2	2	1	0	3	3	3	2	2	2	2	1	0	3	3	4	2	2	2	2	1	0
Q96HE7	ERO1-like protein alpha	ERO1L	1	9	9	2	2	2	2	2	2	1	1	1	2	2	2	2	2	2	1	1	1	2	2	2	2	2	2	1	1	1
P10253	Lysosomal alpha-glucosidase	GAA	1	4	4	0	2	2	3	2	1	1	1	1	0	2	2	3	2	1	1	1	1	0	2	2	3	2	1	1	1	1
Q15366	Poly(rC)-binding protein 2	PCBP2	2	7	5	1	1	0	2	3	2	1	2	2	0	0	0	2	2	2	1	1	1	0	0	0	2	2	2	1	1	1

Q99832	T-complex protein 1 subunit eta	CCT7	1	15	15	0	0	0	2	4	0	1	1	1	0	0	0	2	4	0	1	1	1	0	0	0	2	4	0	1	1	1
Q14393	Growth arrest-specific protein 6	GAS6	1	5	5	0	0	0	2	2	2	1	1	1	0	0	0	2	2	2	1	1	1	0	0	0	2	2	2	1	1	1
Q04760	Lactoylglutathione lyase	GLO1	1	5	5	4	4	2	3	2	2	1	1	1	4	4	2	3	2	2	1	1	1	5	4	2	3	2	2	1	1	1
O95274	Ly6/PLAUR domain-containing protein 3	LYPD3	1	3	3	1	0	0	3	2	2	1	1	1	1	0	0	3	2	2	1	1	1	1	0	0	3	2	2	1	1	1
Q9HCY8	Protein S100-A14	S100A14	1	3	3	4	2	1	3	3	2	1	1	1	1	2	1	3	3	2	1	1	1	1	2	1	3	3	2	1	1	1
Q16543	Hsp90 co-chaperone Cdc37	CDC37	1	8	8	0	0	0	4	1	3	1	1	1	0	0	0	4	1	3	1	1	1	0	0	0	4	1	3	1	1	1
Q99574	Neuroserpin	SERPINI1	1	5	5	4	0	0	3	2	3	1	1	1	0	0	0	3	2	3	1	1	1	0	0	0	3	2	3	1	1	1
P62241	40S ribosomal protein S8	RPS8	1	8	8	4	4	4	2	4	4	1	1	1	5	4	4	2	4	4	1	1	1	7	5	4	2	4	4	1	1	1
P01859	Ig gamma-2 chain C region	IGHG2	1	8	5	6	6	5	4	5	6	3	2	2	3	3	3	2	2	3	1	1	1	4	4	4	3	3	4	1	1	1
P52597	Heterogeneous nuclear ribonucleoprotein F	HNRNPF	1	5	3	1	2	1	4	3	4	1	1	1	0	1	0	2	2	2	0	0	0	1	2	1	4	3	4	1	1	1
P09958	Furin	FURIN	1	9	9	0	1	0	4	4	4	1	1	1	0	1	0	4	4	4	1	1	1	0	1	0	4	4	4	1	1	1
P07814	Bifunctional glutamate/proline--tRNA ligase	EPRS	1	20	20	2	2	1	4	4	5	1	1	1	2	2	1	4	4	5	1	1	1	2	2	1	4	4	5	1	1	1
P26641	Elongation factor 1-gamma	EEF1G	1	10	9	1	1	1	5	4	5	1	1	1	1	1	1	5	4	5	1	1	1	1	1	1	5	4	5	1	1	1
P25685	DnaJ homolog subfamily B member 1	DNAJB1	2	6	6	0	0	0	5	5	4	1	1	1	0	0	0	5	5	4	1	1	1	0	0	0	5	5	4	1	1	1
P51991	Heterogeneous nuclear ribonucleoprotein A3	HNRNPA3	1	8	8	1	2	1	5	5	5	1	1	1	1	2	1	5	5	5	1	1	1	1	2	1	5	5	5	1	1	1
P06576	ATP synthase subunit beta, mitochondrial	ATP5B	1	20	20	5	4	3	5	5	6	1	1	1	5	4	3	5	5	6	1	1	1	6	4	3	6	5	7	1	1	1
Q13813	Spectrin alpha chain, non-erythrocytic 1	SPTAN1	1	38	38	4	0	0	5	10	4	0	2	1	2	0	0	5	10	4	0	2	1	2	0	0	5	10	4	0	2	1
P11940	Polyadenylate-binding protein 1	PABPC1	4	12	12	3	2	4	7	8	6	1	1	1	3	2	4	7	8	6	1	1	1	3	2	4	8	8	6	1	1	1
Q99878	Histone H2A type 1-J	2AH;H2AFJ;HIST1H2AG;	7	11	0	6	5	5	6	6	6	1	1	1	0	0	0	0	0	0	0	0	0	6	6	6	9	9	8	1	1	1
Q96HC4	PDZ and LIM domain protein 5	PDLIM5	1	18	18	4	10	9	0	0	0	1	1	0	11	10	9	0	0	0	1	1	0	12	10	10	0	0	0	1	1	0
Q14126	Desmoglein-2	DSG2	1	13	13	7	9	8	0	0	0	1	1	0	7	9	8	0	0	0	1	1	0	7	9	8	0	0	0	1	1	0
P43652	Afamin	AFM	1	21	21	6	6	6	0	0	0	0	1	1	6	6	6	0	0	0	0	1	1	6	6	6	0	0	0	0	1	1
A0MZ66	Shootin-1	KIAA1598	1	10	10	4	5	7	0	0	0	0	1	1	4	5	7	0	0	0	0	1	1	4	5	7	0	0	0	0	1	1
O94760	N(G),N(G)-dimethylarginine dimethylaminohydrolase 1	DDAH1	1	12	12	4	4	5	0	0	0	1	1	0	4	4	5	0	0	0	1	1	0	5	5	5	0	0	0	1	1	0
P35237	Serpin B6	SERPINB6	1	13	13	4	4	6	0	0	0	0	0	2	5	4	6	0	0	0	0	0	2	5	4	6	0	0	0	0	0	2
P36969	Phospholipid hydroperoxide glutathione peroxidase, mitochondrial	GPX4	1	4	4	3	4	3	0	0	0	1	1	0	3	4	3	0	0	0	1	1	0	3	5	4	0	0	0	1	1	0
Q92882	Osteoclast-stimulating factor 1	OSTF1	1	5	5	4	2	3	0	0	0	1	0	1	3	2	3	0	0	0	1	0	1	3	2	3	0	0	0	1	0	1
P62888	60S ribosomal protein L30	RPL30	1	5	5	4	3	3	0	0	0	0	1	1	2	3	3	0	0	0	0	1	1	2	3	3	0	0	0	0	1	1
P19827	Inter-alpha-trypsin inhibitor heavy chain H1	ITIH1	2	18	18	2	2	2	0	0	0	1	0	1	2	2	2	0	0	0	1	0	1	2	2	2	0	0	0	1	0	1
O60234	Glia maturation factor gamma	GMFG	1	2	2	2	2	2	0	0	0	0	1	1	2	2	2	0	0	0	0	1	1	2	2	2	0	0	0	0	1	1
Q14116	Interleukin-18	IL18	1	5	5	3	2	1	0	0	0	0	1	1	3	2	1	0	0	0	0	1	1	3	2	1	0	0	0	0	1	1
Q8TCD5	5(3)-deoxyribonucleotidase, cytosolic type	NT5C	1	5	5	4	2	2	0	0	0	0	1	1	2	2	2	0	0	0	0	1	1	2	2	2	0	0	0	0	1	1
P09455	Retinol-binding protein 1	RBP1	1	5	5	4	2	1	0	0	0	1	1	0	2	2	1	0	0	0	1	1	0	2	2	1	0	0	0	1	1	0
P07902	Galactose-1-phosphate uridylyltransferase	GALT	1	2	2	1	1	2	0	0	0	0	1	1	1	1	2	0	0	0	0	1	1	1	1	2	0	0	0	0	1	1
Q9NQW7	Xaa-Pro aminopeptidase 1	XPNPEP1	1	11	11	4	1	1	0	0	0	1	0	1	2	1	1	0	0	0	1	0	1	2	1	1	0	0	0	1	0	1
P08246	Neutrophil elastase	ELANE	1	6	6	1	1	1	0	0	0	0	1	1	1	1	1	0	0	0	0	1	1	1	1	1	0	0	0	0	1	1
Q02790	Peptidyl-prolyl cis-trans isomerase FKBP4	FKBP4	1	6	6	2	1	0	0	0	0	0	1	1	2	1	0	0	0	0	0	1	1	2	1	0	0	0	0	0	1	1
P01743	Ig heavy chain V-I region HG3	IGHV1-46	1	2	1	2	2	1	1	1	1	2	1	2	1	1	1	0	0	0	1	0	1	1	1	1	0	0	0	1	0	1
Q9H5N1	Rab GTPase-binding effector protein 2	RABEP2	1	1	1	4	1	1	0	0	0	1	0	1	1	1	1	0	0	0	1	0	1	1	1	1	0	0	0	1	0	1
Q9NVA2	Septin-11	SEPTIN11	1	7	6	4	1	1	0	0	0	1	0	1	1	1	1	0	0	0	1	0	1	1	1	1	0	0	0	1	0	1
O00468	Agrin	AGRN	1	1	1	1	1	0	0	0	0	1	0	1	1	1	0	0	0	0	1	0	1	1	1	0	0	0	0	1	0	1
Q9Y365	PCTP-like protein	STARD10	1	5	5	4	1	0	0	0	0	0	0	2	1	1	0	0	0	0	0	0	2	1	1	0	0	0	0	0	0	2
P50552	Vasodilator-stimulated phosphoprotein	VASP	1	2	2	4	1	1	0	0	0	0	1	1	0	1	1	0	0	0	0	1	1	0	1	1	0	0	0	0	1	1
Q8IWE2	Protein NOXP20	FAM114A1	1	5	5	0	1	0	0	0	0	0	1	1	0	1	0	0	0	0	0	1	1	0	1	0	0	0	0	0	1	1
P07332	Tyrosine-protein kinase Fes/Fps	FES	1	2	2	0	0	1	0	0	0	0	1	1	0	0	1	0	0	0	0	1	1	0	0	1	0	0	0	0	1	1
A0A075B6K4	Ig lambda chain V-IV region Hil	IGLV3-10	2	2	2	0	0	0	0	0	0	0	1	1	0	0	0	0	0	0	0	1	1	0	0	0	0	0	0	0	1	1
P17812	CTP synthase 1	CTPS1	2	12	12	0	0	0	0	0	0	0	1	1	0	0	0	0	0	0	0	1	1	0	0	0	0	0	0	0	1	1
Q96PD5	N-acetylmuramoyl-L-alanine amidase	PGLYRP2	2	11	11	0	0	0	0	0	0	1	0	1	0	0	0	0	0	0	1	0	1	0	0	0	0	0	0	1	0	1
P20591	Interferon-induced GTP-binding protein Mx1	MX1	2	9	9	0	0	0	0	0	0	0	1	1	0	0	0	0	0	0	0	1	1	0	0	0	0	0	0	0	1	1
Q9Y6Q5	AP-1 complex subunit mu-2	AP1M2	1	6	6	0	0	0	0	0	0	1	0	1	0	0	0	0	0	0	1	0	1	0	0	0	0	0	0	1	0	1

O43916	Carbohydrate sulfotransferase 1	CHST1	1	1	1	0	0	0	0	0	0	1	0	1	0	0	0	0	0	0	1	0	1	0	0	0	0	0	0	1	0	1
Q9UBT3	Dickkopf-related protein 4	DKK4	1	3	3	0	0	0	0	0	0	2	0	0	0	0	0	0	0	0	2	0	0	0	0	0	0	0	0	2	0	0
P51452	Dual specificity protein phosphatase 3	DUSP3	1	5	5	0	0	0	0	0	0	1	0	1	0	0	0	0	0	0	1	0	1	0	0	0	0	0	0	1	0	1
Q9NTX5	Ethylmalonyl-CoA decarboxylase	ECHDC1	1	3	3	0	0	0	0	0	0	0	1	1	0	0	0	0	0	0	0	1	1	0	0	0	0	0	0	0	1	1
Q9Y624	Junctional adhesion molecule A	F11R	1	3	3	0	0	0	0	0	0	0	1	1	0	0	0	0	0	0	0	1	1	0	0	0	0	0	0	0	1	1
Q9UHD0	Interleukin-19	IL19	1	2	2	0	0	0	0	0	0	0	1	0	0	0	0	0	0	0	0	1	0	0	0	0	0	0	0	0	2	0
P27361	Mitogen-activated protein kinase 3	MAPK3	1	8	6	0	0	0	0	0	0	1	1	0	0	0	0	0	0	0	1	1	0	0	0	0	0	0	0	1	1	0
Q9Y266	Nuclear migration protein nudC	NUDC	1	2	2	4	0	0	0	0	0	0	1	1	0	0	0	0	0	0	0	1	1	0	0	0	0	0	0	0	1	1
Q53EL6	Programmed cell death protein 4	PDCD4	1	9	9	4	0	0	0	0	0	1	0	1	0	0	0	0	0	0	1	0	1	0	0	0	0	0	0	1	0	1
P35080	Profilin-2	PFN2	1	2	2	4	0	0	0	0	0	1	0	1	0	0	0	0	0	0	1	0	1	0	0	0	0	0	0	1	0	1
Q96FV3	Tetraspanin-17	TSPAN17	1	1	1	4	0	0	0	0	0	1	0	1	0	0	0	0	0	0	1	0	1	0	0	0	0	0	0	1	0	1
Q7Z5L0	Vitelline membrane outer layer protein 1 homolog	VMO1	1	1	1	4	0	0	0	0	0	0	1	1	0	0	0	0	0	0	0	1	1	0	0	0	0	0	0	0	1	1
A0A0G2JRQ6	Ig-like domain-containing protein	ENSG00000281759	1	3	3	4	0	0	0	0	0	1	0	1	0	0	0	0	0	0	1	0	1	0	0	0	0	0	0	1	0	1
Q12904	Aminoacyl tRNA synthase complex-interacting multifunctional protein 1	AIMP1	1	7	7	5	5	6	0	0	1	1	1	0	5	5	6	0	0	1	1	1	0	5	5	6	0	0	1	1	1	0
P07954	Fumarate hydratase, mitochondrial	FH	1	9	9	4	4	5	0	0	1	1	1	0	4	4	5	0	0	1	1	1	0	5	5	6	0	0	1	1	1	0
P34949	Mannose-6-phosphate isomerase	MPI	1	7	7	4	3	4	0	1	0	0	2	0	4	3	4	0	1	0	0	2	0	4	3	4	0	1	0	0	2	0
Q14624	Inter-alpha-trypsin inhibitor heavy chain H4	ITIH4	1	19	19	3	3	4	1	0	0	0	1	1	3	3	4	1	0	0	0	1	1	3	3	4	1	0	0	0	1	1
P25325	3-mercaptopyruvate sulfurtransferase	MPST	1	7	7	3	3	3	1	0	0	0	1	1	3	3	3	1	0	0	0	1	1	3	3	3	1	0	0	0	1	1
P31941	DNA dC->dU-editing enzyme APOBEC-3A	APOBEC3A/3B	2	5	5	2	2	2	0	0	1	1	0	1	2	2	2	0	0	1	1	0	1	2	2	2	0	0	1	1	0	1
P09382	Galectin-1	LGALS1	1	3	3	2	2	2	0	1	0	1	0	1	2	2	2	0	1	0	1	0	1	2	2	2	0	1	0	1	0	1
P51857	3-oxo-5-beta-steroid 4-dehydrogenase	AKR1D1	1	2	2	1	2	1	1	0	0	0	1	1	1	2	1	1	0	0	0	1	1	1	2	1	1	0	0	0	1	1
P62910	60S ribosomal protein L32	RPL32	1	2	2	4	2	1	1	0	0	1	1	0	1	2	1	1	0	0	1	1	0	1	2	1	1	0	0	1	1	0
P0DJI8	Serum amyloid A-1 protein	SAA1;SAA2	2	2	2	1	1	1	1	0	0	0	1	1	1	1	1	1	0	0	0	1	1	1	1	1	1	0	0	0	1	1
Q7L5Y9	Macrophage erythroblast attacher	MAEA	1	1	1	0	0	1	0	1	0	1	0	1	0	0	1	0	1	0	1	0	1	0	0	1	0	1	0	1	0	1
O00264	Membrane-associated progesterone receptor component 1	PGRMC1	1	2	2	4	0	0	1	0	0	1	1	0	1	0	0	1	0	0	1	1	0	1	0	0	1	0	0	1	1	0
Q15437	Protein transport protein Sec23B	SEC23B	2	8	8	0	0	0	1	0	0	0	1	1	0	0	0	1	0	0	0	1	1	0	0	0	1	0	0	0	1	1
Q99439	Calponin-2	CNN2	1	3	3	0	0	0	1	0	0	1	0	1	0	0	0	1	0	0	1	0	1	0	0	0	1	0	0	1	0	1
Q3ZCM7	Tubulin beta-8 chain	TUBB8	1	7	2	4	1	1	1	2	2	3	2	4	0	0	0	0	0	1	1	0	1	0	0	0	0	0	1	1	0	1
P26583	High mobility group protein B2	HMGB2	1	7	4	6	5	5	1	1	0	1	1	2	3	3	2	1	1	0	1	0	1	3	3	2	1	1	0	1	0	1
P30040	Endoplasmic reticulum resident protein 29	ERP29	1	6	6	1	3	2	1	1	0	0	1	1	1	3	2	1	1	0	0	1	1	1	3	2	1	1	0	0	1	1
Q6UX06	Olfactomedin-4	OLFM4	1	3	3	4	1	1	1	1	0	0	1	1	1	1	1	1	1	0	0	1	1	2	1	2	1	1	0	0	1	1
P00736	Complement C1r subcomponent	C1R	1	12	12	2	1	1	1	1	0	2	0	0	2	1	1	1	1	0	2	0	0	2	1	1	1	1	0	2	0	0
P32320	Cytidine deaminase	CDA	1	2	2	1	1	1	1	1	0	0	1	1	1	1	1	1	1	0	0	1	1	1	1	1	1	1	0	0	1	1
P07305	Histone H1.0	H1F0	1	1	1	0	1	1	1	1	0	0	1	1	0	1	1	1	1	0	0	1	1	0	1	1	1	1	0	0	1	1
P84077	ADP-ribosylation factor 1	ARF1;ARF3	2	10	7	0	0	0	1	1	0	1	0	1	0	0	0	0	0	0	0	0	0	0	0	0	1	1	0	1	0	1
Q07960	Rho GTPase-activating protein 1	ARHGAP1	1	5	5	0	0	0	0	1	1	0	1	1	0	0	0	0	1	1	0	1	1	0	0	0	0	1	1	0	1	1
Q92520	Protein FAM3C	FAM3C	1	1	1	0	0	0	0	1	1	1	0	1	0	0	0	0	1	1	1	0	1	0	0	0	0	1	1	1	0	1
P26572	Alpha-1,3-mannosyl-glycoprotein 2-beta-N-acetylglucosaminyltransferase	MGAT1	1	2	2	0	0	0	0	1	1	0	1	1	0	0	0	0	1	1	0	1	1	0	0	0	0	1	1	0	1	1
Q9HC84	Mucin-5B	MUC5B	1	6	2	4	3	2	1	1	0	1	1	0	0	0	0	1	1	0	1	1	0	0	0	0	1	1	0	1	1	0
P61026	Ras-related protein Rab-10	RAB10	1	5	4	4	0	0	2	1	0	1	1	0	0	0	0	1	1	0	1	1	0	0	0	0	1	1	0	1	1	0
P46776	60S ribosomal protein L27a	RPL27A	1	3	3	4	3	2	1	0	2	1	0	1	3	3	2	1	0	2	1	0	1	3	3	2	1	0	2	1	0	1
P21926	CD9 antigen	CD9	1	2	2	2	2	2	1	1	1	1	0	1	2	2	2	1	1	1	1	0	1	2	2	2	1	1	1	1	0	1
P84098	60S ribosomal protein L19	RPL19	1	3	3	4	1	1	1	1	1	0	1	1	2	1	1	1	1	1	0	1	1	2	1	1	1	1	1	0	1	1
P07357	Complement component C8 alpha chain	C8A	1	4	4	1	1	1	1	1	1	0	1	1	1	1	1	1	1	1	0	1	1	1	1	1	1	1	1	0	1	1
Q96Q89	Kinesin-like protein KIF20B	KIF20B	1	1	1	1	1	1	1	1	1	1	0	1	1	1	1	1	1	1	1	0	1	1	1	1	1	1	1	1	0	1
A0A075B6H7	Ig-like domain-containing protein	3-7;IGKV3OR2-268;IGKV	3	2	1	1	2	2	2	2	2	2	1	2	0	1	1	1	1	1	1	0	1	0	1	1	1	1	1	1	0	1
P01764	Ig heavy chain V-III region 23	IGHV3-23	1	5	1	1	2	2	2	2	2	2	2	1	0	0	0	0	0	0	0	0	0	0	1	1	1	1	1	1	1	0
P63000	Ras-related C3 botulinum toxin substrate 1	RAC1	1	6	2	4	0	1	2	1	2	1	1	1	0	0	0	0	0	0	0	0	0	1	0	1	1	1	1	1	1	0
P20290	Transcription factor BTF3	BTF3	1	1	1	0	0	1	1	1	1	1	0	1	0	0	1	1	1	1	1	0	1	0	0	1	1	1	1	1	0	1
O75503	Ceroid-lipofuscinosis neuronal protein 5	CLN5	1	1	1	0	0	0	1	1	1	0	1	1	0	0	0	1	1	1	0	1	1	0	0	0	1	1	1	0	1	1

O43405	Cochlin	COCH	1	3	3	0	0	0	1	1	1	1	1	0	0	0	0	1	1	1	1	1	0	0	0	0	1	1	1	1	1	0
O00115	Deoxyribonuclease-2-alpha	DNASE2	1	1	1	0	0	0	1	1	1	1	1	0	0	0	0	1	1	1	1	1	0	0	0	0	1	1	1	1	1	0
Q13232	Nucleoside diphosphate kinase 3	NME3	1	4	4	4	0	0	1	2	0	1	0	1	0	0	0	1	2	0	1	0	1	0	0	0	1	2	0	1	0	1
P67775	Serine/threonine-protein phosphatase 2A catalytic subunit alpha isoform	PPP2CA	1	8	1	4	6	4	2	2	2	2	0	1	0	0	0	1	1	1	1	0	1	0	0	0	1	1	1	1	0	1
P61353	60S ribosomal protein L27	RPL27	1	3	3	4	1	2	1	2	1	0	1	1	2	1	2	1	2	1	0	1	1	2	1	2	1	2	1	0	1	1
Q16706	Alpha-mannosidase 2	MAN2A1	2	4	4	1	0	0	1	2	1	1	0	1	1	0	0	1	2	1	1	0	1	1	0	0	1	2	1	1	0	1
P15374	Ubiquitin carboxyl-terminal hydrolase isozyme L3	UCHL3	1	5	5	4	2	1	1	2	2	0	1	1	2	2	1	1	2	2	0	1	1	2	2	1	1	2	2	0	1	1
P04004	Vitronectin	VTN	2	9	9	1	1	2	2	2	1	1	1	0	1	1	2	2	2	1	1	1	0	1	1	2	2	2	1	1	1	0
Q7KZF4	Staphylococcal nuclease domain-containing protein 1	SND1	1	14	14	4	0	0	2	1	2	1	0	1	0	0	0	2	1	2	1	0	1	0	0	0	2	1	2	1	0	1
A0A0C4DH31	Immunoglobulin heavy variable 1-18	IGHV1-18	1	3	2	2	3	3	3	2	2	1	0	2	2	2	2	2	2	2	1	0	1	2	2	2	2	2	2	1	0	1
Q8TE68	Epidermal growth factor receptor kinase substrate 8-like protein 1	EPS8L1	1	9	9	1	1	0	4	1	1	0	1	1	1	1	0	4	1	1	0	1	1	1	1	0	4	1	1	0	1	1
Q13438	Protein OS-9	OS9	1	6	6	4	0	0	2	1	3	1	1	0	0	0	0	2	1	3	1	1	0	0	0	0	2	1	3	1	1	0
Q9ULZ3	Apoptosis-associated speck-like protein containing a CARD	PYCARD	1	7	7	4	0	0	2	2	2	1	1	0	0	0	0	2	2	2	1	1	0	0	0	0	2	2	2	1	1	0
P07108	Acyl-CoA-binding protein	DBI	1	4	4	2	3	1	3	3	1	0	1	1	2	3	1	3	3	1	0	1	1	3	3	1	3	3	1	0	1	1
Q92747	Actin-related protein 2/3 complex subunit 1A	ARPC1A	1	5	4	1	2	2	4	2	1	2	2	1	0	1	1	4	2	1	1	1	0	0	1	1	4	2	1	1	1	0
Q6ZVX7	F-box only protein 50	NCCRP1	1	6	6	4	0	0	2	2	3	0	1	1	0	0	0	2	2	3	0	1	1	0	0	0	2	2	3	0	1	1
P26038	Moesin	MSN	1	20	11	15	10	9	7	6	6	4	5	3	8	5	5	2	2	4	1	1	0	9	7	6	2	2	4	1	1	0
P51149	Ras-related protein Rab-7a	RAB7A	1	7	7	4	3	4	2	2	4	1	1	0	3	3	4	2	2	4	1	1	0	3	3	4	2	2	4	1	1	0
P10619	Lysosomal protective protein	CTSA	1	4	4	0	0	0	2	4	2	0	1	1	0	0	0	2	4	2	0	1	1	0	0	0	2	4	2	0	1	1
P49327	Fatty acid synthase	FASN	1	15	15	0	0	0	3	2	3	1	0	1	0	0	0	3	2	3	1	0	1	0	0	0	3	2	3	1	0	1
P61106	Ras-related protein Rab-14	RAB14	1	14	14	4	1	1	2	2	2	1	1	0	1	1	1	2	2	2	1	1	0	1	1	1	4	3	3	1	1	0
P04062	Glucosylceramidase	GBA	1	4	4	0	0	0	4	3	4	1	0	1	0	0	0	4	3	4	1	0	1	0	0	0	4	3	4	1	0	1
Q15084	Protein disulfide-isomerase A6	PDIA6	1	10	10	4	0	0	2	3	4	2	0	0	0	0	0	2	3	4	2	0	0	0	0	0	3	4	5	2	0	0
P05388	60S acidic ribosomal protein P0	RPLP0;RPLP0P6	2	9	9	2	1	3	5	4	4	1	0	1	2	1	3	5	4	4	1	0	1	2	1	3	5	5	4	1	0	1
P40763	Signal transducer and activator of transcription 3	STAT3	1	7	7	4	0	0	6	4	5	1	0	1	0	0	0	6	4	5	1	0	1	0	0	0	6	4	5	1	0	1
P22531	Small proline-rich protein 2E	SPRR2E	1	5	0	4	1	1	4	4	4	0	1	1	0	0	0	0	0	0	0	0	0	1	1	1	6	6	6	0	1	1
Q08554	Desmocollin-1	DSC1	1	16	16	0	0	0	13	14	13	1	1	0	0	0	0	13	14	13	1	1	0	0	0	0	17	18	16	1	1	0
Q92817	Envoplakin	EVPL	2	38	38	1	2	0	27	25	26	1	0	1	1	2	0	27	25	26	1	0	1	1	2	0	30	28	29	1	0	1
Q96C23	Aldose 1-epimerase	GALM	1	14	14	7	9	8	0	0	0	0	0	1	7	9	8	0	0	0	0	0	1	8	9	8	0	0	0	0	0	1
Q86X76	Nitrilase homolog 1	NIT1	1	8	8	4	5	5	0	0	0	0	0	1	5	5	5	0	0	0	0	0	1	5	5	6	0	0	0	0	0	1
P42574	Caspase-3	CASP3	1	8	8	5	4	5	0	0	0	0	1	0	5	4	5	0	0	0	0	1	0	5	4	5	0	0	0	0	1	0
P02144	Myoglobin	MB	1	7	7	2	4	2	0	0	0	0	1	0	2	4	2	0	0	0	0	1	0	2	5	2	0	0	0	0	1	0
P03952	Plasma kallikrein	KLKB1	1	6	5	3	3	2	0	0	0	1	0	0	2	2	2	0	0	0	0	0	0	3	3	2	0	0	0	1	0	0
P62993	Growth factor receptor-bound protein 2	GRB2	1	5	5	1	3	3	0	0	0	0	0	1	1	3	3	0	0	0	0	0	1	1	3	3	0	0	0	0	0	1
Q9H910	Hematological and neurological expressed 1-like protein	HN1L	1	4	4	3	3	1	0	0	0	1	0	0	3	3	1	0	0	0	1	0	0	3	3	1	0	0	0	1	0	0
P53041	Serine/threonine-protein phosphatase 5	PPP5C	1	3	3	4	1	3	0	0	0	1	0	0	2	1	3	0	0	0	1	0	0	2	1	3	0	0	0	1	0	0
O60936	Nucleolar protein 3	NOL3	2	5	5	2	1	2	0	0	0	0	0	1	2	1	2	0	0	0	0	0	1	2	1	2	0	0	0	0	0	1
Q9P1F3	Costars family protein ABRACL	ABRACL	1	5	5	2	1	2	0	0	0	0	0	1	2	1	2	0	0	0	0	0	1	2	1	2	0	0	0	0	0	1
P98095	Fibulin-2	FBLN2	1	4	4	2	1	2	0	0	0	1	0	0	2	1	2	0	0	0	1	0	0	2	1	2	0	0	0	1	0	0
Q9NZT2	Opioid growth factor receptor	OGFR	1	3	3	4	2	1	0	0	0	0	1	0	2	2	1	0	0	0	0	1	0	2	2	1	0	0	0	0	1	0
P15121	Aldose reductase	AKR1B1	1	4	4	1	1	2	0	0	0	0	0	1	1	1	2	0	0	0	0	0	1	1	1	2	0	0	0	0	0	1
Q92688	Acidic leucine-rich nuclear phosphoprotein 32 family member B	ANP32B	1	4	3	3	2	2	0	0	0	1	0	0	2	1	1	0	0	0	1	0	0	2	1	1	0	0	0	1	0	0
O75936	Gamma-butyrobetaine dioxygenase	BBOX1	1	9	9	0	2	2	0	0	0	1	0	0	0	2	2	0	0	0	1	0	0	0	2	2	0	0	0	1	0	0
Q9H8Y8	Golgi reassembly-stacking protein 2	GORASP2	1	7	7	1	1	2	0	0	0	0	0	1	1	1	2	0	0	0	0	0	1	1	1	2	0	0	0	0	0	1
P15170	Eukaryotic peptide chain release factor GTP-binding subunit ERF3A	GSPT1;GSPT2	2	12	12	1	1	1	0	0	0	1	0	0	1	1	1	0	0	0	1	0	0	1	1	1	0	0	0	1	0	0
P49407	Beta-arrestin-1	ARRB1	1	7	7	1	1	1	0	0	0	0	0	1	1	1	1	0	0	0	0	0	1	1	1	1	0	0	0	0	0	1
Q9NWV8	BRISC and BRCA1-A complex member 1	BABAM1	1	1	1	1	1	1	0	0	0	0	0	1	1	1	1	0	0	0	0	0	1	1	1	1	0	0	0	0	0	1
Q9BY43	Charged multivesicular body protein 4a	CHMP4A	1	1	1	1	1	1	0	0	0	0	1	0	1	1	1	0	0	0	0	1	0	1	1	1	0	0	0	0	1	0
P28161	Glutathione S-transferase Mu 2	GSTM2	1	2	1	1	2	1	0	0	0	0	1	0	1	1	1	0	0	0	0	1	0	1	1	1	0	0	0	0	1	0
P12268	Inosine-5-monophosphate dehydrogenase 2	IMPDH2	1	4	4	1	1	1	0	0	0	0	1	0	1	1	1	0	0	0	0	1	0	1	1	1	0	0	0	0	1	0

O14907	Tax1-binding protein 3	TAX1BP3	1	4	4	4	2	2	1	0	0	0	0	1	2	2	2	1	0	0	0	0	1	2	2	2	1	0	0	0	0	1
P55196	Afadin	MLLT4	1	3	3	2	1	1	1	0	0	0	0	1	2	1	1	1	0	0	0	0	1	2	1	1	1	0	0	0	0	1
P02652	Apolipoprotein A-II	APOA2	1	7	7	1	1	1	0	0	1	0	1	0	1	1	1	0	0	1	0	1	0	1	1	1	0	0	1	0	1	0
O60763	General vesicular transport factor p115	USO1	1	6	6	4	0	1	0	0	1	0	1	0	1	0	1	0	0	1	0	1	0	1	0	1	0	0	1	0	1	0
Q9P289	Serine/threonine-protein kinase 26	STK26;STK25	2	6	4	1	0	0	0	1	0	1	0	0	1	0	0	0	0	0	1	0	0	1	0	0	0	1	0	1	0	0
P61019	Ras-related protein Rab-2A	RAB2A;RAB2B	2	13	13	1	0	0	1	0	0	0	1	0	1	0	0	1	0	0	0	1	0	1	0	0	1	0	0	0	1	0
Q9UBF2	Coatomer subunit gamma-2	COPG2	1	7	5	0	0	1	1	0	0	0	0	1	0	0	1	1	0	0	0	0	1	0	0	1	1	0	0	0	0	1
P07948	Tyrosine-protein kinase Lyn	LYN	1	5	5	0	0	1	0	0	1	0	0	1	0	0	1	0	0	1	0	0	1	0	0	1	0	0	1	0	0	1
P28072	Proteasome subunit beta type-6	PSMB6	1	2	2	4	0	0	1	0	0	0	1	0	1	0	0	1	0	0	0	1	0	1	0	0	1	0	0	0	1	0
P62837	Ubiquitin-conjugating enzyme E2 D2	UBE2D2/D3	4	3	3	0	0	0	0	1	0	0	0	1	0	0	0	0	1	0	0	0	1	0	0	0	0	1	0	0	0	1
Q9UI12	V-type proton ATPase subunit H	ATP6V1H	1	6	6	0	0	0	1	0	0	0	1	0	0	0	0	1	0	0	0	1	0	0	0	0	1	0	0	0	1	0
Q9Y259	Choline/ethanolamine kinase	CHKB	1	1	1	0	0	0	0	1	0	1	0	0	0	0	0	0	1	0	1	0	0	0	0	0	0	1	0	1	0	0
P51665	26S proteasome non-ATPase regulatory subunit 7	PSMD7	1	7	7	4	0	0	0	0	1	1	0	0	0	0	0	0	0	1	1	0	0	0	0	0	0	0	1	1	0	0
P31153	S-adenosylmethionine synthase isoform type-2	MAT2A	2	10	10	9	5	5	1	1	0	0	1	0	9	5	5	1	1	0	0	1	0	9	5	6	1	1	0	0	1	0
Q13442	28 kDa heat- and acid-stable phosphoprotein	PDAP1	1	4	4	4	2	3	0	1	1	0	1	0	3	2	3	0	1	1	0	1	0	3	2	3	0	1	1	0	1	0
P33241	Lymphocyte-specific protein 1	LSP1	1	4	4	2	2	2	0	0	2	0	1	0	2	2	2	0	0	2	0	1	0	2	2	2	0	0	2	0	1	0
P51993	Alpha-(1,3)-fucosyltransferase 6	FUT6;FUT3;FUT5	3	4	4	2	1	1	1	0	1	0	1	0	2	1	1	1	0	1	0	1	0	2	1	1	1	0	1	0	1	0
Q8NFT8	Delta and Notch-like epidermal growth factor-related receptor	DNER	1	1	1	1	1	1	1	1	0	0	0	1	1	1	1	1	1	0	0	0	1	1	1	1	1	1	0	0	0	1
P13929	Beta-enolase	ENO3	1	5	2	3	4	3	2	4	4	2	3	2	0	1	0	0	1	1	0	1	0	0	1	0	0	1	1	0	1	0
A0A075B7B8	IGv domain-containing protein	IGHV3OR16-12	1	2	1	2	1	1	1	2	2	1	2	1	1	0	0	0	1	1	0	1	0	1	0	0	0	1	1	0	1	0
Q9NZR2	Low-density lipoprotein receptor-related protein 1B	LRP1B	1	2	2	0	0	1	1	0	1	0	1	0	0	0	1	1	0	1	0	1	0	0	0	1	1	0	1	0	1	0
Q96L46	Calpain small subunit 2	CAPNS2	1	4	3	0	0	0	1	0	1	1	0	0	0	0	0	1	0	1	1	0	0	0	0	0	1	0	1	1	0	0
Q96RT1	Protein LAP2	ERBB2IP	1	1	1	0	0	0	0	1	1	0	0	1	0	0	0	0	1	1	0	0	1	0	0	0	0	1	1	0	0	1
Q12841	Follistatin-related protein 1	FSTL1	1	2	2	0	0	0	1	1	0	1	0	0	0	0	0	1	1	0	1	0	0	0	0	0	1	1	0	1	0	0
P04085	Platelet-derived growth factor subunit A	PDGFA	1	1	1	4	0	0	1	1	0	0	1	0	0	0	0	1	1	0	0	1	0	0	0	0	1	1	0	0	1	0
P04040	Catalase	CAT	1	6	6	2	2	2	0	1	2	0	1	0	2	2	2	0	1	2	0	1	0	3	3	3	0	1	2	0	1	0
P12081	Histidine--tRNA ligase, cytoplasmic	HARS	2	7	7	1	4	3	1	1	1	1	0	0	1	4	3	1	1	1	1	0	0	1	4	3	1	1	1	1	0	0
P62753	40S ribosomal protein S6	RPS6	1	5	5	4	2	2	1	1	1	1	0	0	2	2	2	1	1	1	1	0	0	2	2	2	1	1	1	1	0	0
Q31612	HLA class I histocompatibility antigen, B-73 alpha chain	HLA-B	1	7	4	1	3	2	1	1	1	0	0	1	0	1	1	0	0	0	0	0	1	0	2	1	1	1	1	0	0	1
P47914	60S ribosomal protein L29	RPL29	1	1	1	4	1	1	1	1	1	1	0	0	0	1	1	1	1	1	1	0	0	0	1	1	1	1	1	1	0	0
P01701	Ig lambda chain V-I region NEW	IGKV1-51	1	1	1	4	1	1	1	1	1	1	0	0	0	1	1	1	1	1	1	0	0	0	1	1	1	1	1	1	0	0
Q12797	Aspartyl/asparaginyl beta-hydroxylase	ASPH	1	12	12	0	1	0	1	1	1	0	0	1	0	1	0	1	1	1	0	0	1	0	1	0	1	1	1	0	0	1
P61966	AP-1 complex subunit sigma-1A	AP1S1	1	1	1	0	0	0	1	1	1	1	0	0	0	0	0	1	1	1	1	0	0	0	0	0	1	1	1	1	0	0
P16278	Beta-galactosidase	GLB1	1	2	2	0	0	0	1	1	1	1	0	0	0	0	0	1	1	1	1	0	0	0	0	0	1	1	1	1	0	0
P02750	Leucine-rich alpha-2-glycoprotein	LRG1	1	11	11	0	0	0	1	2	0	0	1	0	0	0	0	1	2	0	0	1	0	0	0	0	1	2	0	0	1	0
Q96DR8	Mucin-like protein 1	MUCL1	1	1	1	4	0	0	1	1	1	0	0	1	0	0	0	1	1	1	0	0	1	0	0	0	1	1	1	0	0	1
P07437	Tubulin beta chain	TUBB	1	23	5	4	3	3	9	8	8	11	10	10	0	0	0	1	1	1	1	0	0	0	0	0	1	1	1	1	0	0
P26640	Valine--tRNA ligase	VARS	1	6	6	4	0	0	0	1	2	1	0	0	0	0	0	0	1	2	1	0	0	0	0	0	0	1	2	1	0	0
P62714	Serine/threonine-protein phosphatase 2A catalytic subunit beta isoform	PPP2CB	1	9	1	4	7	5	1	2	1	1	0	0	0	1	1	0	1	0	0	0	0	7	7	7	1	2	1	1	0	0
P49591	Serine--tRNA ligase, cytoplasmic	SARS	1	8	8	4	0	2	2	0	2	0	1	0	2	0	2	2	0	2	0	1	0	2	0	2	2	0	2	0	1	0
P62917	60S ribosomal protein L8	RPL8	1	2	2	4	1	1	1	1	1	0	0	1	1	1	1	1	1	1	0	0	1	1	1	1	1	2	1	0	0	1
P49419	Alpha-aminoadipic semialdehyde dehydrogenase	ALDH7A1	1	12	12	1	0	0	2	1	1	0	0	1	1	0	0	2	1	1	0	0	1	1	0	0	2	1	1	0	0	1
P62191	26S protease regulatory subunit 4	PSMC1	1	5	5	4	0	0	2	1	1	0	1	0	0	0	0	2	1	1	0	1	0	0	0	0	2	1	1	0	1	0
P31943	Heterogeneous nuclear ribonucleoprotein H	HNRNPH1	2	5	3	2	3	1	3	3	4	1	2	1	1	2	0	1	2	2	0	1	0	1	2	0	1	2	2	0	1	0
Q9BQE3	Tubulin alpha-1C chain	TUBA1C	1	19	3	4	4	5	11	10	9	8	8	8	0	0	0	1	0	1	0	0	0	1	0	1	2	1	2	0	1	0
Q04637	Eukaryotic translation initiation factor 4 gamma 1	EIF4G1	1	7	7	0	0	0	1	2	2	0	0	1	0	0	0	1	2	2	0	0	1	0	0	0	1	2	2	0	0	1
Q7Z7M9	Polypeptide N-acetylgalactosaminyltransferase 5	GALNT5	1	9	9	0	0	0	2	2	1	0	1	0	0	0	0	2	2	1	0	1	0	0	0	0	2	2	1	0	1	0
Q99954	Submaxillary gland androgen-regulated protein 3A	SMR3A	1	1	1	4	1	1	1	1	1	1	0	0	1	1	1	1	1	1	1	0	0	2	1	1	3	2	1	1	0	0
P46783	40S ribosomal protein S10	RPS10;RPS10P5	2	3	3	0	1	2	2	3	1	0	0	1	0	1	2	2	3	1	0	0	1	0	1	2	2	3	1	0	0	1
Q96FQ6	Protein S100-A16	S100A16	1	4	4	4	0	1	2	2	2	0	0	1	1	0	1	2	2	2	0	0	1	1	0	1	2	2	2	0	0	1

O00182	Galectin-9	LGALS9	1	6	4	1	1	1	0	0	0	1	0	0	1	1	1	0	0	0	1	0	0	1	1	1	0	0	0	1	0	0
Q9BV36	Melanophilin	MLPH	1	2	2	1	1	1	0	0	0	1	0	0	1	1	1	0	0	0	1	0	0	1	1	1	0	0	0	1	0	0
P41218	Myeloid cell nuclear differentiation antigen	MNDA	1	10	10	1	1	1	0	0	0	0	1	0	1	1	1	0	0	0	0	1	0	1	1	1	0	0	0	0	1	0
Q9Y617	Phosphoserine aminotransferase	PSAT1	1	2	2	4	1	1	0	0	0	1	0	0	1	1	1	0	0	0	1	0	0	1	1	1	0	0	0	1	0	0
Q01082	Spectrin beta chain, non-erythrocytic 1	SPTBN1	1	16	16	4	1	1	0	0	0	0	0	1	1	1	1	0	0	0	0	0	1	1	1	1	0	0	0	0	0	1
Q8TE77	Protein phosphatase Slingshot homolog 3	SSH3	1	8	8	4	1	1	0	0	0	0	0	1	1	1	1	0	0	0	0	0	1	1	1	1	0	0	0	0	0	1
Q9Y2B0	Protein canopy homolog 2	CNPY2	1	5	5	1	1	0	0	0	0	0	0	1	1	1	0	0	0	0	0	0	1	1	1	0	0	0	0	0	0	1
Q8N1G4	Leucine-rich repeat-containing protein 47	LRRC47	1	5	5	1	1	0	0	0	0	0	1	0	1	1	0	0	0	0	0	1	0	1	1	0	0	0	0	0	1	0
P21741	Midkine	MDK	1	2	2	1	0	1	0	0	0	0	0	1	1	0	1	0	0	0	0	0	1	1	0	1	0	0	0	0	0	1
Q99471	Prefoldin subunit 5	PFDN5	1	4	4	4	0	1	0	0	0	0	1	0	1	0	1	0	0	0	0	1	0	1	0	1	0	0	0	0	1	0
Q15257	Serine/threonine-protein phosphatase 2A activator	PPP2R4	1	5	5	4	1	0	0	0	0	0	1	0	1	1	0	0	0	0	0	1	0	1	1	0	0	0	0	0	1	0
P68036	Ubiquitin-conjugating enzyme E2 L3	UBE2L3	2	8	8	0	0	1	0	0	0	0	0	1	0	0	1	0	0	0	0	0	1	0	0	1	0	0	0	0	0	1
Q7Z4W1	L-xylulose reductase	DCXR	1	4	4	1	0	0	0	0	0	0	1	0	1	0	0	0	0	0	0	1	0	1	0	0	0	0	0	0	1	0
Q9H4G4	Golgi-associated plant pathogenesis-related protein 1	GLIPR2	1	2	2	0	1	0	0	0	0	0	0	1	0	1	0	0	0	0	0	0	1	0	1	0	0	0	0	0	0	1
Q8NC56	LEM domain-containing protein 2	LEMD2	1	2	2	0	1	0	0	0	0	0	0	1	0	1	0	0	0	0	0	0	1	0	1	0	0	0	0	0	0	1
Q92597	Protein NDRG1	NDRG1	1	5	5	4	0	1	0	0	0	0	1	0	0	0	1	0	0	0	0	1	0	0	0	1	0	0	0	0	1	0
P0DMN0	Sulfotransferase 1A4	SULT1A4/A3	2	4	2	0	0	0	0	0	0	0	1	0	0	0	0	0	0	0	0	1	0	0	0	0	0	0	0	0	1	0
P47893	Olfactory receptor 3A2	OR3A2;OR3A3	2	1	1	0	0	0	0	0	0	0	0	1	0	0	0	0	0	0	0	0	1	0	0	0	0	0	0	0	0	1
P53396	ATP-citrate synthase	ACLY	1	6	6	0	0	0	0	0	0	0	0	1	0	0	0	0	0	0	0	0	1	0	0	0	0	0	0	0	0	1
Q13443	Disintegrin and metalloproteinase domain-containing protein 9	ADAM9	1	1	1	0	0	0	0	0	0	1	0	0	0	0	0	0	0	0	1	0	0	0	0	0	0	0	0	1	0	0
Q9NX46	Poly(ADP-ribose) glycohydrolase ARH3	ADPRHL2	1	1	1	0	0	0	0	0	0	0	0	1	0	0	0	0	0	0	0	0	1	0	0	0	0	0	0	0	0	1
P55008	Allograft inflammatory factor 1	AIF1	1	2	2	0	0	0	0	0	0	0	0	1	0	0	0	0	0	0	0	0	1	0	0	0	0	0	0	0	0	1
Q9H3K6	BolA-like protein 2	BOLA2	1	1	1	0	0	0	0	0	0	0	0	1	0	0	0	0	0	0	0	0	1	0	0	0	0	0	0	0	0	1
Q9GZT6	Coiled-coil domain-containing protein 90B, mitochondrial	CCDC90B	1	1	1	0	0	0	0	0	0	1	0	0	0	0	0	0	0	0	1	0	0	0	0	0	0	0	0	1	0	0
P14868	Aspartate--tRNA ligase, cytoplasmic	DARS	1	5	5	0	0	0	0	0	0	1	0	0	0	0	0	0	0	0	1	0	0	0	0	0	0	0	0	1	0	0
Q9UMR2	ATP-dependent RNA helicase DDX19B	DDX19B	1	8	2	0	0	0	0	0	0	1	0	0	0	0	0	0	0	0	0	0	0	0	0	0	0	0	0	1	0	0
P27487	Dipeptidyl peptidase 4	DPP4	1	1	1	0	0	0	0	0	0	0	0	1	0	0	0	0	0	0	0	0	1	0	0	0	0	0	0	0	0	1
Q05639	Elongation factor 1-alpha 2	EEF1A2	1	11	3	2	2	3	2	3	2	3	2	3	0	0	0	0	0	0	1	0	0	0	0	0	0	0	0	1	0	0
P01133	Pro-epidermal growth factor	EGF	1	2	2	0	0	0	0	0	0	0	0	1	0	0	0	0	0	0	0	0	1	0	0	0	0	0	0	0	0	1
Q14240	Eukaryotic initiation factor 4A-II	EIF4A2	1	8	3	0	0	0	3	3	2	3	3	2	0	0	0	0	0	0	1	0	0	0	0	0	0	0	0	1	0	0
Q96MK3	Protein FAM20A	FAM20A	1	2	2	0	0	0	0	0	0	0	0	1	0	0	0	0	0	0	0	0	1	0	0	0	0	0	0	0	0	1
Q9NUQ9	Protein FAM49B	FAM49B	1	2	2	0	0	0	0	0	0	1	0	0	0	0	0	0	0	0	1	0	0	0	0	0	0	0	0	1	0	0
Q7Z4H3	HD domain-containing protein 2	HDDC2	1	2	2	0	0	0	0	0	0	0	1	0	0	0	0	0	0	0	0	1	0	0	0	0	0	0	0	0	1	0
P13284	Gamma-interferon-inducible lysosomal thiol reductase	IFI30	1	2	2	0	0	0	0	0	0	1	0	0	0	0	0	0	0	0	1	0	0	0	0	0	0	0	0	1	0	0
A0A0C4DH29	Immunoglobulin heavy variable 1-3	IGHV1-3	1	2	1	1	1	0	1	1	1	2	1	1	0	0	0	0	0	0	1	0	0	0	0	0	0	0	0	1	0	0
Q9H2U6	Putative uncharacterized protein encoded by LINC00597	LINC00597	1	1	1	0	0	0	0	0	0	1	0	0	0	0	0	0	0	0	1	0	0	0	0	0	0	0	0	1	0	0
Q9H8J5	MANSC domain-containing protein 1	MANSC1	1	1	1	0	0	0	0	0	0	1	0	0	0	0	0	0	0	0	1	0	0	0	0	0	0	0	0	1	0	0
Q8TD08	Mitogen-activated protein kinase 15	MAPK15	1	1	1	0	0	0	0	0	0	1	0	0	0	0	0	0	0	0	1	0	0	0	0	0	0	0	0	1	0	0
Q8WXI7	Mucin-16	MUC16	1	4	4	0	0	0	0	0	0	0	0	1	0	0	0	0	0	0	0	0	1	0	0	0	0	0	0	0	0	1
O95747	Serine/threonine-protein kinase OSR1	OXSR1	1	3	3	4	0	0	0	0	0	0	1	0	0	0	0	0	0	0	0	1	0	0	0	0	0	0	0	0	1	0
P49903	Selenide, water dikinase 1	SEPHS1	1	1	1	4	0	0	0	0	0	0	1	0	0	0	0	0	0	0	0	1	0	0	0	0	0	0	0	0	1	0
Q9UHD8	Septin-9	SEPTIN9	1	6	6	4	0	0	0	0	0	0	0	1	0	0	0	0	0	0	0	0	1	0	0	0	0	0	0	0	0	1
P52788	Spermine synthase	SMS	1	7	7	4	0	0	0	0	0	0	1	0	0	0	0	0	0	0	0	1	0	0	0	0	0	0	0	0	1	0
Q7L7X3	Serine/threonine-protein kinase TAO1	TAOK1	1	1	1	4	0	0	0	0	0	1	0	0	0	0	0	0	0	0	1	0	0	0	0	0	0	0	0	1	0	0
Q9NQ88	Fructose-2,6-bisphosphatase TIGAR	TIGAR	1	1	1	4	0	0	0	0	0	1	0	0	0	0	0	0	0	0	1	0	0	0	0	0	0	0	0	1	0	0
Q15819	Ubiquitin-conjugating enzyme E2 variant 2	UBE2V2	1	5	2	4	0	0	0	0	0	1	1	1	0	0	0	0	0	0	0	0	1	0	0	0	0	0	0	0	0	1
Q99798	Aconitate hydratase, mitochondrial	ACO2	1	16	16	4	6	4	1	0	0	0	0	1	4	6	4	1	0	0	0	0	1	4	6	4	1	0	0	0	0	1
P41567	Eukaryotic translation initiation factor 1	EIF1;EIF1B	2	6	6	4	2	2	1	0	0	0	1	0	4	2	2	1	0	0	0	1	0	4	2	2	1	0	0	0	1	0
P61313	60S ribosomal protein L15	RPL15	1	3	3	4	2	3	0	1	0	0	1	0	3	2	3	0	1	0	0	1	0	3	2	3	0	1	0	0	1	0
P02753	Retinol-binding protein 4	RBP4	1	10	10	4	2	2	0	1	0	0	1	0	3	2	2	0	1	0	0	1	0	3	2	2	0	1	0	0	1	0

Q16270	Insulin-like growth factor-binding protein 7	IGFBP7	1	2	2	1	0	0	2	2	2	0	0	1	1	0	0	2	2	2	0	0	1	1	0	0	2	2	2	0	0	1
P41091	Eukaryotic translation initiation factor 2 subunit 3	EIF2S3;EIF2S3L	2	9	9	0	0	0	2	2	2	0	0	1	0	0	0	2	2	2	0	0	1	0	0	0	2	2	2	0	0	1
P21796	Voltage-dependent anion-selective channel protein 1	VDAC1	1	12	12	4	0	0	2	2	3	1	0	0	0	0	0	2	2	3	1	0	0	0	0	0	2	2	3	1	0	0
P62081	40S ribosomal protein S7	RPS7	1	6	6	4	0	0	4	1	4	0	0	1	0	0	0	4	1	4	0	0	1	0	0	0	4	1	4	0	0	1
P52272	Heterogeneous nuclear ribonucleoprotein M	HNRNPM	1	9	9	2	2	2	3	5	3	0	0	1	2	2	2	3	5	3	0	0	1	2	2	2	3	5	3	0	0	1
Q14247	Src substrate cortactin	CTTN	1	14	14	10	6	7	4	4	4	0	0	1	10	6	7	4	4	4	0	0	1	10	6	7	4	4	4	0	0	1
P35221	Catenin alpha-1	CTNNA1	1	21	16	0	0	0	4	3	7	0	0	1	0	0	0	3	2	6	0	0	1	0	0	0	4	3	8	0	0	1
P30050	60S ribosomal protein L12	RPL12	1	7	7	4	1	0	5	4	4	1	0	0	1	1	0	5	4	4	1	0	0	1	1	0	7	6	5	1	0	0
Q86SG5	Protein S100-A7A	S100A7A	1	11	7	4	2	2	10	10	10	2	3	4	0	0	0	7	6	6	0	0	1	0	0	0	9	7	6	0	0	1
P21333	Filamin-A	FLNA	1	16	15	1	2	1	8	8	8	1	0	0	0	1	0	8	8	8	1	0	0	0	1	0	8	8	8	1	0	0
Q5VT79	Annexin A8-like protein 2	ANXA8L2	1	9	1	1	1	1	7	7	7	0	1	0	0	0	0	1	1	1	0	0	0	1	1	1	9	8	8	0	1	0
P15924	Desmoplakin	DSP	1	111	111	0	1	0	87	87	87	1	0	0	0	1	0	87	87	87	1	0	0	0	1	0	96	97	98	1	0	0
P05023	Sodium/potassium-transporting ATPase subunit alpha-1	ATP1A1	3	28	22	6	7	7	0	0	0	0	0	0	4	4	5	0	0	0	0	0	0	6	7	7	0	0	0	0	0	0
Q16881	Thioredoxin reductase 1, cytoplasmic	TXNRD1	1	12	12	4	6	8	0	0	0	0	0	0	3	6	8	0	0	0	0	0	0	4	7	9	0	0	0	0	0	0
P09417	Dihydropteridine reductase	QDPR	1	8	8	4	5	5	0	0	0	0	0	0	7	5	5	0	0	0	0	0	0	7	6	5	0	0	0	0	0	0
P27695	DNA-(apurinic or apyrimidinic site) lyase	APEX1	1	8	8	6	4	7	0	0	0	0	0	0	6	4	7	0	0	0	0	0	0	6	4	7	0	0	0	0	0	0
P13716	Delta-aminolevulinic acid dehydratase	ALAD	1	9	9	5	6	5	0	0	0	0	0	0	5	6	5	0	0	0	0	0	0	5	6	5	0	0	0	0	0	0
O75531	Barrier-to-autointegration factor	BANF1	1	6	6	5	4	4	0	0	0	0	0	0	5	4	4	0	0	0	0	0	0	6	5	4	0	0	0	0	0	0
P35241	Radixin	RDX	1	21	11	4	11	9	6	4	3	4	5	4	4	5	5	0	0	0	0	0	0	4	5	5	0	0	0	0	0	0
Q9UBQ0	Vacuolar protein sorting-associated protein 29	VPS29	1	4	4	4	3	3	0	0	0	0	0	0	4	3	3	0	0	0	0	0	0	5	4	4	0	0	0	0	0	0
Q32MZ4	Leucine-rich repeat flightless-interacting protein 1	LRRFIP1	1	8	8	5	2	4	0	0	0	0	0	0	5	2	4	0	0	0	0	0	0	5	3	4	0	0	0	0	0	0
Q9NVS9	Pyridoxine-5-phosphate oxidase	PNPO	1	8	8	4	4	4	0	0	0	0	0	0	4	4	4	0	0	0	0	0	0	4	4	4	0	0	0	0	0	0
Q8N1Q1	Carbonic anhydrase 13	CA13	1	5	5	3	3	5	0	0	0	0	0	0	3	3	5	0	0	0	0	0	0	3	3	5	0	0	0	0	0	0
P46108	Adapter molecule crk	CRK	1	9	9	4	3	4	0	0	0	0	0	0	4	3	4	0	0	0	0	0	0	4	3	4	0	0	0	0	0	0
Q8NDH3	Probable aminopeptidase NPEPL1	NPEPL1	1	9	9	4	3	3	0	0	0	0	0	0	4	3	3	0	0	0	0	0	0	5	3	3	0	0	0	0	0	0
Q9GZT8	NIF3-like protein 1	NIF3L1	1	5	5	4	3	2	0	0	0	0	0	0	4	3	2	0	0	0	0	0	0	4	4	2	0	0	0	0	0	0
O15212	Prefoldin subunit 6	PFDN6	1	5	5	4	3	4	0	0	0	0	0	0	3	3	4	0	0	0	0	0	0	3	3	4	0	0	0	0	0	0
P05455	Lupus La protein	SSB	1	9	9	4	3	3	0	0	0	0	0	0	4	3	3	0	0	0	0	0	0	4	3	3	0	0	0	0	0	0
Q9BV57	1,2-dihydroxy-3-keto-5-methylthiopentene dioxygenase	ADI1	1	4	4	3	4	2	0	0	0	0	0	0	3	4	2	0	0	0	0	0	0	3	4	2	0	0	0	0	0	0
Q99102	Mucin-4	MUC4	1	5	5	4	2	3	0	0	0	0	0	0	4	2	3	0	0	0	0	0	0	4	2	3	0	0	0	0	0	0
Q5SRE7	Phytanoyl-CoA dioxygenase domain-containing protein 1	PHYHD1	1	5	4	4	3	3	0	0	0	0	0	0	3	3	3	0	0	0	0	0	0	3	3	3	0	0	0	0	0	0
Q9UHJ6	Sedoheptulokinase	SHPK	1	4	4	4	2	3	0	0	0	0	0	0	4	2	3	0	0	0	0	0	0	4	2	3	0	0	0	0	0	0
P00367	Glutamate dehydrogenase 1, mitochondrial	GLUD1;GLUD2	2	16	16	3	2	3	0	0	0	0	0	0	3	2	3	0	0	0	0	0	0	3	2	3	0	0	0	0	0	0
Q9NP97	Dynein light chain roadblock-type 1	DYNLRB1;DYNLRB2	2	5	5	3	2	3	0	0	0	0	0	0	3	2	3	0	0	0	0	0	0	3	2	3	0	0	0	0	0	0
P08133	Annexin A6	ANXA6	1	5	5	3	2	3	0	0	0	0	0	0	3	2	3	0	0	0	0	0	0	3	2	3	0	0	0	0	0	0
Q9NWV4	UPF0587 protein C1orf123	C1orf123	1	4	4	4	2	2	0	0	0	0	0	0	4	2	2	0	0	0	0	0	0	4	2	2	0	0	0	0	0	0
P35219	Carbonic anhydrase-related protein	CA8	1	4	4	3	2	3	0	0	0	0	0	0	3	2	3	0	0	0	0	0	0	3	2	3	0	0	0	0	0	0
Q13642	Four and a half LIM domains protein 1	FHL1	1	4	4	2	3	3	0	0	0	0	0	0	2	3	3	0	0	0	0	0	0	2	3	3	0	0	0	0	0	0
O75594	Peptidoglycan recognition protein 1	PGLYRP1	1	3	3	4	2	3	0	0	0	0	0	0	3	2	3	0	0	0	0	0	0	3	2	3	0	0	0	0	0	0
P09341	Growth-regulated alpha protein	CXCL1/3/2	3	2	2	2	2	2	0	0	0	0	0	0	2	2	2	0	0	0	0	0	0	3	2	2	0	0	0	0	0	0
Q6NXS1	Protein phosphatase inhibitor 2-like protein 3	PPP1R2P3;PPP1R2	2	2	2	2	2	2	0	0	0	0	0	0	2	2	2	0	0	0	0	0	0	3	2	2	0	0	0	0	0	0
Q03154	Aminoacylase-1	ACY1	1	5	5	4	2	1	0	0	0	0	0	0	4	2	1	0	0	0	0	0	0	4	2	1	0	0	0	0	0	0
P35573	Glycogen debranching enzyme	AGL	1	9	9	3	2	2	0	0	0	0	0	0	3	2	2	0	0	0	0	0	0	3	2	2	0	0	0	0	0	0
P49354	Protein farnesyltransferase/geranylgeranyltransferase type-1 subunit alpha	FNTA	1	4	4	3	1	3	0	0	0	0	0	0	3	1	3	0	0	0	0	0	0	3	1	3	0	0	0	0	0	0
P30047	GTP cyclohydrolase 1 feedback regulatory protein	GCHFR	1	4	4	1	2	1	0	0	0	0	0	0	1	2	1	0	0	0	0	0	0	2	3	2	0	0	0	0	0	0
Q9BW91	ADP-ribose pyrophosphatase, mitochondrial	NUDT9	1	4	4	4	3	1	0	0	0	0	0	0	3	3	1	0	0	0	0	0	0	3	3	1	0	0	0	0	0	0
O14737	Programmed cell death protein 5	PDCD5	1	4	4	4	3	3	0	0	0	0	0	0	1	3	3	0	0	0	0	0	0	1	3	3	0	0	0	0	0	0
P50914	60S ribosomal protein L14	RPL14	1	4	4	4	3	2	0	0	0	0	0	0	2	3	2	0	0	0	0	0	0	2	3	2	0	0	0	0	0	0
O75436	Vacuolar protein sorting-associated protein 26A	VPS26A	1	4	4	4	3	3	0	0	0	0	0	0	1	3	3	0	0	0	0	0	0	1	3	3	0	0	0	0	0	0
Q13185	Chromobox protein homolog 3	CBX3	1	4	3	3	1	2	0	0	0	0	0	0	2	1	1	0	0	0	0	0	0	3	1	2	0	0	0	0	0	0

Q14894	Ketimine reductase mu-crystallin	CRYM	1	4	4	2	2	1	0	0	0	0	0	0	2	2	1	0	0	0	0	0	0	2	3	1	0	0	0	0	0	0
Q13409	Cytoplasmic dynein 1 intermediate chain 2	DYNC1I2	1	4	4	2	1	3	0	0	0	0	0	0	2	1	3	0	0	0	0	0	0	2	1	3	0	0	0	0	0	0
P13726	Tissue factor	F3	1	5	5	3	2	1	0	0	0	0	0	0	3	2	1	0	0	0	0	0	0	3	2	1	0	0	0	0	0	0
Q16775	Hydroxyacylglutathione hydrolase, mitochondrial	HAGH	1	2	2	1	2	2	0	0	0	0	0	0	1	2	2	0	0	0	0	0	0	2	2	2	0	0	0	0	0	0
P15941	Mucin-1	MUC1	1	4	4	1	2	3	0	0	0	0	0	0	1	2	3	0	0	0	0	0	0	1	2	3	0	0	0	0	0	0
P61970	Nuclear transport factor 2	NUTF2	1	2	2	4	2	2	0	0	0	0	0	0	2	2	2	0	0	0	0	0	0	2	2	2	0	0	0	0	0	0
Q9UHV9	Prefoldin subunit 2	PFDN2	1	3	3	4	2	2	0	0	0	0	0	0	2	2	2	0	0	0	0	0	0	2	2	2	0	0	0	0	0	0
Q9Y3C6	Peptidyl-prolyl cis-trans isomerase-like 1	PPIL1	1	3	3	4	1	3	0	0	0	0	0	0	2	1	3	0	0	0	0	0	0	2	1	3	0	0	0	0	0	0
P41222	Prostaglandin-H2 D-isomerase	PTGDS	1	2	2	4	2	2	0	0	0	0	0	0	2	2	2	0	0	0	0	0	0	2	2	2	0	0	0	0	0	0
P46778	60S ribosomal protein L21	RPL21	1	2	2	4	1	1	0	0	0	0	0	0	1	1	1	0	0	0	0	0	0	2	2	2	0	0	0	0	0	0
P83731	60S ribosomal protein L24	RPL24	1	2	2	4	2	2	0	0	0	0	0	0	2	2	2	0	0	0	0	0	0	2	2	2	0	0	0	0	0	0
P46779	60S ribosomal protein L28	RPL28	1	3	3	4	1	2	0	0	0	0	0	0	3	1	2	0	0	0	0	0	0	3	1	2	0	0	0	0	0	0
Q15019	Septin-2	SEPTIN2	1	8	8	4	1	3	0	0	0	0	0	0	2	1	3	0	0	0	0	0	0	2	1	3	0	0	0	0	0	0
Q9GZS3	WD repeat-containing protein 61	WDR61	1	4	4	4	2	2	0	0	0	0	0	0	2	2	2	0	0	0	0	0	0	2	2	2	0	0	0	0	0	0
Q8NEV1	Casein kinase II subunit alpha 3	CSNK2A3/A1	2	5	5	0	3	2	0	0	0	0	0	0	0	3	2	0	0	0	0	0	0	0	3	2	0	0	0	0	0	0
Q02878	60S ribosomal protein L6	RPL6	2	7	7	2	1	2	0	0	0	0	0	0	2	1	2	0	0	0	0	0	0	2	1	2	0	0	0	0	0	0
Q9UKY7	Protein CDV3 homolog	CDV3	1	3	3	1	1	1	0	0	0	0	0	0	1	1	1	0	0	0	0	0	0	2	1	2	0	0	0	0	0	0
O76071	Probable cytosolic iron-sulfur protein assembly protein CIAO1	CIAO1	1	2	2	2	2	1	0	0	0	0	0	0	2	2	1	0	0	0	0	0	0	2	2	1	0	0	0	0	0	0
P08311	Cathepsin G	CTSG	1	4	4	3	1	1	0	0	0	0	0	0	3	1	1	0	0	0	0	0	0	3	1	1	0	0	0	0	0	0
Q93099	Homogentisate 1,2-dioxygenase	HGD	1	4	4	1	2	2	0	0	0	0	0	0	1	2	2	0	0	0	0	0	0	1	2	2	0	0	0	0	0	0
Q9NZL9	Methionine adenosyltransferase 2 subunit beta	MAT2B	1	6	6	2	2	1	0	0	0	0	0	0	2	2	1	0	0	0	0	0	0	2	2	1	0	0	0	0	0	0
P80297	Metallothionein-1X	MT1X	1	2	2	2	1	2	0	0	0	0	0	0	2	1	2	0	0	0	0	0	0	2	1	2	0	0	0	0	0	0
O60925	Prefoldin subunit 1	PFDN1	1	3	3	4	2	1	0	0	0	0	0	0	2	2	1	0	0	0	0	0	0	2	2	1	0	0	0	0	0	0
P53609	Geranylgeranyl transferase type-1 subunit beta	PGGT1B	1	3	2	4	2	1	0	0	0	0	0	0	2	2	1	0	0	0	0	0	0	2	2	1	0	0	0	0	0	0
O94903	Proline synthase co-transcribed bacterial homolog protein	PROSC	1	3	3	4	2	1	0	0	0	0	0	0	2	2	1	0	0	0	0	0	0	2	2	1	0	0	0	0	0	0
Q687X5	Metalloreductase STEAP4	STEAP4	1	4	4	4	2	2	0	0	0	0	0	0	1	2	2	0	0	0	0	0	0	1	2	2	0	0	0	0	0	0
Q53FA7	Quinone oxidoreductase PIG3	TP53I3	1	4	4	4	3	1	0	0	0	0	0	0	1	3	1	0	0	0	0	0	0	1	3	1	0	0	0	0	0	0
P06132	Uroporphyrinogen decarboxylase	UROD	1	5	5	4	2	1	0	0	0	0	0	0	2	2	1	0	0	0	0	0	0	2	2	1	0	0	0	0	0	0
P61254	60S ribosomal protein L26	RPL26;RPL26L1	3	3	3	1	1	2	0	0	0	0	0	0	1	1	2	0	0	0	0	0	0	1	1	2	0	0	0	0	0	0
K7EP46	Thimet oligopeptidase	THOP1	2	2	2	2	1	1	0	0	0	0	0	0	2	1	1	0	0	0	0	0	0	2	1	1	0	0	0	0	0	0
P53367	Arfaptin-1	ARFIP1	1	5	5	1	1	2	0	0	0	0	0	0	1	1	2	0	0	0	0	0	0	1	1	2	0	0	0	0	0	0
Q6P6B1	Glutamate-rich protein 5	ERICH5	1	3	3	1	1	2	0	0	0	0	0	0	1	1	2	0	0	0	0	0	0	1	1	2	0	0	0	0	0	0
O43708	Maleylacetoacetate isomerase	GSTZ1	1	4	4	1	2	1	0	0	0	0	0	0	1	2	1	0	0	0	0	0	0	1	2	1	0	0	0	0	0	0
P48735	Isocitrate dehydrogenase [NADP], mitochondrial	IDH2	1	9	9	1	1	2	0	0	0	0	0	0	1	1	2	0	0	0	0	0	0	1	1	2	0	0	0	0	0	0
Q93052	Lipoma-preferred partner	LPP	1	3	3	1	1	2	0	0	0	0	0	0	1	1	2	0	0	0	0	0	0	1	1	2	0	0	0	0	0	0
Q8WZA0	Protein LZIC	LZIC	1	3	3	1	2	1	0	0	0	0	0	0	1	2	1	0	0	0	0	0	0	1	2	1	0	0	0	0	0	0
Q96EY5	Multivesicular body subunit 12A	MVB12A	1	2	2	4	1	2	0	0	0	0	0	0	1	1	2	0	0	0	0	0	0	1	1	2	0	0	0	0	0	0
P02763	Alpha-1-acid glycoprotein 1	ORM1	1	5	5	4	1	1	0	0	0	0	0	0	1	1	1	0	0	0	0	0	0	1	1	2	0	0	0	0	0	0
Q9NQP4	Prefoldin subunit 4	PFDN4	1	3	3	4	1	1	0	0	0	0	0	0	2	1	1	0	0	0	0	0	0	2	1	1	0	0	0	0	0	0
Q13526	Peptidyl-prolyl cis-trans isomerase NIMA-interacting 1	PIN1	1	2	2	4	1	2	0	0	0	0	0	0	1	1	2	0	0	0	0	0	0	1	1	2	0	0	0	0	0	0
P63173	60S ribosomal protein L38	RPL38	1	2	2	4	1	2	0	0	0	0	0	0	1	1	2	0	0	0	0	0	0	1	1	2	0	0	0	0	0	0
Q07654	Trefoil factor 3	TFF3	1	3	3	4	1	2	0	0	0	0	0	0	1	1	2	0	0	0	0	0	0	1	1	2	0	0	0	0	0	0
Q9H3S4	Thiamin pyrophosphokinase 1	TPK1	1	3	2	4	1	1	0	0	0	0	0	0	2	1	1	0	0	0	0	0	0	2	1	1	0	0	0	0	0	0
P10155	60 kDa SS-A/Ro ribonucleoprotein	TROVE2	1	6	6	4	1	1	0	0	0	0	0	0	2	1	1	0	0	0	0	0	0	2	1	1	0	0	0	0	0	0
Q96B54	Zinc finger protein 428	ZNF428	1	2	2	4	1	1	0	0	0	0	0	0	2	1	1	0	0	0	0	0	0	2	1	1	0	0	0	0	0	0
P01709	Ig lambda chain V-II region MGC	IGKV2-8	2	2	2	2	0	1	0	0	0	0	0	0	2	0	1	0	0	0	0	0	0	2	0	1	0	0	0	0	0	0
P0DPI2	Glutamine amidotransferase-like class 1 domain-containing protein 3A, mitochondrial	GATD3A	2	4	4	1	1	1	0	0	0	0	0	0	1	1	1	0	0	0	0	0	0	1	1	1	0	0	0	0	0	0
P62879	Guanine nucleotide-binding protein G(I)/G(S)/G(T) subunit beta-2	GNB2	2	12	7	4	2	2	0	0	0	0	0	0	2	0	1	0	0	0	0	0	0	2	0	1	0	0	0	0	0	0
P62854	40S ribosomal protein S26	RPS26;RPS26P11	2	2	2	2	0	1	0	0	0	0	0	0	2	0	1	0	0	0	0	0	0	2	0	1	0	0	0	0	0	0
P00325	Alcohol dehydrogenase 1B	ADH1B	1	8	1	5	3	4	0	0	0	1	2	2	1	1	0	0	0	0	0	0	0	1	2	0	0	0	0	0	0	0

P52594	Arf-GAP domain and FG repeat-containing protein 1	AGFG1	1	2	2	1	1	1	0	0	0	0	0	0	1	1	1	0	0	0	0	0	0	1	1	1	0	0	0	0	0	0
O94973	AP-2 complex subunit alpha-2	AP2A2	1	9	7	1	1	1	0	0	0	0	0	0	1	1	1	0	0	0	0	0	0	1	1	1	0	0	0	0	0	0
Q96GX9	Methylthioribulose-1-phosphate dehydratase	APIP	1	1	1	1	1	1	0	0	0	0	0	0	1	1	1	0	0	0	0	0	0	1	1	1	0	0	0	0	0	0
P80723	Brain acid soluble protein 1	BASP1	1	3	3	2	1	0	0	0	0	0	0	0	2	1	0	0	0	0	0	0	0	2	1	0	0	0	0	0	0	0
P02746	Complement C1q subcomponent subunit B	C1QB	1	4	4	1	1	1	0	0	0	0	0	0	1	1	1	0	0	0	0	0	0	1	1	1	0	0	0	0	0	0
Q9Y5K6	CD2-associated protein	CD2AP	1	3	3	0	2	1	0	0	0	0	0	0	0	2	1	0	0	0	0	0	0	0	2	1	0	0	0	0	0	0
O00533	Neural cell adhesion molecule L1-like protein	CHL1	1	1	1	1	1	1	0	0	0	0	0	0	1	1	1	0	0	0	0	0	0	1	1	1	0	0	0	0	0	0
P12277	Creatine kinase B-type	CKB	1	2	2	1	1	1	0	0	0	0	0	0	1	1	1	0	0	0	0	0	0	1	1	1	0	0	0	0	0	0
P09496	Clathrin light chain A	CLTA	1	2	2	1	1	1	0	0	0	0	0	0	1	1	1	0	0	0	0	0	0	1	1	1	0	0	0	0	0	0
Q9BT09	Protein canopy homolog 3	CNPY3	1	1	1	1	1	1	0	0	0	0	0	0	1	1	1	0	0	0	0	0	0	1	1	1	0	0	0	0	0	0
Q9ULV4	Coronin-1C	CORO1C	1	4	4	0	1	2	0	0	0	0	0	0	0	1	2	0	0	0	0	0	0	0	1	2	0	0	0	0	0	0
Q14061	Cytochrome c oxidase copper chaperone	COX17	1	1	1	1	1	1	0	0	0	0	0	0	1	1	1	0	0	0	0	0	0	1	1	1	0	0	0	0	0	0
Q96IY4	Carboxypeptidase B2	CPB2	1	1	1	1	1	1	0	0	0	0	0	0	1	1	1	0	0	0	0	0	0	1	1	1	0	0	0	0	0	0
Q9Y315	Deoxyribose-phosphate aldolase	DERA	1	7	7	1	1	1	0	0	0	0	0	0	1	1	1	0	0	0	0	0	0	1	1	1	0	0	0	0	0	0
O00273	DNA fragmentation factor subunit alpha	DFFA	1	4	4	1	0	2	0	0	0	0	0	0	1	0	2	0	0	0	0	0	0	1	0	2	0	0	0	0	0	0
P55010	Eukaryotic translation initiation factor 5	EIF5	1	7	7	1	1	1	0	0	0	0	0	0	1	1	1	0	0	0	0	0	0	1	1	1	0	0	0	0	0	0
Q12929	Epidermal growth factor receptor kinase substrate 8	EPS8	1	5	5	1	2	0	0	0	0	0	0	0	1	2	0	0	0	0	0	0	0	1	2	0	0	0	0	0	0	0
P49789	Bis(5-adenosyl)-triphosphatase	FHIT	1	1	1	1	1	1	0	0	0	0	0	0	1	1	1	0	0	0	0	0	0	1	1	1	0	0	0	0	0	0
Q14192	Four and a half LIM domains protein 2	FHL2	1	3	3	2	1	0	0	0	0	0	0	0	2	1	0	0	0	0	0	0	0	2	1	0	0	0	0	0	0	0
P68106	Peptidyl-prolyl cis-trans isomerase FKBP1B	FKBP1B	1	1	1	1	1	1	0	0	0	0	0	0	1	1	1	0	0	0	0	0	0	1	1	1	0	0	0	0	0	0
P26885	Peptidyl-prolyl cis-trans isomerase FKBP2	FKBP2	1	3	3	1	1	1	0	0	0	0	0	0	1	1	1	0	0	0	0	0	0	1	1	1	0	0	0	0	0	0
Q96EK6	Glucosamine 6-phosphate N-acetyltransferase	GNPNAT1	1	3	3	1	1	1	0	0	0	0	0	0	1	1	1	0	0	0	0	0	0	1	1	1	0	0	0	0	0	0
Q9BX68	Histidine triad nucleotide-binding protein 2, mitochondrial	HINT2	1	4	4	1	1	1	0	0	0	0	0	0	1	1	1	0	0	0	0	0	0	1	1	1	0	0	0	0	0	0
Q5SSJ5	Heterochromatin protein 1-binding protein 3	HP1BP3	1	1	1	1	1	1	0	0	0	0	0	0	1	1	1	0	0	0	0	0	0	1	1	1	0	0	0	0	0	0
Q9GZP8	Immortalization up-regulated protein	IMUP	1	1	1	1	1	1	0	0	0	0	0	0	1	1	1	0	0	0	0	0	0	1	1	1	0	0	0	0	0	0
Q9UHB6	LIM domain and actin-binding protein 1	LIMA1	1	9	9	1	1	1	0	0	0	0	0	0	1	1	1	0	0	0	0	0	0	1	1	1	0	0	0	0	0	0
Q6UXB3	Ly6/PLAUR domain-containing protein 2	LYPD2	1	1	1	1	1	1	0	0	0	0	0	0	1	1	1	0	0	0	0	0	0	1	1	1	0	0	0	0	0	0
Q9NTJ4	Alpha-mannosidase 2C1	MAN2C1	1	5	5	1	1	1	0	0	0	0	0	0	1	1	1	0	0	0	0	0	0	1	1	1	0	0	0	0	0	0
Q8IVT2	Mitotic interactor and substrate of PLK1	MISP	1	1	1	1	1	1	0	0	0	0	0	0	1	1	1	0	0	0	0	0	0	1	1	1	0	0	0	0	0	0
Q6IA69	Glutamine-dependent NAD(+) synthetase	NADSYN1	1	3	3	4	1	1	0	0	0	0	0	0	1	1	1	0	0	0	0	0	0	1	1	1	0	0	0	0	0	0
P36873	Serine/threonine-protein phosphatase PP1-gamma catalytic subunit	PPP1CC	1	9	1	4	5	5	1	2	1	2	1	2	1	1	1	0	0	0	0	0	0	1	1	1	0	0	0	0	0	0
Q96C90	Protein phosphatase 1 regulatory subunit 14B	PPP1R14B	1	2	2	4	1	1	0	0	0	0	0	0	1	1	1	0	0	0	0	0	0	1	1	1	0	0	0	0	0	0
Q99436	Proteasome subunit beta type-7	PSMB7	1	4	4	4	0	1	0	0	0	0	0	0	2	0	1	0	0	0	0	0	0	2	0	1	0	0	0	0	0	0
Q96BW5	Phosphotriesterase-related protein	PTER	1	3	3	4	2	0	0	0	0	0	0	0	1	2	0	0	0	0	0	0	0	1	2	0	0	0	0	0	0	0
P62834	Ras-related protein Rap-1A	RAP1A	1	9	1	4	1	1	0	0	0	3	2	2	1	1	1	0	0	0	0	0	0	1	1	1	0	0	0	0	0	0
P98179	RNA-binding protein 3	RBM3	1	3	3	4	1	1	0	0	0	0	0	0	1	1	1	0	0	0	0	0	0	1	1	1	0	0	0	0	0	0
Q9HD89	Resistin	RETN	1	2	2	4	1	1	0	0	0	0	0	0	1	1	1	0	0	0	0	0	0	1	1	1	0	0	0	0	0	0
P35244	Replication protein A 14 kDa subunit	RPA3	1	2	2	4	1	1	0	0	0	0	0	0	1	1	1	0	0	0	0	0	0	1	1	1	0	0	0	0	0	0
P49207	60S ribosomal protein L34	RPL34	1	1	1	4	1	1	0	0	0	0	0	0	1	1	1	0	0	0	0	0	0	1	1	1	0	0	0	0	0	0
O00442	RNA 3-terminal phosphate cyclase	RTCA	1	1	1	4	1	1	0	0	0	0	0	0	1	1	1	0	0	0	0	0	0	1	1	1	0	0	0	0	0	0
P18827	Syndecan-1	SDC1	1	2	2	4	1	1	0	0	0	0	0	0	1	1	1	0	0	0	0	0	0	1	1	1	0	0	0	0	0	0
Q9UJC5	SH3 domain-binding glutamic acid-rich-like protein 2	SH3BGRL2	1	4	3	4	1	1	0	0	0	0	0	0	1	1	1	0	0	0	0	0	0	1	1	1	0	0	0	0	0	0
Q96B97	SH3 domain-containing kinase-binding protein 1	SH3KBP1	1	2	2	4	1	1	0	0	0	0	0	0	1	1	1	0	0	0	0	0	0	1	1	1	0	0	0	0	0	0
Q9NXA8	NAD-dependent protein deacylase sirtuin-5, mitochondrial	SIRT5	1	1	1	4	1	1	0	0	0	0	0	0	1	1	1	0	0	0	0	0	0	1	1	1	0	0	0	0	0	0
O95436	Sodium-dependent phosphate transport protein 2B	SLC34A2	1	7	7	4	0	1	0	0	0	0	0	0	2	0	1	0	0	0	0	0	0	2	0	1	0	0	0	0	0	0
P04179	Superoxide dismutase [Mn], mitochondrial	SOD2	1	3	3	4	1	1	0	0	0	0	0	0	1	1	1	0	0	0	0	0	0	1	1	1	0	0	0	0	0	0
P49458	Signal recognition particle 9 kDa protein	SRP9	1	3	3	4	1	1	0	0	0	0	0	0	1	1	1	0	0	0	0	0	0	1	1	1	0	0	0	0	0	0
Q96I99	Succinyl-CoA ligase [GDP-forming] subunit beta, mitochondrial	SUCLG2	1	4	4	4	1	1	0	0	0	0	0	0	1	1	1	0	0	0	0	0	0	1	1	1	0	0	0	0	0	0
Q12962	Transcription initiation factor TFIID subunit 10	TAF10	1	1	1	4	1	1	0	0	0	0	0	0	1	1	1	0	0	0	0	0	0	1	1	1	0	0	0	0	0	0
P26639	Threonine--tRNA ligase, cytoplasmic	TARS	1	6	6	4	1	1	0	0	0	0	0	0	1	1	1	0	0	0	0	0	0	1	1	1	0	0	0	0	0	0

P53597	Succinyl-CoA ligase [ADP/GDP-forming] subunit alpha, mitochondrial	SUCLG1	1	4	4	4	1	0	0	0	0	0	0	0	0	1	0	0	0	0	0	0	0	0	1	0	0	0	0	0	0	0
Q8WY91	THAP domain-containing protein 4	THAP4	1	2	2	4	0	0	0	0	0	0	0	0	1	0	0	0	0	0	0	0	0	1	0	0	0	0	0	0	0	0
Q15642	Cdc42-interacting protein 4	TRIP10	1	6	6	4	0	1	0	0	0	0	0	0	0	0	1	0	0	0	0	0	0	0	0	1	0	0	0	0	0	0
Q9H0U9	Testis-specific Y-encoded-like protein 1	TSPYL1	1	1	1	4	1	0	0	0	0	0	0	0	0	1	0	0	0	0	0	0	0	0	1	0	0	0	0	0	0	0
Q5R3I4	Tetratricopeptide repeat protein 38	TTC38	1	3	3	4	1	0	0	0	0	0	0	0	0	1	0	0	0	0	0	0	0	0	1	0	0	0	0	0	0	0
Q9Y4E8	Ubiquitin carboxyl-terminal hydrolase 15	USP15	1	3	2	4	1	0	0	0	0	0	0	0	0	1	0	0	0	0	0	0	0	0	1	0	0	0	0	0	0	0
Q15836	Vesicle-associated membrane protein 3	VAMP3	1	2	1	4	0	1	0	0	0	0	0	0	0	0	1	0	0	0	0	0	0	0	0	1	0	0	0	0	0	0
O00534	von Willebrand factor A domain-containing protein 5A	VWA5A	1	8	8	4	0	0	0	0	0	0	0	0	1	0	0	0	0	0	0	0	0	1	0	0	0	0	0	0	0	0
P17174	Aspartate aminotransferase, cytoplasmic	GOT1	1	6	6	4	4	3	0	0	1	0	0	0	4	4	3	0	0	1	0	0	0	4	4	3	0	0	1	0	0	0
P62873	Guanine nucleotide-binding protein G(I)/G(S)/G(T) subunit beta-1	GNB1	2	12	7	3	3	2	1	0	0	0	0	0	1	1	1	1	0	0	0	0	0	3	3	2	1	0	0	0	0	0
Q07020	60S ribosomal protein L18	RPL18	1	5	5	4	3	3	0	0	1	0	0	0	2	3	3	0	0	1	0	0	0	2	3	3	0	0	1	0	0	0
O95359	Transforming acidic coiled-coil-containing protein 2	TACC2	1	5	5	4	3	2	1	0	0	0	0	0	2	3	2	1	0	0	0	0	0	2	3	2	1	0	0	0	0	0
P30046	D-dopachrome decarboxylase	DDT;DDTL	2	5	5	3	2	1	0	1	0	0	0	0	3	2	1	0	1	0	0	0	0	3	2	1	0	1	0	0	0	0
P26373	60S ribosomal protein L13	RPL13	1	4	4	4	2	2	1	0	0	0	0	0	2	2	2	1	0	0	0	0	0	2	2	2	1	0	0	0	0	0
P18621	60S ribosomal protein L17	RPL17	1	6	6	4	1	2	0	1	0	0	0	0	3	1	2	0	1	0	0	0	0	3	1	2	0	1	0	0	0	0
P46781	40S ribosomal protein S9	RPS9	1	9	9	4	2	2	0	1	0	0	0	0	2	2	2	0	1	0	0	0	0	2	2	2	0	1	0	0	0	0
P30626	Sorcin	SRI	1	5	5	4	1	1	0	1	0	0	0	0	2	1	1	0	1	0	0	0	0	2	1	1	0	1	0	0	0	0
P62701	40S ribosomal protein S4, X isoform	RPS4X	3	5	5	1	1	1	1	0	0	0	0	0	1	1	1	1	0	0	0	0	0	1	1	1	1	0	0	0	0	0
Q9BY44	Eukaryotic translation initiation factor 2A	EIF2A	1	3	3	2	0	1	0	1	0	0	0	0	2	0	1	0	1	0	0	0	0	2	0	1	0	1	0	0	0	0
O75822	Eukaryotic translation initiation factor 3 subunit J	EIF3J	1	2	2	1	1	1	0	0	1	0	0	0	1	1	1	0	0	1	0	0	0	1	1	1	0	0	1	0	0	0
Q8NDC0	MAPK-interacting and spindle-stabilizing protein-like	MAPK1IP1L	1	1	1	1	1	1	0	0	1	0	0	0	1	1	1	0	0	1	0	0	0	1	1	1	0	0	1	0	0	0
Q9GZP4	PITH domain-containing protein 1	PITHD1	1	1	1	4	1	1	1	0	0	0	0	0	1	1	1	1	0	0	0	0	0	1	1	1	1	0	0	0	0	0
P53999	Activated RNA polymerase II transcriptional coactivator p15	SUB1	1	1	1	4	1	1	0	1	0	0	0	0	1	1	1	0	1	0	0	0	0	1	1	1	0	1	0	0	0	0
Q9BRP8	Partner of Y14 and mago	WIBG	1	2	2	4	1	0	0	0	1	0	0	0	2	1	0	0	0	1	0	0	0	2	1	0	0	0	1	0	0	0
P61163	Alpha-centractin	ACTR1A	1	13	8	0	0	1	0	1	0	0	0	0	0	0	1	0	0	0	0	0	0	0	0	1	0	1	0	0	0	0
O14672	Disintegrin and metalloproteinase domain-containing protein 10	ADAM10	1	7	7	0	1	0	0	1	0	0	0	0	0	1	0	0	1	0	0	0	0	0	1	0	0	1	0	0	0	0
P01031	Complement C5	C5	1	13	13	0	0	1	1	0	0	0	0	0	0	0	1	1	0	0	0	0	0	0	0	1	1	0	0	0	0	0
P36980	Complement factor H-related protein 2	CFHR2	1	4	2	0	0	1	0	0	1	1	0	0	0	0	1	0	0	1	0	0	0	0	0	1	0	0	1	0	0	0
O60869	Endothelial differentiation-related factor 1	EDF1	1	2	2	0	1	0	0	0	1	0	0	0	0	1	0	0	0	1	0	0	0	0	1	0	0	0	1	0	0	0
P60510	Serine/threonine-protein phosphatase 4 catalytic subunit	PPP4C	1	3	2	4	2	1	0	0	1	0	0	0	0	1	0	0	0	1	0	0	0	0	1	0	0	0	1	0	0	0
Q9NQG5	Regulation of nuclear pre-mRNA domain-containing protein 1B	RPRD1B	1	4	4	4	1	0	0	0	1	0	0	0	0	1	0	0	0	1	0	0	0	0	1	0	0	0	1	0	0	0
P04350	Tubulin beta-4A chain	TUBB4A	4	20	0	5	4	5	7	7	9	10	8	12	0	0	0	0	0	0	0	0	0	0	0	0	0	0	1	0	0	0
P24752	Acetyl-CoA acetyltransferase, mitochondrial	ACAT1	1	5	5	0	0	0	0	1	0	0	0	0	0	0	0	0	1	0	0	0	0	0	0	0	0	1	0	0	0	0
O75342	Arachidonate 12-lipoxygenase, 12R-type	ALOX12B	1	1	1	0	0	0	1	0	0	0	0	0	0	0	0	1	0	0	0	0	0	0	0	0	1	0	0	0	0	0
Q86V81	THO complex subunit 4	ALYREF	1	1	1	0	0	0	0	1	0	0	0	0	0	0	0	0	1	0	0	0	0	0	0	0	0	1	0	0	0	0
Q6DD88	Atlastin-3	ATL3	1	2	2	0	0	0	1	0	0	0	0	0	0	0	0	1	0	0	0	0	0	0	0	0	1	0	0	0	0	0
P54707	Potassium-transporting ATPase alpha chain 2	ATP12A	1	3	1	0	1	0	0	0	1	0	0	0	0	0	0	0	0	1	0	0	0	0	0	0	0	0	1	0	0	0
P57730	Caspase recruitment domain-containing protein 18	CARD18	1	1	1	0	0	0	0	0	1	0	0	0	0	0	0	0	0	1	0	0	0	0	0	0	0	0	1	0	0	0
Q9UJ71	C-type lectin domain family 4 member K	CD207	1	1	1	0	0	0	0	0	1	0	0	0	0	0	0	0	0	1	0	0	0	0	0	0	0	0	1	0	0	0
P04233	HLA class II histocompatibility antigen gamma chain	CD74	1	1	1	0	0	0	0	0	1	0	0	0	0	0	0	0	0	1	0	0	0	0	0	0	0	0	1	0	0	0
P53618	Coatomer subunit beta	COPB1	1	8	8	0	0	0	1	0	0	0	0	0	0	0	0	1	0	0	0	0	0	0	0	0	1	0	0	0	0	0
P55060	Exportin-2	CSE1L	1	3	3	0	0	0	1	0	0	0	0	0	0	0	0	1	0	0	0	0	0	0	0	0	1	0	0	0	0	0
O60716	Catenin delta-1	CTNND1	1	8	8	0	0	0	0	0	1	0	0	0	0	0	0	0	0	1	0	0	0	0	0	0	0	0	1	0	0	0
O60911	Cathepsin L2	CTSV	1	2	2	0	0	0	0	0	1	0	0	0	0	0	0	0	0	1	0	0	0	0	0	0	0	0	1	0	0	0
Q13618	Cullin-3	CUL3	1	4	4	0	0	0	1	0	0	0	0	0	0	0	0	1	0	0	0	0	0	0	0	0	1	0	0	0	0	0
P17844	Probable ATP-dependent RNA helicase DDX5	DDX5	1	1	1	0	0	0	0	1	0	0	0	0	0	0	0	0	1	0	0	0	0	0	0	0	0	1	0	0	0	0
Q96LJ7	Dehydrogenase/reductase SDR family member 1	DHRS1	1	4	4	0	0	0	0	1	0	0	0	0	0	0	0	0	1	0	0	0	0	0	0	0	0	1	0	0	0	0
O60610	Protein diaphanous homolog 1	DIAPH1	1	9	9	0	0	0	1	0	0	0	0	0	0	0	0	1	0	0	0	0	0	0	0	0	1	0	0	0	0	0
Q99615	DnaJ homolog subfamily C member 7	DNAJC7	1	1	1	0	0	0	1	0	0	0	0	0	0	0	0	1	0	0	0	0	0	0	0	0	1	0	0	0	0	0
P60228	Eukaryotic translation initiation factor 3 subunit E	EIF3E	1	3	3	0	0	0	1	0	0	0	0	0	0	0	0	1	0	0	0	0	0	0	0	0	1	0	0	0	0	0

Q6P1N9	Putative deoxyribonuclease TATDN1	TATDN1	1	1	1	4	1	1	0	0	0	0	0	0	1	1	1	0	0	0	0	0	0	1	1	1	0	0	0	0	0	0
Q99426	Tubulin-folding cofactor B	TBCB	1	2	2	4	1	1	0	0	0	0	0	0	1	1	1	0	0	0	0	0	0	1	1	1	0	0	0	0	0	0
O60635	Tetraspanin-1	TSPAN1	1	1	1	4	1	1	0	0	0	0	0	0	1	1	1	0	0	0	0	0	0	1	1	1	0	0	0	0	0	0
Q9BV40	Vesicle-associated membrane protein 8	VAMP8	1	3	3	4	2	1	0	0	0	0	0	0	0	2	1	0	0	0	0	0	0	0	2	1	0	0	0	0	0	0
Q9H9H4	Vacuolar protein sorting-associated protein 37B	VPS37B	1	2	2	4	1	1	0	0	0	0	0	0	1	1	1	0	0	0	0	0	0	1	1	1	0	0	0	0	0	0
Q86YZ3	Hornerin	HRNR	2	1	1	1	1	0	0	0	0	0	0	0	1	1	0	0	0	0	0	0	0	1	1	0	0	0	0	0	0	0
P42330	Aldo-keto reductase family 1 member C3	AKR1C3	2	9	2	7	6	6	1	1	1	1	2	2	2	0	0	0	0	0	0	0	0	2	0	0	0	0	0	0	0	0
P47989	Xanthine dehydrogenase/oxidase	XDH	2	15	15	1	0	1	0	0	0	0	0	0	1	0	1	0	0	0	0	0	0	1	0	1	0	0	0	0	0	0
P47813	Eukaryotic translation initiation factor 1A, X-chromosomal	EIF1AX/Y	2	1	1	1	1	0	0	0	0	0	0	0	1	1	0	0	0	0	0	0	0	1	1	0	0	0	0	0	0	0
Q6P996	Pyridoxal-dependent decarboxylase domain-containing protein 1	PDXDC1	2	3	3	1	0	1	0	0	0	0	0	0	1	0	1	0	0	0	0	0	0	1	0	1	0	0	0	0	0	0
Q8IVF2	Protein AHNAK2	AHNAK2	1	2	2	0	2	0	0	0	0	0	0	0	0	2	0	0	0	0	0	0	0	0	2	0	0	0	0	0	0	0
Q9BTT0	Acidic leucine-rich nuclear phosphoprotein 32 family member E	ANP32E	1	2	2	1	1	0	0	0	0	0	0	0	1	1	0	0	0	0	0	0	0	1	1	0	0	0	0	0	0	0
Q9HB07	UPF0160 protein MYG1, mitochondrial	C12orf10	1	1	1	0	1	1	0	0	0	0	0	0	0	1	1	0	0	0	0	0	0	0	1	1	0	0	0	0	0	0
P46109	Crk-like protein	CRKL	1	3	3	1	0	1	0	0	0	0	0	0	1	0	1	0	0	0	0	0	0	1	0	1	0	0	0	0	0	0
P00387	NADH-cytochrome b5 reductase 3	CYB5R3	1	6	6	1	0	1	0	0	0	0	0	0	1	0	1	0	0	0	0	0	0	1	0	1	0	0	0	0	0	0
Q5TDH0	Protein DDI1 homolog 2	DDI2	1	4	4	2	0	0	0	0	0	0	0	0	2	0	0	0	0	0	0	0	0	2	0	0	0	0	0	0	0	0
O43583	Density-regulated protein	DENR	1	2	2	1	0	1	0	0	0	0	0	0	1	0	1	0	0	0	0	0	0	1	0	1	0	0	0	0	0	0
Q9ULA0	Aspartyl aminopeptidase	DNPEP	1	3	3	0	0	2	0	0	0	0	0	0	0	0	2	0	0	0	0	0	0	0	0	2	0	0	0	0	0	0
O43598	2-deoxynucleoside 5-phosphate N-hydrolase 1	DNPH1	1	3	3	0	1	1	0	0	0	0	0	0	0	1	1	0	0	0	0	0	0	0	1	1	0	0	0	0	0	0
P23588	Eukaryotic translation initiation factor 4B	EIF4B	1	3	3	1	1	0	0	0	0	0	0	0	1	1	0	0	0	0	0	0	0	1	1	0	0	0	0	0	0	0
O43768	Alpha-endosulfine	ENSA	1	1	1	0	1	1	0	0	0	0	0	0	0	1	1	0	0	0	0	0	0	0	1	1	0	0	0	0	0	0
P48506	Glutamate--cysteine ligase catalytic subunit	GCLC	1	3	3	1	1	0	0	0	0	0	0	0	1	1	0	0	0	0	0	0	0	1	1	0	0	0	0	0	0	0
Q9Y2T3	Guanine deaminase	GDA	1	2	2	1	1	0	0	0	0	0	0	0	1	1	0	0	0	0	0	0	0	1	1	0	0	0	0	0	0	0
P14317	Hematopoietic lineage cell-specific protein	HCLS1	1	1	1	1	0	1	0	0	0	0	0	0	1	0	1	0	0	0	0	0	0	1	0	1	0	0	0	0	0	0
Q08623	Pseudouridine-5-phosphatase	HDHD1	1	2	2	1	0	1	0	0	0	0	0	0	1	0	1	0	0	0	0	0	0	1	0	1	0	0	0	0	0	0
Q9NRV9	Heme-binding protein 1	HEBP1	1	4	4	0	1	1	0	0	0	0	0	0	0	1	1	0	0	0	0	0	0	0	1	1	0	0	0	0	0	0
P00492	Hypoxanthine-guanine phosphoribosyltransferase	HPRT1	1	4	4	1	1	0	0	0	0	0	0	0	1	1	0	0	0	0	0	0	0	1	1	0	0	0	0	0	0	0
Q96CN7	Isochorismatase domain-containing protein 1	ISOC1	1	4	4	1	1	0	0	0	0	0	0	0	1	1	0	0	0	0	0	0	0	1	1	0	0	0	0	0	0	0
Q06136	3-ketodihydrosphingosine reductase	KDSR	1	3	3	0	1	1	0	0	0	0	0	0	0	1	1	0	0	0	0	0	0	0	1	1	0	0	0	0	0	0
Q53QV2	Protein LBH	LBH	1	1	1	1	0	1	0	0	0	0	0	0	1	0	1	0	0	0	0	0	0	1	0	1	0	0	0	0	0	0
Q9NUP9	Protein lin-7 homolog C	LIN7C	1	2	2	1	1	0	0	0	0	0	0	0	1	1	0	0	0	0	0	0	0	1	1	0	0	0	0	0	0	0
Q9Y333	U6 snRNA-associated Sm-like protein LSm2	LSM2	1	2	2	1	0	1	0	0	0	0	0	0	1	0	1	0	0	0	0	0	0	1	0	1	0	0	0	0	0	0
Q14696	LDLR chaperone MESD	MESDC2	1	1	1	0	1	1	0	0	0	0	0	0	0	1	1	0	0	0	0	0	0	0	1	1	0	0	0	0	0	0
P02795	Metallothionein-2	MT2A	1	1	1	1	0	1	0	0	0	0	0	0	1	0	1	0	0	0	0	0	0	1	0	1	0	0	0	0	0	0
Q99747	Gamma-soluble NSF attachment protein	NAPG	1	2	2	4	2	0	0	0	0	0	0	0	0	2	0	0	0	0	0	0	0	0	2	0	0	0	0	0	0	0
Q8WVJ2	NudC domain-containing protein 2	NUDCD2	1	3	3	4	1	0	0	0	0	0	0	0	1	1	0	0	0	0	0	0	0	1	1	0	0	0	0	0	0	0
Q9UKS6	Protein kinase C and casein kinase substrate in neurons protein 3	PACSIN3	1	3	3	4	0	1	0	0	0	0	0	0	1	0	1	0	0	0	0	0	0	1	0	1	0	0	0	0	0	0
Q96IZ0	PRKC apoptosis WT1 regulator protein	PAWR	1	1	1	4	1	1	0	0	0	0	0	0	0	1	1	0	0	0	0	0	0	0	1	1	0	0	0	0	0	0
Q06203	Amidophosphoribosyltransferase	PPAT	1	1	1	4	1	1	0	0	0	0	0	0	0	1	1	0	0	0	0	0	0	0	1	1	0	0	0	0	0	0
Q8TAE6	Protein phosphatase 1 regulatory subunit 14C	PPP1R14C	1	1	1	4	1	1	0	0	0	0	0	0	0	1	1	0	0	0	0	0	0	0	1	1	0	0	0	0	0	0
Q99873	Protein arginine N-methyltransferase 1	PRMT1	1	2	2	4	0	1	0	0	0	0	0	0	1	0	1	0	0	0	0	0	0	1	0	1	0	0	0	0	0	0
O00233	26S proteasome non-ATPase regulatory subunit 9	PSMD9	1	2	2	4	0	1	0	0	0	0	0	0	1	0	1	0	0	0	0	0	0	1	0	1	0	0	0	0	0	0
Q92530	Proteasome inhibitor PI31 subunit	PSMF1	1	3	3	4	1	1	0	0	0	0	0	0	0	1	1	0	0	0	0	0	0	0	1	1	0	0	0	0	0	0
Q96NY8	Nectin-4	PVRL4	1	3	3	4	0	1	0	0	0	0	0	0	1	0	1	0	0	0	0	0	0	1	0	1	0	0	0	0	0	0
O75884	Putative hydrolase RBBP9	RBBP9	1	2	2	4	1	0	0	0	0	0	0	0	1	1	0	0	0	0	0	0	0	1	1	0	0	0	0	0	0	0
Q9UBF6	RING-box protein 2	RNF7	1	1	1	4	0	1	0	0	0	0	0	0	1	0	1	0	0	0	0	0	0	1	0	1	0	0	0	0	0	0
P49247	Ribose-5-phosphate isomerase	RPIA	1	1	1	4	1	1	0	0	0	0	0	0	0	1	1	0	0	0	0	0	0	0	1	1	0	0	0	0	0	0
P42766	60S ribosomal protein L35	RPL35	1	3	3	4	0	1	0	0	0	0	0	0	1	0	1	0	0	0	0	0	0	1	0	1	0	0	0	0	0	0
Q96RF0	Sorting nexin-18	SNX18	1	1	1	4	0	1	0	0	0	0	0	0	1	0	1	0	0	0	0	0	0	1	0	1	0	0	0	0	0	0
P09758	Tumor-associated calcium signal transducer 2	TACSTD2	1	11	11	4	1	0	0	0	0	0	0	0	1	1	0	0	0	0	0	0	0	1	1	0	0	0	0	0	0	0

P29144	Tripeptidyl-peptidase 2	TPP2	1	8	8	4	0	1	0	0	0	0	0	0	1	0	1	0	0	0	0	0	0	1	0	1	0	0	0	0	0	0
O94811	Tubulin polymerization-promoting protein	TPPP	1	3	3	4	1	1	0	0	0	0	0	0	0	1	1	0	0	0	0	0	0	0	1	1	0	0	0	0	0	0
Q04323	UBX domain-containing protein 1	UBXN1	1	1	1	4	1	0	0	0	0	0	0	0	1	1	0	0	0	0	0	0	0	1	1	0	0	0	0	0	0	0
P09488	Glutathione S-transferase Mu 1	GSTM1/M4	3	4	3	0	1	0	0	0	0	0	0	0	0	0	0	0	0	0	0	0	0	0	1	0	0	0	0	0	0	0
Q6DKI2	Galectin-9C	LGALS9C/9B	2	4	2	0	1	0	0	0	0	0	0	0	0	1	0	0	0	0	0	0	0	0	1	0	0	0	0	0	0	0
E5RIL1	Uroplakin-3b-like protein	UPK3BL	2	3	3	1	0	0	0	0	0	0	0	0	1	0	0	0	0	0	0	0	0	1	0	0	0	0	0	0	0	0
Q9BWD1	Acetyl-CoA acetyltransferase, cytosolic	ACAT2	1	7	7	0	0	1	0	0	0	0	0	0	0	0	1	0	0	0	0	0	0	0	0	1	0	0	0	0	0	0
P51648	Fatty aldehyde dehydrogenase	ALDH3A2	1	8	7	1	1	1	0	0	0	0	1	0	1	0	0	0	0	0	0	0	0	1	0	0	0	0	0	0	0	0
Q01432	AMP deaminase 3	AMPD3	1	4	4	1	0	0	0	0	0	0	0	0	1	0	0	0	0	0	0	0	0	1	0	0	0	0	0	0	0	0
P15144	Aminopeptidase N	ANPEP	1	7	7	1	0	0	0	0	0	0	0	0	1	0	0	0	0	0	0	0	0	1	0	0	0	0	0	0	0	0
P13671	Complement component C6	C6	1	5	5	1	0	0	0	0	0	0	0	0	1	0	0	0	0	0	0	0	0	1	0	0	0	0	0	0	0	0
Q14444	Caprin-1	CAPRIN1	1	1	1	0	1	0	0	0	0	0	0	0	0	1	0	0	0	0	0	0	0	0	1	0	0	0	0	0	0	0
O14618	Copper chaperone for superoxide dismutase	CCS	1	2	2	1	0	0	0	0	0	0	0	0	1	0	0	0	0	0	0	0	0	1	0	0	0	0	0	0	0	0
Q99828	Calcium and integrin-binding protein 1	CIB1	1	4	4	0	1	0	0	0	0	0	0	0	0	1	0	0	0	0	0	0	0	0	1	0	0	0	0	0	0	0
Q14011	Cold-inducible RNA-binding protein	CIRBP	1	6	6	0	0	1	0	0	0	0	0	0	0	0	1	0	0	0	0	0	0	0	0	1	0	0	0	0	0	0
P54105	Methylosome subunit pICln	CLNS1A	1	2	2	0	0	1	0	0	0	0	0	0	0	0	1	0	0	0	0	0	0	0	0	1	0	0	0	0	0	0
O75390	Citrate synthase, mitochondrial	CS	1	2	2	0	0	1	0	0	0	0	0	0	0	0	1	0	0	0	0	0	0	0	0	1	0	0	0	0	0	0
Q6UWP2	Dehydrogenase/reductase SDR family member 11	DHRS11	1	2	2	0	1	0	0	0	0	0	0	0	0	1	0	0	0	0	0	0	0	0	1	0	0	0	0	0	0	0
Q9UBS4	DnaJ homolog subfamily B member 11	DNAJB11	1	2	2	0	0	1	0	0	0	0	0	0	0	0	1	0	0	0	0	0	0	0	0	1	0	0	0	0	0	0
Q14195	Dihydropyrimidinase-related protein 3	DPYSL3	1	2	2	0	1	0	0	0	0	0	0	0	0	1	0	0	0	0	0	0	0	0	1	0	0	0	0	0	0	0
Q8TEA8	D-tyrosyl-tRNA(Tyr) deacylase 1	DTD1	1	1	1	0	0	1	0	0	0	0	0	0	0	0	1	0	0	0	0	0	0	0	0	1	0	0	0	0	0	0
Q13011	Delta(3,5)-Delta(2,4)-dienoyl-CoA isomerase, mitochondrial	ECH1	1	7	7	1	0	0	0	0	0	0	0	0	1	0	0	0	0	0	0	0	0	1	0	0	0	0	0	0	0	0
Q9H4G0	Band 4.1-like protein 1	EPB41L1	1	9	9	1	0	0	0	0	0	0	0	0	1	0	0	0	0	0	0	0	0	1	0	0	0	0	0	0	0	0
Q9Y2J2	Band 4.1-like protein 3	EPB41L3	1	2	2	0	0	1	0	0	0	0	0	0	0	0	1	0	0	0	0	0	0	0	0	1	0	0	0	0	0	0
Q9HA64	Ketosamine-3-kinase	FN3KRP	1	1	1	0	1	0	0	0	0	0	0	0	0	1	0	0	0	0	0	0	0	0	1	0	0	0	0	0	0	0
O14908	PDZ domain-containing protein GIPC1	GIPC1	1	2	2	1	0	0	0	0	0	0	0	0	1	0	0	0	0	0	0	0	0	1	0	0	0	0	0	0	0	0
P60983	Glia maturation factor beta	GMFB	1	2	2	1	0	0	0	0	0	0	0	0	1	0	0	0	0	0	0	0	0	1	0	0	0	0	0	0	0	0
Q8TDQ7	Glucosamine-6-phosphate isomerase 2	GNPDA2	1	7	2	5	6	5	0	0	0	1	1	1	0	1	0	0	0	0	0	0	0	0	1	0	0	0	0	0	0	0
Q9NQX3	Gephyrin	GPHN	1	4	4	1	0	0	0	0	0	0	0	0	1	0	0	0	0	0	0	0	0	1	0	0	0	0	0	0	0	0
Q16774	Guanylate kinase	GUK1	1	2	2	1	0	0	0	0	0	0	0	0	1	0	0	0	0	0	0	0	0	1	0	0	0	0	0	0	0	0
Q9H0R4	Haloacid dehalogenase-like hydrolase domain-containing protein 2	HDHD2	1	3	3	0	0	1	0	0	0	0	0	0	0	0	1	0	0	0	0	0	0	0	0	1	0	0	0	0	0	0
P31942	Heterogeneous nuclear ribonucleoprotein H3	HNRNPH3	1	2	2	0	1	0	0	0	0	0	0	0	0	1	0	0	0	0	0	0	0	0	1	0	0	0	0	0	0	0
Q9Y547	Intraflagellar transport protein 25 homolog	HSPB11	1	1	1	0	0	1	0	0	0	0	0	0	0	0	1	0	0	0	0	0	0	0	0	1	0	0	0	0	0	0
Q7Z6Z7	E3 ubiquitin-protein ligase HUWE1	HUWE1	1	6	6	0	0	1	0	0	0	0	0	0	0	0	1	0	0	0	0	0	0	0	0	1	0	0	0	0	0	0
P05362	Intercellular adhesion molecule 1	ICAM1	1	2	2	0	1	0	0	0	0	0	0	0	0	1	0	0	0	0	0	0	0	0	1	0	0	0	0	0	0	0
P14780	Matrix metalloproteinase-9	MMP9	1	5	5	1	0	0	0	0	0	0	0	0	1	0	0	0	0	0	0	0	0	1	0	0	0	0	0	0	0	0
P15531	Nucleoside diphosphate kinase A	NME1	1	6	2	4	3	3	2	3	2	3	2	2	1	0	0	0	0	0	0	0	0	1	0	0	0	0	0	0	0	0
Q5TFE4	5-nucleotidase domain-containing protein 1	NT5DC1	1	4	4	4	0	0	0	0	0	0	0	0	1	0	0	0	0	0	0	0	0	1	0	0	0	0	0	0	0	0
P21589	5-nucleotidase	NT5E	1	4	4	4	0	1	0	0	0	0	0	0	0	0	1	0	0	0	0	0	0	0	0	1	0	0	0	0	0	0
Q8WW12	PEST proteolytic signal-containing nuclear protein	PCNP	1	1	1	4	0	0	0	0	0	0	0	0	1	0	0	0	0	0	0	0	0	1	0	0	0	0	0	0	0	0
P62136	Serine/threonine-protein phosphatase PP1-alpha catalytic subunit	PPP1CA	1	11	3	4	4	4	1	2	1	2	1	2	1	0	0	0	0	0	0	0	0	1	0	0	0	0	0	0	0	0
Q5H9R7	Serine/threonine-protein phosphatase 6 regulatory subunit 3	PPP6R3	1	1	1	4	0	1	0	0	0	0	0	0	0	0	1	0	0	0	0	0	0	0	0	1	0	0	0	0	0	0
P62266	40S ribosomal protein S23	RPS23	1	4	4	4	1	0	0	0	0	0	0	0	0	1	0	0	0	0	0	0	0	0	1	0	0	0	0	0	0	0
O94979	Protein transport protein Sec31A	SEC31A	1	9	9	4	0	0	0	0	0	0	0	0	1	0	0	0	0	0	0	0	0	1	0	0	0	0	0	0	0	0
P08185	Corticosteroid-binding globulin	SERPINA6	1	3	3	4	0	1	0	0	0	0	0	0	0	0	1	0	0	0	0	0	0	0	0	1	0	0	0	0	0	0
Q9NR46	Endophilin-B2	SH3GLB2	1	9	9	4	0	0	0	0	0	0	0	0	1	0	0	0	0	0	0	0	0	1	0	0	0	0	0	0	0	0
O60493	Sorting nexin-3	SNX3	1	2	2	4	0	0	0	0	0	0	0	0	1	0	0	0	0	0	0	0	0	1	0	0	0	0	0	0	0	0
Q9Y5X3	Sorting nexin-5	SNX5	1	7	7	4	0	0	0	0	0	0	0	0	1	0	0	0	0	0	0	0	0	1	0	0	0	0	0	0	0	0
O60271	C-Jun-amino-terminal kinase-interacting protein 4	SPAG9	1	2	2	4	0	0	0	0	0	0	0	0	1	0	0	0	0	0	0	0	0	1	0	0	0	0	0	0	0	0
P27105	Erythrocyte band 7 integral membrane protein	STOM	1	5	5	4	0	1	0	0	0	0	0	0	0	0	1	0	0	0	0	0	0	0	0	1	0	0	0	0	0	0

P06730	Eukaryotic translation initiation factor 4E	EIF4E	1	2	2	0	0	0	0	1	0	0	0	0	0	0	0	0	1	0	0	0	0	0	0	0	0	1	0	0	0	0
Q9NRM1	Enamelin	ENAM	1	1	1	0	0	0	0	1	0	0	0	0	0	0	0	0	1	0	0	0	0	0	0	0	0	1	0	0	0	0
Q52LJ0	Protein FAM98B	FAM98B	1	2	2	0	0	0	0	1	0	0	0	0	0	0	0	0	1	0	0	0	0	0	0	0	0	1	0	0	0	0
Q9Y6R7	IgGFc-binding protein	FCGBP	1	6	6	0	0	0	0	0	1	0	0	0	0	0	0	0	0	1	0	0	0	0	0	0	0	0	1	0	0	0
Q5T1M5	FK506-binding protein 15	FKBP15	1	2	2	0	0	0	0	0	1	0	0	0	0	0	0	0	0	1	0	0	0	0	0	0	0	0	1	0	0	0
Q8N4A0	Polypeptide N-acetylgalactosaminyltransferase 4	GALNT4	1	2	2	0	0	0	0	1	0	0	0	0	0	0	0	0	1	0	0	0	0	0	0	0	0	1	0	0	0	0
P13747	HLA class I histocompatibility antigen, alpha chain E	HLA-E	1	2	1	0	0	0	0	0	1	0	0	0	0	0	0	0	0	1	0	0	0	0	0	0	0	0	1	0	0	0
P30519	Heme oxygenase 2	HMOX2	1	1	1	0	0	0	1	0	0	0	0	0	0	0	0	1	0	0	0	0	0	0	0	0	1	0	0	0	0	0
Q9UHA7	Interleukin-36 alpha	IL36A	1	1	1	0	0	0	1	0	0	0	0	0	0	0	0	1	0	0	0	0	0	0	0	0	1	0	0	0	0	0
Q12905	Interleukin enhancer-binding factor 2	ILF2	1	3	3	0	0	0	0	0	1	0	0	0	0	0	0	0	0	1	0	0	0	0	0	0	0	0	1	0	0	0
P17301	Integrin alpha-2	ITGA2	1	6	6	0	0	0	0	1	0	0	0	0	0	0	0	0	1	0	0	0	0	0	0	0	0	1	0	0	0	0
Q2I0M4	Leucine-rich repeat-containing protein 26	LRRC26	1	1	1	0	0	0	1	0	0	0	0	0	0	0	0	1	0	0	0	0	0	0	0	0	1	0	0	0	0	0
Q96AG4	Leucine-rich repeat-containing protein 59	LRRC59	1	3	3	0	0	0	0	0	1	0	0	0	0	0	0	0	0	1	0	0	0	0	0	0	0	0	1	0	0	0
P52815	39S ribosomal protein L12, mitochondrial	MRPL12	1	2	2	0	0	0	0	1	0	0	0	0	0	0	0	0	1	0	0	0	0	0	0	0	0	1	0	0	0	0
P55769	NHP2-like protein 1	NHP2L1	1	2	2	4	0	0	1	0	0	0	0	0	0	0	0	1	0	0	0	0	0	0	0	0	1	0	0	0	0	0
O75340	Programmed cell death protein 6	PDCD6	1	7	7	4	0	0	0	0	1	0	0	0	0	0	0	0	0	1	0	0	0	0	0	0	0	0	1	0	0	0
O00232	26S proteasome non-ATPase regulatory subunit 12	PSMD12	1	6	6	4	0	0	0	1	0	0	0	0	0	0	0	0	1	0	0	0	0	0	0	0	0	1	0	0	0	0
Q9NP72	Ras-related protein Rab-18	RAB18	1	6	6	4	0	0	1	0	0	0	0	0	0	0	0	1	0	0	0	0	0	0	0	0	1	0	0	0	0	0
P54136	Arginine--tRNA ligase, cytoplasmic	RARS	1	11	11	4	0	0	0	1	0	0	0	0	0	0	0	0	1	0	0	0	0	0	0	0	0	1	0	0	0	0
Q15287	RNA-binding protein with serine-rich domain 1	RNPS1	1	1	1	4	0	0	1	0	0	0	0	0	0	0	0	1	0	0	0	0	0	0	0	0	1	0	0	0	0	0
P08708	40S ribosomal protein S17	RPS17	1	4	4	4	0	0	1	0	0	0	0	0	0	0	0	1	0	0	0	0	0	0	0	0	1	0	0	0	0	0
P62851	40S ribosomal protein S25	RPS25	1	2	2	4	0	0	1	0	0	0	0	0	0	0	0	1	0	0	0	0	0	0	0	0	1	0	0	0	0	0
Q9NQC3	Reticulon-4	RTN4	1	2	2	4	0	0	1	0	0	0	0	0	0	0	0	1	0	0	0	0	0	0	0	0	1	0	0	0	0	0
Q9Y3A5	Ribosome maturation protein SBDS	SBDS	1	5	5	4	0	0	1	0	0	0	0	0	0	0	0	1	0	0	0	0	0	0	0	0	1	0	0	0	0	0
O60613	15 kDa selenoprotein	SELENOF	1	4	4	4	0	0	0	1	0	0	0	0	0	0	0	0	1	0	0	0	0	0	0	0	0	1	0	0	0	0
P09661	U2 small nuclear ribonucleoprotein A	SNRPA1	1	2	2	4	0	0	0	1	0	0	0	0	0	0	0	0	1	0	0	0	0	0	0	0	0	1	0	0	0	0
Q9BYE4	Small proline-rich protein 2G	SPRR2G	1	3	1	4	1	1	2	1	1	0	1	1	0	0	0	1	0	0	0	0	0	0	0	0	1	0	0	0	0	0
O76094	Signal recognition particle subunit SRP72	SRP72	1	1	1	4	0	0	0	0	1	0	0	0	0	0	0	0	0	1	0	0	0	0	0	0	0	0	1	0	0	0
O43815	Striatin	STRN	1	2	2	4	0	0	0	1	0	0	0	0	0	0	0	0	1	0	0	0	0	0	0	0	0	1	0	0	0	0
Q06520	Bile salt sulfotransferase	SULT2A1	1	1	1	4	0	0	0	1	0	0	0	0	0	0	0	0	1	0	0	0	0	0	0	0	0	1	0	0	0	0
P09493	Tropomyosin alpha-1 chain	TPM1	1	6	1	4	2	4	4	3	3	2	3	2	0	0	0	1	0	0	0	0	0	0	0	0	1	0	0	0	0	0
A6NCI4	von Willebrand factor A domain-containing protein 3A	VWA3A	1	1	1	4	0	0	1	0	0	0	0	0	0	0	0	1	0	0	0	0	0	0	0	0	1	0	0	0	0	0
Q9UIA9	Exportin-7	XPO7	1	3	3	4	0	0	1	0	0	0	0	0	0	0	0	1	0	0	0	0	0	0	0	0	1	0	0	0	0	0
P36578	60S ribosomal protein L4	RPL4	1	10	10	4	4	5	1	1	0	0	0	0	5	4	5	1	1	0	0	0	0	5	4	6	1	1	0	0	0	0
Q9NRX4	14 kDa phosphohistidine phosphatase	PHPT1	1	5	5	4	3	3	2	0	0	0	0	0	4	3	3	2	0	0	0	0	0	5	3	3	2	0	0	0	0	0
P18124	60S ribosomal protein L7	RPL7	1	8	8	4	3	3	0	1	1	0	0	0	3	3	3	0	1	1	0	0	0	3	3	3	0	1	1	0	0	0
P20073	Annexin A7	ANXA7	1	8	8	2	2	2	0	1	1	0	0	0	2	2	2	0	1	1	0	0	0	2	2	2	0	1	1	0	0	0
P01861	Ig gamma-4 chain C region	IGHG4	1	8	2	7	6	6	5	6	6	5	3	4	1	0	1	0	0	0	0	0	0	3	1	2	0	1	1	0	0	0
P40429	60S ribosomal protein L13a	RPL13A	2	4	4	2	1	1	1	0	1	0	0	0	2	1	1	1	0	1	0	0	0	2	1	1	1	0	1	0	0	0
P67870	Casein kinase II subunit beta	CSNK2B	1	2	2	2	1	1	1	1	0	0	0	0	2	1	1	1	1	0	0	0	0	2	1	1	1	1	0	0	0	0
Q02543	60S ribosomal protein L18a	RPL18A	1	2	2	4	1	2	1	0	1	0	0	0	1	1	2	1	0	1	0	0	0	1	1	2	1	0	1	0	0	0
P35268	60S ribosomal protein L22	RPL22	1	4	4	4	2	0	0	1	1	0	0	0	2	2	0	0	1	1	0	0	0	2	2	0	0	1	1	0	0	0
P62750	60S ribosomal protein L23a	RPL23A	1	3	3	4	0	2	1	0	1	0	0	0	2	0	2	1	0	1	0	0	0	2	0	2	1	0	1	0	0	0
A0A0A0MS15	Immunoglobulin heavy variable 3-49	IGHV3-49/-73	4	5	3	2	1	1	1	0	1	0	0	0	1	1	1	1	0	1	0	0	0	1	1	1	1	0	1	0	0	0
Q00341	Vigilin	HDLBP	1	4	4	1	1	1	0	1	1	0	0	0	1	1	1	0	1	1	0	0	0	1	1	1	0	1	1	0	0	0
O14979	Heterogeneous nuclear ribonucleoprotein D-like	HNRNPDL	1	1	1	1	1	1	0	1	1	0	0	0	1	1	1	0	1	1	0	0	0	1	1	1	0	1	1	0	0	0
P62310	U6 snRNA-associated Sm-like protein LSm3	LSM3	1	1	1	1	1	1	1	1	0	0	0	0	1	1	1	1	1	0	0	0	0	1	1	1	1	1	0	0	0	0
Q9P258	Protein RCC2	RCC2	1	3	3	4	1	1	0	1	1	0	0	0	1	1	1	0	1	1	0	0	0	1	1	1	0	1	1	0	0	0
P62277	40S ribosomal protein S13	RPS13	1	5	5	4	1	0	0	1	1	0	0	0	2	1	0	0	1	1	0	0	0	2	1	0	0	1	1	0	0	0
Q9H2U2	Inorganic pyrophosphatase 2, mitochondrial	PPA2	1	7	7	4	1	0	1	0	1	0	0	0	1	1	0	1	0	1	0	0	0	1	1	0	1	0	1	0	0	0

Q9NZD2	Glycolipid transfer protein	GLTP	1	3	3	0	0	0	2	2	1	0	0	0	0	0	0	2	2	1	0	0	0	0	0	0	2	2	1	0	0	0
O75146	Huntingtin-interacting protein 1-related protein	HIP1R	1	3	3	0	0	0	2	2	1	0	0	0	0	0	0	2	2	1	0	0	0	0	0	0	2	2	1	0	0	0
Q92945	Far upstream element-binding protein 2	KHSRP	1	2	2	0	0	0	2	1	2	0	0	0	0	0	0	2	1	2	0	0	0	0	0	0	2	1	2	0	0	0
O95867	Lymphocyte antigen 6 complex locus protein G6c	LY6G6C	1	1	1	0	0	0	1	1	1	0	0	0	0	0	0	1	1	1	0	0	0	0	0	0	2	1	2	0	0	0
Q9GZN4	Brain-specific serine protease 4	PRSS22	1	3	3	4	0	0	2	1	2	0	0	0	0	0	0	2	1	2	0	0	0	0	0	0	2	1	2	0	0	0
P04843	Dolichyl-diphosphooligosaccharide--protein glycosyltransferase subunit 1	RPN1	1	17	17	4	0	0	2	2	1	0	0	0	0	0	0	2	2	1	0	0	0	0	0	0	2	2	1	0	0	0
Q96P63	Serpin B12	SERPINB12	1	3	3	4	0	0	1	3	1	0	0	0	0	0	0	1	3	1	0	0	0	0	0	0	1	3	1	0	0	0
Q07955	Serine/arginine-rich splicing factor 1	SRSF1	1	4	4	4	0	0	1	2	2	0	0	0	0	0	0	1	2	2	0	0	0	0	0	0	1	2	2	0	0	0
Q9UDY2	Tight junction protein ZO-2	TJP2	1	8	8	4	0	0	2	1	2	0	0	0	0	0	0	2	1	2	0	0	0	0	0	0	2	1	2	0	0	0
P45880	Voltage-dependent anion-selective channel protein 2	VDAC2	1	8	8	4	0	0	1	1	2	0	0	0	0	0	0	1	1	2	0	0	0	0	0	0	1	1	3	0	0	0
P12956	X-ray repair cross-complementing protein 6	XRCC6	1	14	14	4	0	0	2	2	1	0	0	0	0	0	0	2	2	1	0	0	0	0	0	0	2	2	1	0	0	0
P62269	40S ribosomal protein S18	RPS18	1	4	4	4	0	1	2	2	2	0	0	0	0	0	1	2	2	2	0	0	0	0	0	1	2	2	2	0	0	0
O00571	ATP-dependent RNA helicase DDX3X	DDX3X;DDX3Y	2	8	8	0	0	0	2	2	2	0	0	0	0	0	0	2	2	2	0	0	0	0	0	0	2	2	2	0	0	0
P02649	Apolipoprotein E	APOE	2	11	11	0	0	0	2	2	2	0	0	0	0	0	0	2	2	2	0	0	0	0	0	0	2	2	2	0	0	0
Q9UI42	Carboxypeptidase A4	CPA4	1	4	4	0	0	0	2	2	2	0	0	0	0	0	0	2	2	2	0	0	0	0	0	0	2	2	2	0	0	0
Q01581	Hydroxymethylglutaryl-CoA synthase, cytoplasmic	HMGCS1	1	4	4	0	0	0	2	3	1	0	0	0	0	0	0	2	3	1	0	0	0	0	0	0	2	3	1	0	0	0
P20700	Lamin-B1	LMNB1	1	9	8	0	0	0	4	0	2	0	0	0	0	0	0	4	0	1	0	0	0	0	0	0	4	0	2	0	0	0
Q99536	Synaptic vesicle membrane protein VAT-1 homolog	VAT1	1	8	8	4	0	0	2	2	2	0	0	0	0	0	0	2	2	2	0	0	0	0	0	0	2	2	2	0	0	0
Q5T750	Skin-specific protein 32	XP32	1	3	3	4	0	0	2	2	2	0	0	0	0	0	0	2	2	2	0	0	0	0	0	0	2	2	2	0	0	0
O43399	Tumor protein D54	TPD52L2	1	5	5	4	3	3	3	2	2	0	0	0	5	3	3	3	2	2	0	0	0	5	3	3	3	2	2	0	0	0
Q9C0C2	182 kDa tankyrase-1-binding protein	TNKS1BP1	1	10	10	4	2	2	4	1	2	0	0	0	2	2	2	4	1	2	0	0	0	2	2	2	4	1	2	0	0	0
P63151	Serine/threonine-protein phosphatase 2A 55 kDa regulatory subunit B alpha isoform	PPP2R2A	4	13	13	2	0	1	3	1	3	0	0	0	2	0	1	3	1	3	0	0	0	2	0	1	3	1	3	0	0	0
P12532	Creatine kinase U-type, mitochondrial	CKMT1A	1	6	6	0	1	0	2	2	3	0	0	0	0	1	0	2	2	3	0	0	0	0	1	0	2	2	3	0	0	0
O60506	Heterogeneous nuclear ribonucleoprotein Q	SYNCRIP	1	9	6	4	0	0	2	1	4	0	0	0	1	0	0	1	0	2	0	0	0	1	0	0	2	1	4	0	0	0
P38646	Stress-70 protein, mitochondrial	HSPA9	1	20	20	0	0	0	2	3	2	0	0	0	0	0	0	2	3	2	0	0	0	0	0	0	2	3	2	0	0	0
Q14210	Lymphocyte antigen 6D	LY6D	1	2	2	0	0	0	2	1	1	0	0	0	0	0	0	2	1	1	0	0	0	0	0	0	3	2	2	0	0	0
P62333	26S protease regulatory subunit 10B	PSMC6	1	10	10	4	0	0	3	2	2	0	0	0	0	0	0	3	2	2	0	0	0	0	0	0	3	2	2	0	0	0
O15020	Spectrin beta chain, non-erythrocytic 2	SPTBN2	1	11	11	4	0	0	2	2	3	0	0	0	0	0	0	2	2	3	0	0	0	0	0	0	2	2	3	0	0	0
P84103	Serine/arginine-rich splicing factor 3	SRSF3	1	4	4	4	0	0	1	3	3	0	0	0	0	0	0	1	3	3	0	0	0	0	0	0	1	3	3	0	0	0
P09497	Clathrin light chain B	CLTB	1	4	4	3	1	2	3	1	4	0	0	0	3	1	2	3	1	4	0	0	0	3	1	2	3	1	4	0	0	0
P06748	Nucleophosmin	NPM1	1	5	5	4	1	1	2	2	1	0	0	0	1	1	1	2	2	1	0	0	0	1	1	1	3	3	2	0	0	0
P61978	Heterogeneous nuclear ribonucleoprotein K	HNRNPK	1	10	10	0	0	0	3	2	3	0	0	0	0	0	0	3	2	3	0	0	0	0	0	0	3	2	3	0	0	0
Q9UKR3	Kallikrein-13	KLK13	1	4	4	0	0	0	3	3	2	0	0	0	0	0	0	3	3	2	0	0	0	0	0	0	3	3	2	0	0	0
Q9UPN3	Microtubule-actin cross-linking factor 1, isoforms 1/2/3/5	MACF1	1	6	6	0	0	0	2	2	4	0	0	0	0	0	0	2	2	4	0	0	0	0	0	0	2	2	4	0	0	0
P54920	Alpha-soluble NSF attachment protein	NAPA	1	7	7	4	0	0	3	2	3	0	0	0	0	0	0	3	2	3	0	0	0	0	0	0	3	2	3	0	0	0
O75635	Serpin B7	SERPINB7	1	3	3	4	0	0	2	3	2	0	0	0	0	0	0	2	3	2	0	0	0	0	0	0	2	3	3	0	0	0
Q9BQ50	Three prime repair exonuclease 2	TREX2	1	4	4	4	0	0	3	3	2	0	0	0	0	0	0	3	3	2	0	0	0	0	0	0	3	3	2	0	0	0
P46782	40S ribosomal protein S5	RPS5	1	6	6	4	3	2	3	2	4	0	0	0	3	3	2	3	2	4	0	0	0	3	3	2	3	2	4	0	0	0
P23396	40S ribosomal protein S3	RPS3	1	9	9	4	2	0	3	3	3	0	0	0	1	2	0	3	3	3	0	0	0	1	2	0	3	3	3	0	0	0
Q9Y4K1	Absent in melanoma 1 protein	AIM1	1	5	5	0	0	1	3	3	3	0	0	0	0	0	1	3	3	3	0	0	0	0	0	1	3	3	3	0	0	0
Q5D862	Filaggrin-2	FLG2	2	5	5	0	0	0	3	3	3	0	0	0	0	0	0	3	3	3	0	0	0	0	0	0	3	3	3	0	0	0
P30622	CAP-Gly domain-containing linker protein 1	CLIP1	2	5	5	0	0	0	4	4	1	0	0	0	0	0	0	4	4	1	0	0	0	0	0	0	4	4	1	0	0	0
Q92841	Probable ATP-dependent RNA helicase DDX17	DDX17	1	4	4	0	0	0	3	3	3	0	0	0	0	0	0	3	3	3	0	0	0	0	0	0	3	3	3	0	0	0
O75367	Core histone macro-H2A.1	H2AFY	1	3	3	0	0	0	3	3	3	0	0	0	0	0	0	3	3	3	0	0	0	0	0	0	3	3	3	0	0	0
P49862	Kallikrein-7	KLK7	1	4	4	0	0	0	3	4	2	0	0	0	0	0	0	3	4	2	0	0	0	0	0	0	3	4	2	0	0	0
Q9H1E1	Ribonuclease 7	RNASE7	1	3	3	4	0	0	3	3	3	0	0	0	0	0	0	3	3	3	0	0	0	0	0	0	3	3	3	0	0	0
P22735	Protein-glutamine gamma-glutamyltransferase K	TGM1	1	4	4	4	0	0	3	3	3	0	0	0	0	0	0	3	3	3	0	0	0	0	0	0	3	3	3	0	0	0
P09525	Annexin A4	ANXA4	1	21	21	12	14	13	3	2	5	0	0	0	12	14	13	3	2	5	0	0	0	12	15	13	3	2	5	0	0	0
Q6E0U4	Dermokine	DMKN	2	3	1	0	0	0	3	3	3	0	0	0	0	0	0	1	1	1	0	0	0	0	0	0	3	3	4	0	0	0
P09601	Heme oxygenase 1	HMOX1	1	4	4	0	0	0	4	3	3	0	0	0	0	0	0	4	3	3	0	0	0	0	0	0	4	3	3	0	0	0

O75131	Copine-3	CPNE3	1	13	13	0	0	1	0	1	1	0	0	0	0	0	1	0	1	1	0	0	0	0	0	1	0	1	1	0	0	0
Q92499	ATP-dependent RNA helicase DDX1	DDX1	1	12	12	1	0	0	1	0	1	0	0	0	1	0	0	1	0	1	0	0	0	1	0	0	1	0	1	0	0	0
Q71DI3	Histone H3.2	HIST2H3A	1	5	1	0	0	1	0	0	2	0	0	0	0	0	0	0	0	0	0	0	0	0	0	1	0	0	2	0	0	0
P27635	60S ribosomal protein L10	RPL10	1	3	3	4	0	0	1	1	0	0	0	0	1	0	0	1	1	0	0	0	0	1	0	0	1	1	0	0	0	0
P62280	40S ribosomal protein S11	RPS11	1	4	4	4	0	1	1	1	0	0	0	0	0	0	1	1	1	0	0	0	0	0	0	1	1	1	0	0	0	0
P15880	40S ribosomal protein S2	RPS2	1	5	5	4	0	0	0	1	1	0	0	0	1	0	0	0	1	1	0	0	0	1	0	0	0	1	1	0	0	0
Q99961	Endophilin-A2	SH3GL1	1	3	3	4	0	1	1	0	1	0	0	0	0	0	1	1	0	1	0	0	0	0	0	1	1	0	1	0	0	0
Q13510	Acid ceramidase	ASAH1	1	6	6	0	0	0	1	1	0	0	0	0	0	0	0	1	1	0	0	0	0	0	0	0	1	1	0	0	0	0
O75947	ATP synthase subunit d, mitochondrial	ATP5H	1	6	6	0	0	0	1	0	1	0	0	0	0	0	0	1	0	1	0	0	0	0	0	0	1	0	1	0	0	0
P61923	Coatomer subunit zeta-1	COPZ1	1	4	4	0	0	0	1	0	1	0	0	0	0	0	0	1	0	1	0	0	0	0	0	0	1	0	1	0	0	0
P14854	Cytochrome c oxidase subunit 6B1	COX6B1	1	4	4	0	0	0	1	1	0	0	0	0	0	0	0	1	1	0	0	0	0	0	0	0	1	1	0	0	0	0
P00167	Cytochrome b5	CYB5A	1	4	4	0	0	0	1	1	0	0	0	0	0	0	0	1	1	0	0	0	0	0	0	0	1	1	0	0	0	0
P36957	Dihydrolipoyllysine-residue succinyltransferase component of 2-oxoglutarate dehydrogenase complex, mitochondrial	DLST	1	4	4	0	0	0	0	1	1	0	0	0	0	0	0	0	1	1	0	0	0	0	0	0	0	1	1	0	0	0
O00303	Eukaryotic translation initiation factor 3 subunit F	EIF3F	1	1	1	0	0	0	1	0	1	0	0	0	0	0	0	1	0	1	0	0	0	0	0	0	1	0	1	0	0	0
Q96AE4	Far upstream element-binding protein 1	FUBP1	1	2	2	0	0	0	1	0	1	0	0	0	0	0	0	1	0	1	0	0	0	0	0	0	1	0	1	0	0	0
A0A075B6J9	Immunoglobulin lambda variable 2-18	IGLV2-18	1	1	1	0	0	0	1	1	0	0	0	0	0	0	0	1	1	0	0	0	0	0	0	0	1	1	0	0	0	0
O00515	Ladinin-1	LAD1	1	1	1	0	0	0	1	1	0	0	0	0	0	0	0	1	1	0	0	0	0	0	0	0	1	1	0	0	0	0
Q3ZCW2	Galectin-related protein	LGALSL	1	2	2	0	0	0	1	0	1	0	0	0	0	0	0	1	0	1	0	0	0	0	0	0	1	0	1	0	0	0
O15173	Membrane-associated progesterone receptor component 2	PGRMC2	1	3	3	4	0	0	1	0	1	0	0	0	0	0	0	1	0	1	0	0	0	0	0	0	1	0	1	0	0	0
P35998	26S protease regulatory subunit 7	PSMC2	1	11	11	4	0	0	1	0	1	0	0	0	0	0	0	1	0	1	0	0	0	0	0	0	1	0	1	0	0	0
P43686	26S protease regulatory subunit 6B	PSMC4	1	8	8	4	0	0	0	1	1	0	0	0	0	0	0	0	1	1	0	0	0	0	0	0	0	1	1	0	0	0
Q99460	26S proteasome non-ATPase regulatory subunit 1	PSMD1	1	8	8	4	0	0	0	1	1	0	0	0	0	0	0	0	1	1	0	0	0	0	0	0	0	1	1	0	0	0
Q15008	26S proteasome non-ATPase regulatory subunit 6	PSMD6	1	9	9	4	0	0	0	1	1	0	0	0	0	0	0	0	1	1	0	0	0	0	0	0	0	1	1	0	0	0
P61289	Proteasome activator complex subunit 3	PSME3	1	1	1	4	0	0	0	1	1	0	0	0	0	0	0	0	1	1	0	0	0	0	0	0	0	1	1	0	0	0
P20339	Ras-related protein Rab-5A	RAB5A	1	4	1	4	0	0	1	1	0	0	0	0	0	0	0	1	1	0	0	0	0	0	0	0	1	1	0	0	0	0
P04844	Dolichyl-diphosphooligosaccharide--protein glycosyltransferase subunit 2	RPN2	1	9	9	4	0	0	0	0	2	0	0	0	0	0	0	0	0	2	0	0	0	0	0	0	0	0	2	0	0	0
P62244	40S ribosomal protein S15a	RPS15A	1	3	3	4	0	0	1	1	0	0	0	0	0	0	0	1	1	0	0	0	0	0	0	0	1	1	0	0	0	0
P42677	40S ribosomal protein S27	RPS27	1	1	1	4	0	0	1	0	1	0	0	0	0	0	0	1	0	1	0	0	0	0	0	0	1	0	1	0	0	0
Q9Y3I0	tRNA-splicing ligase RtcB homolog	RTCB	1	9	9	4	0	0	1	0	1	0	0	0	0	0	0	1	0	1	0	0	0	0	0	0	1	0	1	0	0	0
Q15020	Squamous cell carcinoma antigen recognized by T-cells 3	SART3	1	1	1	4	0	0	1	1	0	0	0	0	0	0	0	1	1	0	0	0	0	0	0	0	1	1	0	0	0	0
P53992	Protein transport protein Sec24C	SEC24C	1	2	2	4	0	0	1	1	0	0	0	0	0	0	0	1	1	0	0	0	0	0	0	0	1	1	0	0	0	0
Q92599	Septin-8	SEPTIN8	1	5	5	4	0	0	1	0	1	0	0	0	0	0	0	1	0	1	0	0	0	0	0	0	1	0	1	0	0	0
Q9UIV8	Serpin B13	SERPINB13	1	2	2	4	0	0	0	0	2	0	0	0	0	0	0	0	0	2	0	0	0	0	0	0	0	0	2	0	0	0
P05141	ADP/ATP translocase 2	SLC25A5	1	6	5	4	0	0	1	0	1	0	0	0	0	0	0	0	0	0	0	0	0	0	0	0	1	0	1	0	0	0
P62316	Small nuclear ribonucleoprotein Sm D2	SNRPD2	1	1	1	4	0	0	1	0	1	0	0	0	0	0	0	1	0	1	0	0	0	0	0	0	1	0	1	0	0	0
Q9NQ38	Serine protease inhibitor Kazal-type 5	SPINK5	1	2	2	4	0	0	1	1	0	0	0	0	0	0	0	1	1	0	0	0	0	0	0	0	1	1	0	0	0	0
P22532	Small proline-rich protein 2D	SPRR2D	1	4	1	4	1	1	2	3	2	0	1	1	0	0	0	0	1	1	0	0	0	0	0	0	0	1	1	0	0	0
Q13885	Tubulin beta-2A chain	TUBB2A	1	20	1	4	2	2	8	6	8	11	9	10	0	0	0	1	0	1	0	0	0	0	0	0	1	0	1	0	0	0
P26368	Splicing factor U2AF 65 kDa subunit	U2AF2	1	1	1	4	0	0	1	0	1	0	0	0	0	0	0	1	0	1	0	0	0	0	0	0	1	0	1	0	0	0
Q8TCV5	WAP four-disulfide core domain protein 5	WFDC5	1	1	1	4	0	0	1	0	1	0	0	0	0	0	0	1	0	1	0	0	0	0	0	0	1	0	1	0	0	0
P13010	X-ray repair cross-complementing protein 5	XRCC5	1	14	14	4	0	0	1	0	1	0	0	0	0	0	0	1	0	1	0	0	0	0	0	0	1	0	1	0	0	0
P60903	Protein S100-A10	S100A10	1	6	6	4	4	4	1	1	1	0	0	0	3	4	4	1	1	1	0	0	0	4	4	4	1	1	1	0	0	0
P02747	Complement C1q subcomponent subunit C	C1QC	1	4	4	3	1	1	1	1	1	0	0	0	3	1	1	1	1	1	0	0	0	3	1	1	1	1	1	0	0	0
O75828	Carbonyl reductase [NADPH] 3	CBR3	1	8	6	3	4	4	3	3	3	2	2	2	1	2	2	1	1	1	0	0	0	1	2	2	1	1	1	0	0	0
Q15075	Early endosome antigen 1	EEA1	1	9	9	2	2	1	1	2	0	0	0	0	2	2	1	1	2	0	0	0	0	2	2	1	1	2	0	0	0	0
Q8NC51	Plasminogen activator inhibitor 1 RNA-binding protein	SERBP1	1	4	4	4	2	2	1	1	1	0	0	0	1	2	2	1	1	1	0	0	0	1	2	2	1	1	1	0	0	0
P50452	Serpin B8	SERPINB8	1	4	2	4	3	2	2	2	2	1	1	1	1	1	0	1	1	1	0	0	0	2	2	1	1	1	1	0	0	0
P07910	Heterogeneous nuclear ribonucleoproteins C1/C2	PCL4;HNRNPCL1;HNRNP	7	5	5	1	2	1	1	1	1	0	0	0	1	2	1	1	1	1	0	0	0	1	2	1	1	1	1	0	0	0
Q8WWI1	LIM domain only protein 7	LMO7	1	8	8	2	1	1	1	1	1	0	0	0	2	1	1	1	1	1	0	0	0	2	1	1	1	1	1	0	0	0
P55209	Nucleosome assembly protein 1-like 1	NAP1L1	1	5	3	4	3	1	1	1	1	2	2	2	2	1	1	1	1	1	0	0	0	2	1	1	1	1	1	0	0	0

Q9C002	Normal mucosa of esophagus-specific gene 1 protein	NMES1	1	3	3	4	0	0	1	1	1	0	0	0	0	0	0	1	1	1	0	0	0	0	0	0	1	1	1	0	0	0
Q9NRA1	Platelet-derived growth factor C	PDGFC	1	3	3	4	0	0	1	1	1	0	0	0	0	0	0	1	1	1	0	0	0	0	0	0	1	1	1	0	0	0
Q3MJ16	Cytosolic phospholipase A2 epsilon	PLA2G4E	1	1	1	4	0	0	1	1	1	0	0	0	0	0	0	1	1	1	0	0	0	0	0	0	1	1	1	0	0	0
P62829	60S ribosomal protein L23	RPL23	1	3	3	4	0	0	1	1	1	0	0	0	0	0	0	1	1	1	0	0	0	0	0	0	1	1	1	0	0	0
P32969	60S ribosomal protein L9	RPL9	1	7	7	4	0	0	1	1	1	0	0	0	0	0	0	1	1	1	0	0	0	0	0	0	1	1	1	0	0	0
P62263	40S ribosomal protein S14	RPS14	1	4	4	4	0	0	1	2	0	0	0	0	0	0	0	1	2	0	0	0	0	0	0	0	1	2	0	0	0	0
Q00325	Phosphate carrier protein, mitochondrial	SLC25A3	1	1	1	4	0	0	1	1	1	0	0	0	0	0	0	1	1	1	0	0	0	0	0	0	1	1	1	0	0	0
P55000	Secreted Ly-6/uPAR-related protein 1	SLURP1	1	1	1	4	0	0	1	1	1	0	0	0	0	0	0	1	1	1	0	0	0	0	0	0	1	1	1	0	0	0
P35325	Small proline-rich protein 2B	SPRR2B	1	5	0	4	1	1	4	4	3	0	1	1	0	0	0	0	0	0	0	0	0	0	0	0	1	2	0	0	0	0
Q96RM1	Small proline-rich protein 2F	SPRR2F	1	2	1	4	0	0	2	2	2	0	0	0	0	0	0	1	1	1	0	0	0	0	0	0	1	1	1	0	0	0
Q9Y6N5	Sulfide:quinone oxidoreductase, mitochondrial	SQRDL	1	17	17	4	0	0	1	1	1	0	0	0	0	0	0	1	1	1	0	0	0	0	0	0	1	1	1	0	0	0
Q9Y5M8	Signal recognition particle receptor subunit beta	SRPRB	1	2	2	4	0	0	1	1	1	0	0	0	0	0	0	1	1	1	0	0	0	0	0	0	1	1	1	0	0	0
Q6NXT6	Transmembrane anterior posterior transformation protein 1 homolog	TAPT1	1	2	2	4	0	0	1	1	1	0	0	0	0	0	0	1	1	1	0	0	0	0	0	0	1	1	1	0	0	0
P49755	Transmembrane emp24 domain-containing protein 10	TMED10	1	6	6	4	0	0	1	1	1	0	0	0	0	0	0	1	1	1	0	0	0	0	0	0	1	1	1	0	0	0
Q9BUF5	Tubulin beta-6 chain	TUBB6	1	7	2	4	1	1	3	3	4	3	3	3	0	0	0	1	1	1	0	0	0	0	0	0	1	1	1	0	0	0
P07919	Cytochrome b-c1 complex subunit 6, mitochondrial	UQCRH	1	1	1	4	0	0	1	1	1	0	0	0	0	0	0	1	1	1	0	0	0	0	0	0	1	1	1	0	0	0
P62857	40S ribosomal protein S28	RPS28	1	6	6	4	4	3	1	2	1	0	0	0	3	4	3	1	2	1	0	0	0	3	4	3	1	2	1	0	0	0
P16989	Y-box-binding protein 3	YBX3	1	8	5	4	3	3	1	1	2	0	0	0	3	3	2	1	1	1	0	0	0	4	3	3	1	1	2	0	0	0
P04003	C4b-binding protein alpha chain	C4BPA	1	12	12	3	3	3	1	2	1	0	0	0	3	3	3	1	2	1	0	0	0	3	3	3	1	2	1	0	0	0
P41250	Glycine--tRNA ligase	GARS	1	9	9	2	2	5	1	1	2	0	0	0	2	2	5	1	1	2	0	0	0	2	2	5	1	1	2	0	0	0
Q13347	Eukaryotic translation initiation factor 3 subunit I	EIF3I	1	6	6	2	1	2	2	1	1	0	0	0	2	1	2	2	1	1	0	0	0	2	1	2	2	1	1	0	0	0
P27816	Microtubule-associated protein 4	MAP4	1	6	6	2	2	1	1	1	2	0	0	0	2	2	1	1	1	2	0	0	0	2	2	1	1	1	2	0	0	0
Q96EP5	DAZ-associated protein 1	DAZAP1	1	4	4	1	1	1	2	1	1	0	0	0	1	1	1	2	1	1	0	0	0	1	1	1	2	1	1	0	0	0
P48163	NADP-dependent malic enzyme	ME1	1	4	4	1	1	1	1	1	1	0	0	0	1	1	1	1	1	1	0	0	0	1	1	1	2	1	1	0	0	0
O76003	Glutaredoxin-3	GLRX3	1	7	7	0	1	1	1	2	1	0	0	0	0	1	1	1	2	1	0	0	0	0	1	1	1	2	1	0	0	0
Q14258	E3 ubiquitin/ISG15 ligase TRIM25	TRIM25	1	6	6	4	0	1	1	2	1	0	0	0	1	0	1	1	2	1	0	0	0	1	0	1	1	2	1	0	0	0
P61604	10 kDa heat shock protein, mitochondrial	HSPE1	1	3	3	0	0	1	1	1	2	0	0	0	0	0	1	1	1	2	0	0	0	0	0	1	1	1	2	0	0	0
P16615	Sarcoplasmic/endoplasmic reticulum calcium ATPase 2	ATP2A2	2	9	6	0	0	0	2	1	1	0	0	0	0	0	0	1	0	0	0	0	0	0	0	0	2	1	1	0	0	0
Q9BPX5	Actin-related protein 2/3 complex subunit 5-like protein	ARPC5L	1	3	3	0	0	0	1	2	1	0	0	0	0	0	0	1	2	1	0	0	0	0	0	0	1	2	1	0	0	0
P27824	Calnexin	CANX	1	10	10	0	0	0	1	2	1	0	0	0	0	0	0	1	2	1	0	0	0	0	0	0	1	2	1	0	0	0
P20674	Cytochrome c oxidase subunit 5A, mitochondrial	COX5A	1	5	5	0	0	0	2	1	1	0	0	0	0	0	0	2	1	1	0	0	0	0	0	0	2	1	1	0	0	0
P30084	Enoyl-CoA hydratase, mitochondrial	ECHS1	1	7	7	0	0	0	2	1	1	0	0	0	0	0	0	2	1	1	0	0	0	0	0	0	2	1	1	0	0	0
Q9Y262	Eukaryotic translation initiation factor 3 subunit L	EIF3L	1	4	4	0	0	0	2	1	1	0	0	0	0	0	0	2	1	1	0	0	0	0	0	0	2	1	1	0	0	0
Q01844	RNA-binding protein EWS	EWSR1	1	3	3	0	0	0	1	1	2	0	0	0	0	0	0	1	1	2	0	0	0	0	0	0	1	1	2	0	0	0
Q7L5L3	Glycerophosphodiester phosphodiesterase domain-containing protein 3	GDPD3	1	2	2	0	0	0	1	1	2	0	0	0	0	0	0	1	1	2	0	0	0	0	0	0	1	1	2	0	0	0
P51659	Peroxisomal multifunctional enzyme type 2	HSD17B4	1	16	16	0	0	0	2	0	2	0	0	0	0	0	0	2	0	2	0	0	0	0	0	0	2	0	2	0	0	0
Q6ZNF0	Iron/zinc purple acid phosphatase-like protein	PAPL	1	2	2	4	0	0	1	2	1	0	0	0	0	0	0	1	2	1	0	0	0	0	0	0	1	2	1	0	0	0
P23246	Splicing factor, proline- and glutamine-rich	SFPQ	1	4	4	4	0	0	1	1	2	0	0	0	0	0	0	1	1	2	0	0	0	0	0	0	1	1	2	0	0	0
Q9Y5X1	Sorting nexin-9	SNX9	1	2	2	4	0	0	2	1	1	0	0	0	0	0	0	2	1	1	0	0	0	0	0	0	2	1	1	0	0	0
Q9Y3C8	Ubiquitin-fold modifier-conjugating enzyme 1	UFC1	1	2	2	4	0	0	1	1	2	0	0	0	0	0	0	1	1	2	0	0	0	0	0	0	1	1	2	0	0	0
Q92598	Heat shock protein 105 kDa	HSPH1	2	7	6	3	2	2	1	3	1	0	0	1	2	1	1	1	3	1	0	0	0	2	1	1	1	3	1	0	0	0
O95817	BAG family molecular chaperone regulator 3	BAG3	1	4	4	1	2	1	1	2	2	0	0	0	1	2	1	1	2	2	0	0	0	1	2	1	1	2	2	0	0	0
Q99880	Histone H2B type 1-L	BC;HIST1H2BD;H2BFS;H	15	4	4	1	1	0	1	1	3	0	0	0	1	1	0	1	1	3	0	0	0	1	1	0	1	1	3	0	0	0
P16401	Histone H1.5	HIST1H1B	1	5	4	2	0	0	2	2	2	0	0	0	2	0	0	2	2	1	0	0	0	2	0	0	2	2	1	0	0	0
Q9H0E2	Toll-interacting protein	TOLLIP	1	3	3	4	0	0	2	2	1	0	0	0	1	0	0	2	2	1	0	0	0	1	0	0	2	2	1	0	0	0
O76027	Annexin A9	ANXA9	1	13	13	0	0	0	2	1	2	0	0	0	0	0	0	2	1	2	0	0	0	0	0	0	2	1	2	0	0	0
Q9NVJ2	ADP-ribosylation factor-like protein 8B	ARL8B	1	4	1	0	0	0	2	1	2	0	0	0	0	0	0	1	1	1	0	0	0	0	0	0	2	1	2	0	0	0
P21128	Poly(U)-specific endoribonuclease	ENDOU	1	2	2	0	0	0	2	2	1	0	0	0	0	0	0	2	2	1	0	0	0	0	0	0	2	2	1	0	0	0
Q86WN1	F-BAR and double SH3 domains protein 1	FCHSD1	1	2	2	0	0	0	2	2	1	0	0	0	0	0	0	2	2	1	0	0	0	0	0	0	2	2	1	0	0	0
P02751	Fibronectin	FN1	1	12	12	0	0	0	2	1	2	0	0	0	0	0	0	2	1	2	0	0	0	0	0	0	2	1	2	0	0	0

P49643	DNA primase large subunit	PRIM2	1	1	1	4	1	1	1	1	1	0	0	0	1	1	1	1	1	1	0	0	0	2	1	1	1	1	1	0	0	0
P20340	Ras-related protein Rab-6A	RAB6A;RAB6B	3	4	4	1	1	1	1	1	1	0	0	0	1	1	1	1	1	1	0	0	0	1	1	1	1	1	1	0	0	0
Q9HC35	Echinoderm microtubule-associated protein-like 4	EML4	1	3	3	1	1	1	1	1	1	0	0	0	1	1	1	1	1	1	0	0	0	1	1	1	1	1	1	0	0	0
Q16658	Fascin	FSCN1	1	1	1	1	1	1	1	1	1	0	0	0	1	1	1	1	1	1	0	0	0	1	1	1	1	1	1	0	0	0
P14866	Heterogeneous nuclear ribonucleoprotein L	HNRNPL	1	8	8	1	1	1	1	1	1	0	0	0	1	1	1	1	1	1	0	0	0	1	1	1	1	1	1	0	0	0
Q969H8	Myeloid-derived growth factor	MYDGF	1	2	2	4	0	1	1	1	1	0	0	0	2	0	1	1	1	1	0	0	0	2	0	1	1	1	1	0	0	0
P28074	Proteasome subunit beta type-5	PSMB5	1	1	1	4	1	1	1	1	1	0	0	0	1	1	1	1	1	1	0	0	0	1	1	1	1	1	1	0	0	0
Q09028	Histone-binding protein RBBP4	RBBP4	1	1	1	4	1	1	1	1	1	0	0	0	1	1	1	1	1	1	0	0	0	1	1	1	1	1	1	0	0	0
P62913	60S ribosomal protein L11	RPL11	1	2	2	4	1	1	1	1	1	0	0	0	1	1	1	1	1	1	0	0	0	1	1	1	1	1	1	0	0	0
P61513	60S ribosomal protein L37a	RPL37A	1	1	1	4	1	1	1	1	1	0	0	0	1	1	1	1	1	1	0	0	0	1	1	1	1	1	1	0	0	0
P0C0L5	Complement C4-B	C4B	1	60	2	7	8	8	15	13	16	5	4	5	1	1	0	1	1	1	0	0	0	1	1	0	1	1	1	0	0	0
A0A0C4DH36	Ig-like domain-containing protein	IGHV3-38	1	3	1	1	1	0	1	1	1	0	0	0	1	1	0	1	1	1	0	0	0	1	1	0	1	1	1	0	0	0
Q9Y4Y9	U6 snRNA-associated Sm-like protein LSm5	LSM5	1	1	1	1	0	1	1	1	1	0	0	0	1	0	1	1	1	1	0	0	0	1	0	1	1	1	1	0	0	0
P55145	Mesencephalic astrocyte-derived neurotrophic factor	MANF	1	1	1	0	1	1	1	1	1	0	0	0	0	1	1	1	1	1	0	0	0	0	1	1	1	1	1	0	0	0
O75608	Acyl-protein thioesterase 1	LYPLA1	1	6	6	1	0	0	1	1	1	0	0	0	1	0	0	1	1	1	0	0	0	1	0	0	1	1	1	0	0	0
P17980	26S protease regulatory subunit 6A	PSMC3	1	5	5	4	0	1	1	1	1	0	0	0	0	0	1	1	1	1	0	0	0	0	0	1	1	1	1	0	0	0
Q8WZ42	Titin	TTN	1	6	6	4	1	0	0	1	2	0	0	0	0	1	0	0	1	2	0	0	0	0	1	0	0	1	2	0	0	0
Q13838	Spliceosome RNA helicase DDX39B	DDX39B;DDX39A	2	12	12	0	0	0	1	1	1	0	0	0	0	0	0	1	1	1	0	0	0	0	0	0	1	1	1	0	0	0
P50570	Dynamin-2	DNM2	2	7	7	0	0	0	1	1	1	0	0	0	0	0	0	1	1	1	0	0	0	0	0	0	1	1	1	0	0	0
Q9H4M9	EH domain-containing protein 1	EHD1	2	3	2	0	0	0	1	1	1	0	0	0	0	0	0	1	1	1	0	0	0	0	0	0	1	1	1	0	0	0
P35637	RNA-binding protein FUS	FUS;TAF15	2	1	1	0	0	0	1	1	1	0	0	0	0	0	0	1	1	1	0	0	0	0	0	0	1	1	1	0	0	0
Q15067	Peroxisomal acyl-coenzyme A oxidase 1	ACOX1	1	2	2	0	0	0	2	0	1	0	0	0	0	0	0	2	0	1	0	0	0	0	0	0	2	0	1	0	0	0
Q53RT3	Retroviral-like aspartic protease 1	ASPRV1	1	2	2	0	0	0	1	1	1	0	0	0	0	0	0	1	1	1	0	0	0	0	0	0	1	1	1	0	0	0
Q8NEY4	V-type proton ATPase subunit C 2	ATP6V1C2	1	1	1	0	0	0	1	1	1	0	0	0	0	0	0	1	1	1	0	0	0	0	0	0	1	1	1	0	0	0
Q07021	Complement component 1 Q subcomponent-binding protein, mitochondrial	C1QBP	1	4	4	0	0	0	1	1	1	0	0	0	0	0	0	1	1	1	0	0	0	0	0	0	1	1	1	0	0	0
A6NLJ0	C2 calcium-dependent domain-containing protein 4B	C2CD4B	1	1	1	0	0	0	1	1	1	0	0	0	0	0	0	1	1	1	0	0	0	0	0	0	1	1	1	0	0	0
P00918	Carbonic anhydrase 2	CA2	1	2	2	0	0	0	1	1	1	0	0	0	0	0	0	1	1	1	0	0	0	0	0	0	1	1	1	0	0	0
Q8N6Q3	CD177 antigen	CD177	1	1	1	0	0	0	1	1	1	0	0	0	0	0	0	1	1	1	0	0	0	0	0	0	1	1	1	0	0	0
P10606	Cytochrome c oxidase subunit 5B, mitochondrial	COX5B	1	2	2	0	0	0	1	1	1	0	0	0	0	0	0	1	1	1	0	0	0	0	0	0	1	1	1	0	0	0
Q9UGL9	Cysteine-rich C-terminal protein 1	CRCT1	1	2	2	0	0	0	1	2	0	0	0	0	0	0	0	1	2	0	0	0	0	0	0	0	1	2	0	0	0	0
Q15828	Cystatin-M	CST6	1	2	2	0	0	0	1	1	1	0	0	0	0	0	0	1	1	1	0	0	0	0	0	0	1	1	1	0	0	0
P35222	Catenin beta-1	CTNNB1	1	5	3	0	0	0	3	3	3	0	0	0	0	0	0	1	1	1	0	0	0	0	0	0	1	1	1	0	0	0
Q9NSA3	Beta-catenin-interacting protein 1	CTNNBIP1	1	1	1	0	0	0	1	1	1	0	0	0	0	0	0	1	1	1	0	0	0	0	0	0	1	1	1	0	0	0
O15263	Beta-defensin 4A	DEFB4A	1	1	1	0	0	0	1	1	1	0	0	0	0	0	0	1	1	1	0	0	0	0	0	0	1	1	1	0	0	0
Q08211	ATP-dependent RNA helicase A	DHX9	1	6	6	0	0	0	1	1	1	0	0	0	0	0	0	1	1	1	0	0	0	0	0	0	1	1	1	0	0	0
P31689	DnaJ homolog subfamily A member 1	DNAJA1	1	4	4	0	0	0	1	1	1	0	0	0	0	0	0	1	1	1	0	0	0	0	0	0	1	1	1	0	0	0
P63172	Dynein light chain Tctex-type 1	DYNLT1	1	2	2	0	0	0	1	1	1	0	0	0	0	0	0	1	1	1	0	0	0	0	0	0	1	1	1	0	0	0
O15371	Eukaryotic translation initiation factor 3 subunit D	EIF3D	1	7	7	0	0	0	1	1	1	0	0	0	0	0	0	1	1	1	0	0	0	0	0	0	1	1	1	0	0	0
P84090	Enhancer of rudimentary homolog	ERH	1	3	3	0	0	0	1	2	0	0	0	0	0	0	0	1	2	0	0	0	0	0	0	0	1	2	0	0	0	0
Q92820	Gamma-glutamyl hydrolase	GGH	1	3	3	0	0	0	1	2	0	0	0	0	0	0	0	1	2	0	0	0	0	0	0	0	1	2	0	0	0	0
P84243	Histone H3.3	H3F3A	1	5	1	0	0	1	1	1	3	0	0	0	0	0	0	1	1	1	0	0	0	0	0	0	1	1	1	0	0	0
P40939	Trifunctional enzyme subunit alpha, mitochondrial	HADHA	1	21	21	0	0	0	1	1	1	0	0	0	0	0	0	1	1	1	0	0	0	0	0	0	1	1	1	0	0	0
Q9Y241	HIG1 domain family member 1A, mitochondrial	HIGD1A	1	1	1	0	0	0	1	1	1	0	0	0	0	0	0	1	1	1	0	0	0	0	0	0	1	1	1	0	0	0
P17096	High mobility group protein HMG-I/HMG-Y	HMGA1	1	2	2	0	0	0	1	1	1	0	0	0	0	0	0	1	1	1	0	0	0	0	0	0	1	1	1	0	0	0
Q58FF8	Putative heat shock protein HSP 90-beta 2	HSP90AB2P	1	5	1	1	1	1	4	4	4	0	1	1	0	0	0	1	1	1	0	0	0	0	0	0	1	1	1	0	0	0
A0A0C4DH35	Ig-like domain-containing protein	IGHV3-35	1	1	1	0	0	0	1	1	1	0	0	0	0	0	0	1	1	1	0	0	0	0	0	0	1	1	1	0	0	0
P49441	Inositol polyphosphate 1-phosphatase	INPP1	1	3	3	0	0	0	1	1	1	0	0	0	0	0	0	1	1	1	0	0	0	0	0	0	1	1	1	0	0	0
Q07666	KH domain-containing, RNA-binding, signal transduction-associated protein 1	KHDRBS1	1	1	1	0	0	0	1	1	1	0	0	0	0	0	0	1	1	1	0	0	0	0	0	0	1	1	1	0	0	0
Q03252	Lamin-B2	LMNB2	1	5	4	0	0	0	1	2	1	0	0	0	0	0	0	1	2	0	0	0	0	0	0	0	1	2	0	0	0	0
Q92614	Unconventional myosin-XVIIIa	MYO18A	1	1	1	4	0	0	1	1	1	0	0	0	0	0	0	1	1	1	0	0	0	0	0	0	1	1	1	0	0	0

Q86XP0	Cytosolic phospholipase A2 delta	PLA2G4D	1	4	4	4	0	0	4	3	3	0	0	0	0	0	0	4	3	3	0	0	0	0	0	0	4	3	3	0	0	0
Q6NUJ1	Proactivator polypeptide-like 1	PSAPL1	1	5	5	4	0	0	2	4	3	0	0	0	0	0	0	2	4	3	0	0	0	0	0	0	2	4	4	0	0	0
O60218	Aldo-keto reductase family 1 member B10	AKR1B10	2	5	5	0	0	0	3	4	4	0	0	0	0	0	0	3	4	4	0	0	0	0	0	0	3	4	4	0	0	0
P05089	Arginase-1	ARG1	1	5	5	0	0	0	4	3	3	0	0	0	0	0	0	4	3	3	0	0	0	0	0	0	5	3	3	0	0	0
Q86UP2	Kinectin	KTN1	1	7	7	0	0	0	3	5	3	0	0	0	0	0	0	3	5	3	0	0	0	0	0	0	3	5	3	0	0	0
Q9H0P0	Cytosolic 5-nucleotidase 3A	NT5C3A	1	5	5	4	0	0	4	4	3	0	0	0	0	0	0	4	4	3	0	0	0	0	0	0	4	4	3	0	0	0
B3EWG6	Protein FAM25G	AM25G;FAM25C;FAM25	3	3	3	0	0	0	3	3	3	0	0	0	0	0	0	3	3	3	0	0	0	0	0	0	4	4	4	0	0	0
O95833	Chloride intracellular channel protein 3	CLIC3	1	9	9	0	0	0	4	4	4	0	0	0	0	0	0	4	4	4	0	0	0	0	0	0	4	4	4	0	0	0
Q9UBH0	Interleukin-36 receptor antagonist protein	IL36RN	1	4	4	0	0	0	3	2	4	0	0	0	0	0	0	3	2	4	0	0	0	0	0	0	4	3	5	0	0	0
Q9Y3R4	Sialidase-2	NEU2	1	5	5	4	0	0	5	3	4	0	0	0	0	0	0	5	3	4	0	0	0	0	0	0	5	3	4	0	0	0
P0C869	Cytosolic phospholipase A2 beta	PLA2G4B	1	5	5	4	0	0	5	4	4	0	0	0	0	0	0	5	4	4	0	0	0	0	0	0	5	4	4	0	0	0
P19338	Nucleolin	NCL	1	9	9	4	3	3	4	4	3	0	0	0	6	3	3	4	4	3	0	0	0	7	3	3	5	5	4	0	0	0
Q9UKQ9	Kallikrein-9	KLK9	1	5	5	0	0	0	3	3	5	0	0	0	0	0	0	3	3	5	0	0	0	0	0	0	4	4	6	0	0	0
P60985	Keratinocyte differentiation-associated protein	KRTDAP	1	5	5	0	0	0	5	3	3	0	0	0	0	0	0	5	3	3	0	0	0	0	0	0	6	5	4	0	0	0
P10809	60 kDa heat shock protein, mitochondrial	HSPD1	1	16	16	1	0	1	5	4	4	0	0	0	1	0	1	5	4	4	0	0	0	2	0	2	6	5	5	0	0	0
P25705	ATP synthase subunit alpha, mitochondrial	ATP5A1	1	24	24	0	0	0	5	5	3	0	0	0	0	0	0	5	5	3	0	0	0	0	0	0	6	6	4	0	0	0
Q15517	Corneodesmosin	CDSN	1	7	7	0	0	0	6	4	6	0	0	0	0	0	0	6	4	6	0	0	0	0	0	0	6	4	6	0	0	0
Q00839	Heterogeneous nuclear ribonucleoprotein U	HNRNPU	1	11	11	0	0	0	5	5	6	0	0	0	0	0	0	5	5	6	0	0	0	0	0	0	5	5	6	0	0	0
Q9Y446	Plakophilin-3	PKP3	1	7	7	4	0	1	6	5	6	0	0	0	1	0	1	6	5	6	0	0	0	1	0	1	6	5	6	0	0	0
Q96QA5	Gasdermin-A	GSDMA	1	5	5	0	0	0	5	4	5	0	0	0	0	0	0	5	4	5	0	0	0	0	0	0	6	5	6	0	0	0
Q9NZH8	Interleukin-36 gamma	IL36G	1	7	7	0	0	0	7	5	6	0	0	0	0	0	0	7	5	6	0	0	0	0	0	0	7	5	6	0	0	0
Q92876	Kallikrein-6	KLK6	1	7	7	0	0	0	5	4	6	0	0	0	0	0	0	5	4	6	0	0	0	0	0	0	6	5	7	0	0	0
P17900	Ganglioside GM2 activator	GM2A	1	5	5	0	0	0	5	5	5	0	0	0	0	0	0	5	5	5	0	0	0	0	0	0	6	6	7	0	0	0
O43240	Kallikrein-10	KLK10	1	6	6	0	0	0	6	5	5	0	0	0	0	0	0	6	5	5	0	0	0	0	0	0	7	7	7	0	0	0
P62805	Histone H4	HIST1H4A	1	10	10	4	4	4	7	6	7	0	0	0	4	4	4	7	6	7	0	0	0	4	4	4	8	7	8	0	0	0
P12259	Coagulation factor V	F5	1	21	15	0	0	0	8	5	10	0	0	0	0	0	0	4	4	6	0	0	0	0	0	0	8	5	10	0	0	0
P42357	Histidine ammonia-lyase	HAL	1	10	10	0	0	0	8	6	8	0	0	0	0	0	0	8	6	8	0	0	0	0	0	0	9	6	8	0	0	0
P05091	Aldehyde dehydrogenase, mitochondrial	ALDH2	1	16	13	3	1	2	11	8	8	1	1	1	1	0	0	10	7	6	0	0	0	1	0	0	10	7	7	0	0	0
A8K2U0	Alpha-2-macroglobulin-like protein 1	A2ML1	1	11	11	0	0	0	9	8	8	0	0	0	0	0	0	9	8	8	0	0	0	0	0	0	9	8	8	0	0	0
Q14574	Desmocollin-3	DSC3	1	14	14	2	2	1	9	9	10	0	0	0	2	2	1	9	9	10	0	0	0	2	2	1	9	9	10	0	0	0
Q08188	Protein-glutamine gamma-glutamyltransferase E	TGM3	1	12	12	4	0	0	11	9	10	0	0	0	0	0	0	11	9	10	0	0	0	0	0	0	12	9	10	0	0	0
Q5T749	Keratinocyte proline-rich protein	KPRP	1	13	13	0	0	0	10	11	9	0	0	0	0	0	0	10	11	9	0	0	0	0	0	0	10	13	9	0	0	0
Q07065	Cytoskeleton-associated protein 4	CKAP4	1	18	18	0	0	0	11	14	10	0	0	0	0	0	0	11	14	10	0	0	0	0	0	0	11	14	10	0	0	0
P48594	Serpin B4	SERPINB4	1	20	9	4	0	0	18	19	19	0	0	0	0	0	0	8	8	9	0	0	0	0	0	0	11	13	12	0	0	0
P14735	Insulin-degrading enzyme	IDE	1	16	16	3	0	1	12	11	14	0	0	0	3	0	1	12	11	14	0	0	0	3	0	1	13	11	14	0	0	0
Q13835	Plakophilin-1	PKP1	1	16	16	4	0	0	11	9	16	0	0	0	0	0	0	11	9	16	0	0	0	0	0	0	13	9	16	0	0	0
P58107	Epiplakin	EPPK1	1	36	32	0	0	0	19	15	17	0	0	0	0	0	0	15	12	13	0	0	0	0	0	0	16	12	14	0	0	0
Q8WVV4	Protein POF1B	POF1B	1	20	20	4	0	0	16	15	18	0	0	0	0	0	0	16	15	18	0	0	0	0	0	0	20	18	21	0	0	0
P29508	Serpin B3	SERPINB3	1	22	11	4	0	0	21	19	18	0	0	0	0	0	0	11	8	8	0	0	0	0	0	0	26	24	21	0	0	0
Q15149	Plectin	PLEC	1	61	57	4	2	3	26	25	29	0	0	0	1	2	3	22	22	25	0	0	0	1	2	3	26	25	29	0	0	0

Table S2. List of identified proteins with the highest mean of spectral count in the whole strip (W). The proteins are ranked according to their spectral count.

Accession number	Protein name	Gene name	Mean of spectral count
P12273	Prolactin-inducible protein	*PIP*	142.3
P98160	Heparan sulfate proteoglycan 2	*HSPG2*	113.3
P61626	Lysozyme C	*LYZ*	111.3
P01876	Ig alpha-1 chain C region	*IGHA1*	107.0
P02768	Serum albumin	*ALB*	101.7
P02788	Lactotransferrin	*LTF*	100.7
P01833	Polymeric immunoglobulin receptor	*PIGR*	98.7
P01036	Cystatin-S	*CST4*	91.0
P01024	Complement C3	*C3*	72.7
Q16378	Proline-rich protein 4	*PRR4*	71.3
P00450	Ceruloplasmin	*CP*	53.7
Q9UGM3	Deleted in malignant brain tumors 1 protein	*DMBT1*	50.7
Q9GZZ8	Extracellular glycoprotein lacritin	*LACRT*	49.7
P31025	Lipocalin-1	*LCN1*	45.3
P00352	Retinal dehydrogenase 1	*ALDH1A1*	35.3
O75556	Mammaglobin-B	*SCGB2A1*	35.3
P01834	Ig kappa chain C region	*IGKC*	32.3
P06733	Alpha-enolase	*ENO1*	31.3
O43707	Alpha-actinin-4	*ACTN4*	30.7
P01591	Immunoglobulin J chain	*IGJ*	30.7

Table S3. List of identified proteins with the highest mean of spectral count in the bulb (B). The proteins are ranked according to their spectral count.

Accession number	Protein name	Gene name	Mean of spectral count
P02768	Serum albumin	*ALB*	204.3
P02788	Lactotransferrin	*LTF*	130.3
P12273	Prolactin-inducible protein	PIP	108.0
P61626	Lysozyme C	*LYZ*	104.0
P01024	Complement C3	*C3*	69.7

P01876	Ig alpha-1 chain C region	*IGHA1*	61.7
P01833	Polymeric immunoglobulin receptor	*PIGR*	60.0
P02787	Serotransferrin	*TF*	59.0
P98160	Heparan sulfate proteoglycan 2	*HSPG2*	56.3
P98088	Mucin-5AC	*MUC5AC*	54.3
P06733	Alpha-enolase	*ENO1*	52.0
P31025	Lipocalin-1	*LCN1*	50.0
Q9UGM3	Deleted in malignant brain tumors 1 protein	*DMBT1*	48.0
P00352	Retinal dehydrogenase 1	*ALDH1A1*	46.0
P00450	Ceruloplasmin	*CP*	43.3
P01036	Cystatin-S	*CST4*	42.7
P14618	Pyruvate kinase PKM	*PKM*	41.7
P07355	Annexin A2	*ANXA2*	36.7
P0DMV9	Heat shock 70 kDa protein 1B	*HSPA1B*	35.7
P00558	Phosphoglycerate kinase 1	*PGK1*	35.7

Table S4. List of identified proteins with the highest mean of spectral count in the rest of the strip (R). The proteins are ranked according to their spectral count.

Accession number	Protein name	Gene name	Mean of spectral count
P98160	Heparan sulfate proteoglycan 2	*HSPG2*	123.7
P12273	Prolactin-inducible protein	*PIP*	121.7
P61626	Lysozyme C	*LYZ*	108.7
P02788	Lactotransferrin	*LTF*	101.7
P15924	Desmoplakin	*DSP*	97.0
P01833	Polymeric immunoglobulin receptor;	*PIGR*	83.3
P01036	Cystatin-S	*CST4*	76.7
P01876	Ig alpha-1 chain C region	*IGHA1*	74.0
P02768	Serum albumin	*ALB*	67.0
P01024	Complement C3	*C3*	62.7
P04264	Keratin, type II cytoskeletal 1	*KRT1*	59.3
Q9UGM3	Deleted in malignant brain tumors 1 protein	*DMBT1*	54.3
P00450	Ceruloplasmin	*CP*	50.3
Q16378	Proline-rich protein 4	*PRR4*	48.0
Q09666	AHNAK Nucleoprotein	*AHNAK*	46.0
P02538	Keratin, type II cytoskeletal 6A	*KRT6A*	45.0
P13645	Keratin, type I cytoskeletal 10	*KRT10*	44.3
O60437	Periplakin	*PPL*	40.7
P35579	Myosin-9	*MYH9*	39.3
P31025	Lipocalin-1	*LCN1*	36.7

Table S5. Biological processes associated with identified proteins in the whole strip (W), bulb (B), rest of the strip (R) and all identified proteins (W+B+R). The number of proteins involved and the percentage of all processes are ranked according to their value in W.

Biological Process	Section of Schirmer's strip W	B	R	W+B+R
Cellular process	525 (29.5%)	586 (30.6 %)	582 (30.2 %)	825 (31.3%)
Metabolic progress	350 (19.7%)	398 (20.8 %)	384 (19.9 %)	552 (20.9)
Biological regulation	227 (12.8%)	252 (13.1 %)	255 (13.2%)	344 (13%)
Response to stimuli	187 (10.5%)	178 (9.3 %)	185 (9.6 %)	236 (8.9%)
Localization	128(7.2 %)	138 (7.2 %)	143 (7.4%)	197 (7.5 %)
Signaling	88(4.9 %)	84 (4.4%)	88 (4.6%)	117(4.4%)
Immune system	84 (4.7%)	85 (4.4 %)	84 (4.4%)	102 (3.9%)
Multicellular organismal	51 (2.9%)	53(2.8%)	55 (2.9%)	72 (2.7%)
Interspecies interaction	46 (2.6%)	48 (2.5%)	48 (2.5%)	58 (2.2%)
Developmental process	39 (2.2%)	37 (1.9 %)	42 (2.2%)	54 (2.0%)
Locomotion	25 (1.4%)	26(1.4%)	25 (1.3%)	35 (1.3%)
Biological adhesion	20 (1.1%)	22 (1.1%)	22 (1.1%)	31(1.2%)
Growth	3 (0.2 %)	5 (0.3%)	3 (0.2%)	5 (0.2%)
Reproduction	2 (0.1%)	2 (0.1%)	3 (0.2%)	3 (0.1%)
Reproductive process	2 (0.1%)	2 (0.1 %)	3 (0.2%)	3 (1.0%)
Multi-organism process	1(0.1%)	1 (0.1%)	2 (0.1%)	2 (0.1%)
Biomineralization	1(0.1%)	1 (0.1%)	1(0.1%)	1 (0.1%)

Table S6. Molecular functions of identified proteins in the whole strip (W), bulb (B), rest of the strip (R) and all identified proteins (W+B+R). The number of proteins involved and the percentage of each group are ranked according to their value in W.

Molecular Function	Section of Schirmer's strip W	B	R	W+B+R
Binding	354 (43.4%)	418 (45.2%)	424 (46.1%)	565 (44%)
Catalytic activity	344 (42.2%)	360 (38.9%)	318 (34.6%)	492 (38.3%)
Molecular function regulator	52 (6.4%)	60 (6.5%)	69 (7.5%)	88 (6.9%)
Structural molecular	28 (3.4%)	53 (5.7%)	57 (6.2%)	65 (5.1%)
Transporter	12 (1.5%)	12 (1.3%)	23 (2.5%)	29 (2.3%)
Translation regulator	12 (1.5%)	9 (1%)	14 (1.5%)	19 (1.5%)
Molecular transducer	8 (1%)	5 (0.5%)	8 (0.9%)	11 (0.9%)
Molecular adaptor	6 (0.7%)	8 (0.9%)	6 (0.7%)	14 (1.1%)

Table S7. List of proteins identified in the chosen 6 signaling pathways. The number of corresponding proteins is shown between brackets.

	Batch	Gene name
Apoptosis (61)	W+B+R (29)	*PSMA1, PSMA2, PSMA3, PSMA4, PSMA5, PSMA6, PSMA7, PSMB1, PSMB2, PSMB3, PSMB4, PSMB6, PSMB8, PSMD4, PSMD10, PSME1, PSME2, CDH1, DBNL, DSG1, DYNLL1, DYNLL2, GSN, H1F0, HIST1H1C, HIST1H1E, SPTAN1, VIM, YWHAB*
	W+B (5)	*PSMB10, PSMB9, CASP3, CASP7, DSG2*
	W+R (5)	*PSMC1, PSMC5, PSMD2, PSMD7, KPNB1*
	B+R (5)	*PSMB5, PSMC3, HIST1H1B, PLEC, TRIM25*
	W (3)	*PSMD5, BID, TNFSF10*
	B (5)	*PSMB7, PSMD9, PSMF1, DFFA, FNTA*
	R (9)	*PSMC2, PSMC4, PSMC6, PSMD1, PSMD6, PSMD12, PSME3, LMNB1, STK24*
Complement (15)	W+B+R (11)	*C1R, C3, C4A, C4B, C5, C7, C8A, CFB, CFD, CFH, CFI*
	W+B (1)	*C6*
	W+R	/
	B+R (2)	*C1QC, C4BPA*
	W (0)	/
	B (1)	*C1QB*
	R (0)	/
IFNs Signaling (17)	W+B+R (5)	*HLA-A, HLA-B, HLA-C, FLNB, PSMB8*
	W+B (0)	/
	W+R (6)	*EIF4A1, EIF4A2, EIF4A3, EIF4G1, KPNB1, UBE2N*
	B+R (1)	*TRIM25*
	W (2)	*MAPK3, MX1*
	B (1)	*PIN1*
	R (2)	*EIF4E, HLA-E*
MMPs (19)	W+B+R (12)	*ACTN1, COL14A1, COL6A1, FLNA, FURIN, P4HB, PCOLCE, PLG, PLOD1, PPIB, TIMP1, TIMP2*
	W+B (3)	*ELANE, VASP, KLKB1*
	W+R (1)	*COL9A3*
	B+R (0)	/
	W (1)	*MMP7*
	B (2)	*MMP9, CTSG*

	R (0)	/
Cell Junction (10)	W+B+R (2)	*PVRL4, CDH1*
	W+B (0)	/
	W+R (2)	*CTNNA1, JUP*
	B+R (0)	/
	W (1)	*F11R*
	B (4)	*ACTN1, FLNA, VASP, PLEC*
	R (1)	*CTNND1*
Lipid Metabolism (21)	W+B+R (7)	*PLA2G2A, ARSA, CHKB, HEXA, HEXB, PSAP, SUMF2*
	W+B (1)	*GDP1L*
	W+R (5)	*CTSA, GBA, GLA, GLB1, NEU1*
	B+R (0)	/
	W (0)	/
	B (1)	*KDSR*
	R (7)	*PLA2G4B, PLA2G4D, PLA2G4E, HADHA, ASAH1, GM2A, NEU2*

Supplementary figures

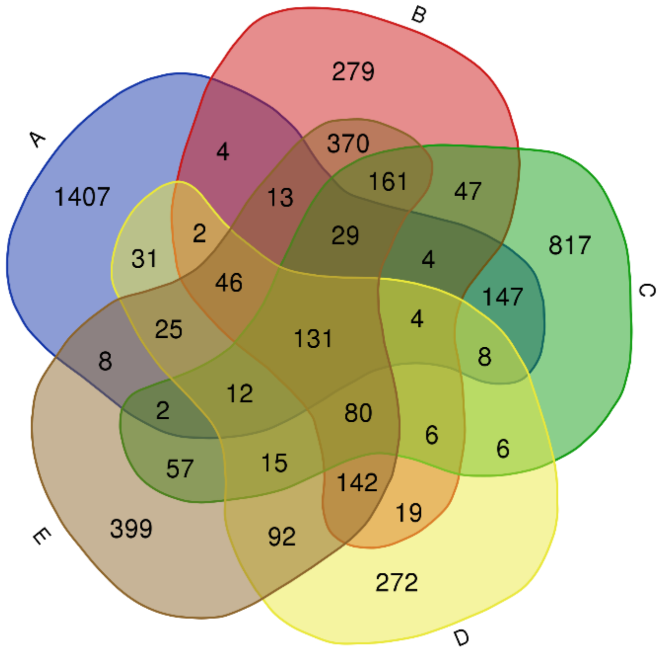

Figure S1. Venn diagram comparing four other comprehensive proteomics studies; (A) Kandhavelu *et al.*, (B) Dor *et al.*, and (C) Aass *et al.* (D) Ponzini *et al.* with (E) corresponding to our study.

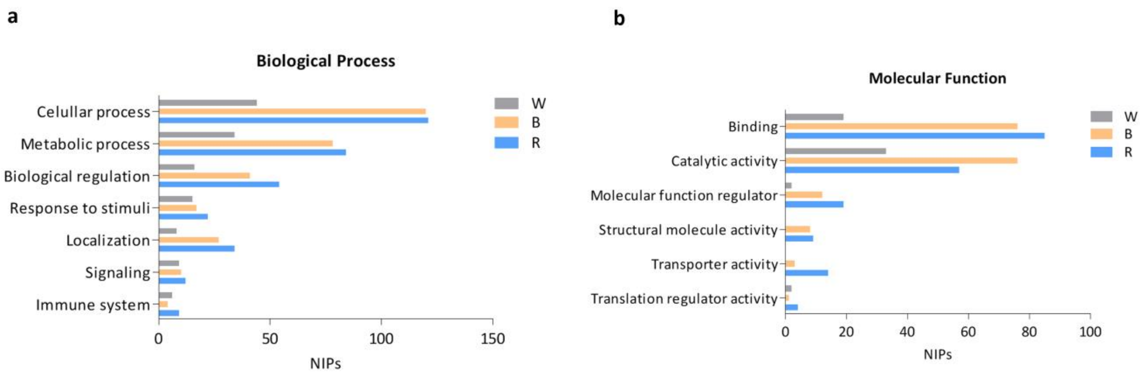

c

Protein Class	W	B	R
metabolite interconversion enzyme	14	61	33
protein modifying enzyme	15	16	16
cytoskeletal protein	3	12	14
defense/immunity protein	5	1	4
protein-binding activity modulator	0	11	9
translational protein	4	16	15
calcium-binding protein	2	1	1
chaperone	1	8	5
membrane traffic protein	3	7	8
extracellular matrix protein	1	1	2
scaffold/adaptor protein	0	8	4
nucleic acid metabolism protein	1	8	20
transfer/carrier protein	0	1	5
transporter	0	3	10
transmembrane signal receptor	2	2	1
intercellular signal molecule	0	3	5
cell adhesion molecule	0	1	1
chromatin/chromatin-binding	0	2	2
gene-specific transcriptional regulator	0	2	2
structural protein	1	0	2
viral or transposable element protein	0	0	1
cell junction protein	0	1	1

0	5	10	20	30	60

Figure S2. Functional analysis and structural classification of unique proteins in each batch. **a**. Subgroups of biological process, **b**.Subgroups of molecular function. **c**. The list and number of protein classes in each ScS section. The NIPs illustrates in the x-axis the number of proteins involved in (a) and (b).
